# Genome-wide association mapping for wheat morphometric seed traits in Iranian landraces and cultivars under rain-fed and well-watered conditions

**DOI:** 10.1038/s41598-022-22607-0

**Published:** 2022-10-25

**Authors:** Ehsan Rabieyan, Mohammad Reza Bihamta, Mohsen Esmaeilzadeh Moghaddam, Valiollah Mohammadi, Hadi Alipour

**Affiliations:** 1 Department of Agronomy and Plant Breeding, Faculty of Agricultural Sciences and Engineering, University of Tehran, Karaj, Iran; 2 Cereal Department, Seed and Plant Improvement Institute, AREEO, Karaj, Iran, Karaj, Iran; 3 Department of Plant Production and Genetics, Faculty of Agriculture, Urmia University, Urmia, Iran

**Keywords:** Genetic association study, Genetic markers

## Abstract

Seed traits in bread wheat are valuable to breeders and farmers, thus it is important exploring putative QTLs responsible for key traits to be used in breeding programs. GWAS was carried out using 298 bread wheat landraces and cultivars from Iran to uncover the genetic basis of seed characteristics in both rain-fed and well-watered environments. The analyses of linkage disequilibrium (LD) between marker pairs showed that the largest number of significant LDs in landraces (427,017) and cultivars (370,359) was recorded in genome B, and the strongest LD was identified on chromosome 4A (0.318). LD decay was higher in the B and A genomes, compared to the D genome. Mapping by using mrMLM (LOD > 3) and MLM (0.05/m, Bonferroni) led to 246 and 67 marker-trait associations (MTAs) under rain-fed, as well as 257 and 74 MTAs under well-watered conditions, respectively. The study found that 3VmrMLM correctly detected all types of loci and estimated their effects in an unbiased manner, with high power and accuracy and a low false positive rate, which led to the identification of 140 MTAs (LOD > 3) in all environments. Gene ontology revealed that 10 and 10 MTAs were found in protein-coding regions for rain-fed and well-watered conditions, respectively. The findings suggest that landraces studied in Iranian bread wheat germplasm possess valuable alleles, which are responsive to water-limited conditions. MTAs uncovered in this study can be exploited in the genome-mediated development of novel wheat cultivars.

## Introduction

Bread wheat (*Triticum aestivum* L.) is a strategic cereal worldwide and can feed approximately 30% of the global population and provide 25% of the calorie consumed by humans^1^. Owing to rapid population growth, climate change, and abiotic stress incidence in the world, wheat productivity needs a 2.5% of yield increase yearly. Therefore, to meet future demand, plant breeders face the challenge of increasing wheat production up to 70% by the 2050s^2^. Drought stress adversely influences wheat productivity by disrupting a variety of bio-physiological and metabolic activities, and thereby giving rise to yield loss by a diminution in biomass^3^. Seed morphometric properties are explored as basic parameters in digital seed analysis^4^, enhancing the understanding of seed response to drought stress and providing data for research on wheat breeding in water-limited conditions^5–7^. There are only a few reports on investigating seed physical traits in the previous researches^8^, and such data have been focused on energy dissipation (thermodynamic) or shape, volume, surface area, sphericity, aspect ratio, density, and moisture (dimensional) properties. Seed physical traits, such as shape and size, are found effective for grain storage and processing. These features may be helpful, among others, for food scientists, processors, and engineers. The composition of the seed is influenced by the seed number, cultivar, water availability, temperature, light, and maturity^9^.

Genomics-by-sequencing (GBS) is a method for evaluating genetic variation and discovering new markers based on the advent of next-generation sequencing technologies^10^. This approach has been used to discover the complicated agronomical properties of wheat using molecular markers such as single nucleotide polymorphisms (SNP). They have also been recognized as key elements in genome-wide association studies^11^. This approach is aimed at detecting genomic regions that are either QTLs, genes, or markers related to important traits for gene introgression, gene discovery, or marker-assisted breeding^12^. Genetic markers detected by GWAS enable the dissection of genetic structure and diversity across many loci. This can enable wheat breeders to discover and use genomic loci controlling drought tolerance^13^.

Exploring the genetic basis of complicated quantitative traits by innovative technologies is critical to wheat breeding programs^14^. Genome-wide association mapping (GWAS), an efficient approach to dissecting the genetic foundation of complex traits, first genotypes a large collection of accessions with a lot of single-nucleotide polymorphisms (SNPs) distributed throughout the genome and then tests their associations with agronomic traits^15^. Association mapping has been successfully utilized to evaluate several agronomic traits in a range of plants/crops, including alfalfa^16^, sorghum^17^, soybean^18^, maize^19^, and rice^20^. Although GWAS has been widely adopted to examine agronomic characteristics in wheat, only a few studies used this approach for seed-related properties in drought-stressed wheat genotypes. In an attempt, Rahimi et al.^21^ demonstrated that bread wheat landraces from Iran possess favorable alleles, which are adaptive to water deficit. They also observed marker-trait associations (MTAs) within protein-coding regions that can be used in the molecular breeding of novel wheat cultivars. Such studies also provide important data about MTAs, which can assist plant breeders in the marker-assisted selection schedules^22^.

The objective of this study was to perform a genome-wide association analysis for seed morphometric traits in Iranian bread wheat. Seed morphometric traits were utilized in association studies to uncover putative QTLs responsible for key seed traits in water-limited conditions.

## Results

### Phenotypic data summary

**Figure 1:**
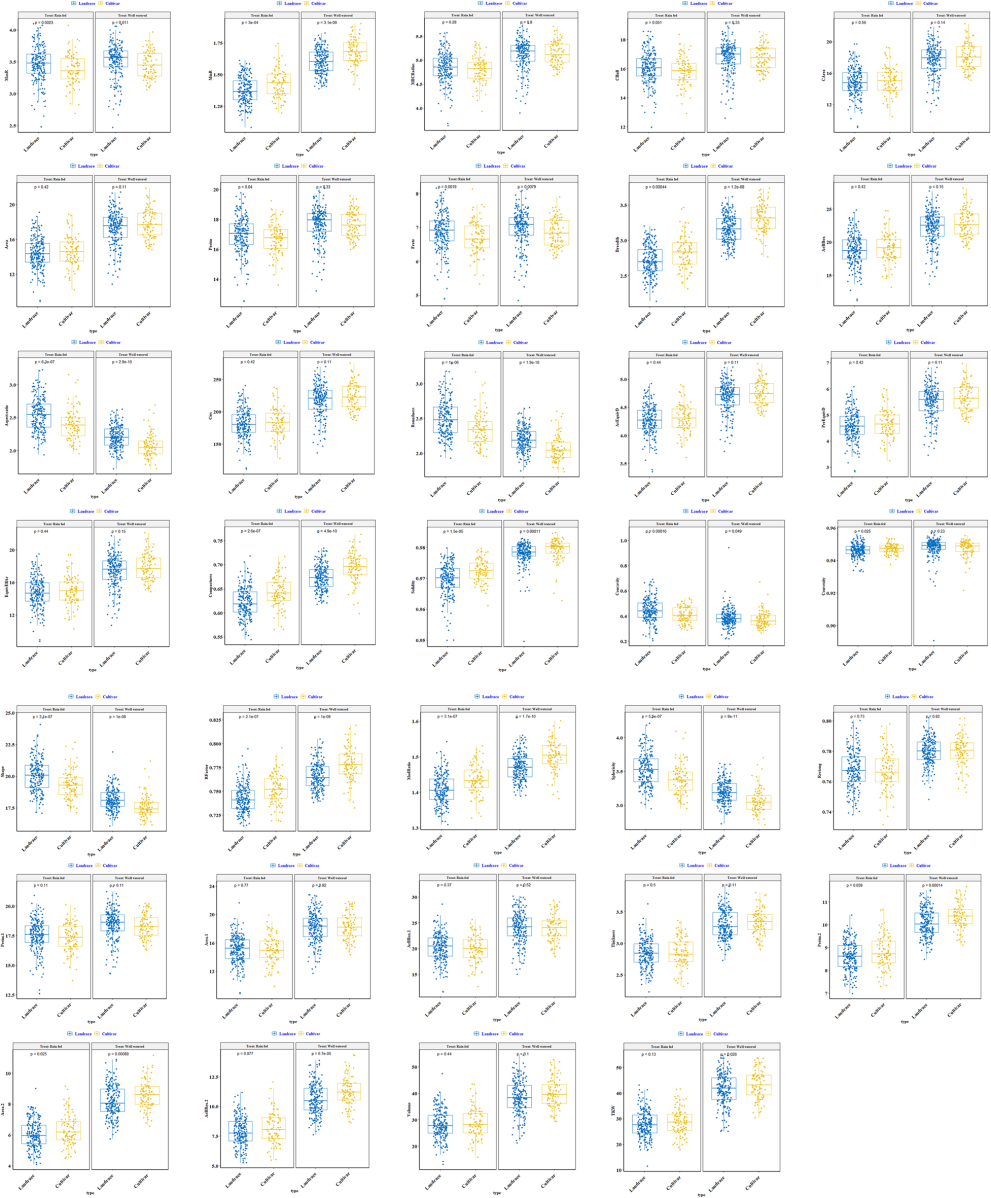
Box-plot representation of the distribution for a total of 34 morphometric seed traits for Iranian wheat landraces and cultivars in the well-watered and rain-fed environments.

The effects of genotype and genotype × environment for seed morphometric traits in the whole population were significant at 0.001 probability level (Table 1). The results of the box plot of 34 morphometric traits of wheat seeds for cultivars and native landraces in favorable conditions (well-watered) and stress (rain-fed) are shown in Fig. 1. The means of all traits under stress conditions decreased compared to normal conditions in both cultivars and native landraces. In both environments, the highest length (Feret) and width (Breadth) were found in landraces and cultivars, respectively. There was no significant difference between cultivars and landraces for the most important morphometric traits of seeds, i.e. Area, Area.1, Area.2, Volume, thickness, and 1000-kernel weight (TKW). Overall, the diversity and distribution among native landraces were higher than those of cultivars in both well-watered and rain-fed environments.

**Table 1:** Mean, coefficient of variation (CV), broad sense heritability (H^2^), and combined analysis of variance based on studied traits in 298 Iranian wheat landraces and cultivars.

Trait	Well-watered							Rain-fed						
	Mean	CV (%)	H^2^	Mean squares				Mean	CV (%)	H^2^	Mean squares			
				Env	Rep (env)	Gen	Gen × Env				Env	Rep (Env)	Gen	Gen × Env
ArBBox	22.37	6.543	0.831	*	**	***	***	18.89	7.926	0.732	**	**	***	***
ArBBox_1	24.07	7.595	0.848	*	*	***	***	20.16	8.676	0.686	**	**	***	***
ArBBox_2	10.79	8.027	0.801	*	*	***	***	7.963	10.43	0.660	**	**	***	***
Area	17.44	6.461	0.826	*	*	***	***	14.49	7.811	0.719	**	**	***	***
Area_1	18.22	7.495	0.839	*	*	***	***	15.06	8.419	0.681	**	**	***	***
Area_2	8.322	7.965	0.808	*	*	***	***	6.133	10.03	0.619	**	**	***	***
ArEquivD	4.702	3.260	0.835	*	*	***	***	4.283	3.910	0.738	**	**	***	***
Aspectratio	2.166	4.170	0.881	*	*	***	***	2.504	5.253	0.843	**	**	***	***
Breadth	3.213	4.454	0.799	*	*	***	***	2.750	5.815	0.701	**	**	***	***
CArea	17.82	6.411	0.830	*	*	***	***	14.93	7.699	0.728	**	**	***	***
CHull	16.74	3.030	0.878	ns	ns	***	***	15.97	3.335	0.840	ns	ns	***	***
Circ	219.1	6.461	0.826	*	**	***	***	182.1	7.811	0.719	**	**	***	***
Compactness	0.680	2.030	0.882	ns	ns	***	***	0.629	2.478	0.830	ns	*	***	***
Concavity	0.382	22.18	0.504	*	*	***	***	0.437	12.92	0.631	**	**	***	***
Convexity	0.948	0.865	0.374	*	**	***	***	0.947	0.415	0.519	**	**	***	***
EquivEllAr	17.53	6.561	0.830	*	**	***	***	14.79	8.001	0.732	**	**	***	***
Frete	6.931	3.237	0.901	ns	ns	***	***	6.829	3.414	0.869	*	*	***	***
MaxR	3.495	3.151	0.899	*	**	***	***	3.439	3.334	0.869	**	**	***	***
MBCRadius	5.123	3.057	0.868	ns	ns	***	***	4.831	3.485	0.811	*	*	***	***
MinR	1.628	4.357	0.803	ns	ns	***	***	1.392	5.634	0.706	ns	ns	***	***
ModRatio	1.481	1.401	0.890	ns	ns	***	***	1.417	1.568	0.842	*	*	***	***
PerEquivD	5.551	6.461	0.826	ns	ns	***	***	4.613	7.811	0.719	ns	*	***	***
Perim	17.67	3.292	0.869	ns	ns	***	***	16.88	3.410	0.837	ns	*	***	***
Perim_1	18.41	3.353	0.879	ns	ns	***	***	17.46	4.073	0.794	*	*	***	***
Perim_2	10.17	4.135	0.800	ns	*	***	***	8.681	5.201	0.672	*	*	***	***
Rectang	0.780	1.207	0.488	*	**	***	***	0.768	1.332	0.707	ns	ns	***	***
RFactor	0.770	1.049	0.882	*	**	***	***	0.745	1.077	0.828	*	*	***	***
Roundness	2.156	4.605	0.871	*	**	***	***	2.456	5.872	0.846	*	*	***	***
Shape	17.99	3.778	0.770	ns	*	***	***	19.82	3.150	0.820	*	*	***	***
Solidity	0.978	0.463	0.416	**	**	***	***	0.970	0.381	0.556	**	**	***	***
Sphericity	3.155	2.744	0.880	ns	*	***	***	3.490	3.567	0.849	*	*	***	***
Thickness	3.312	5.445	0.801	ns	*	***	***	2.847	6.379	0.607	*	**	***	***
TKW	42.10	1.193	0.796	*	ns	***	***	28.18	1.955	0.591	*	ns	***	***
Volume	38.92	8.379	0.837	***	***	***	***	28.38	10.71	0.688	**	**	***	***

*, **, *** and ^ns^ are significant at the probability levels of 5%, 1%, 0.1%, and non-significant, respectively.

**Figure 2:**
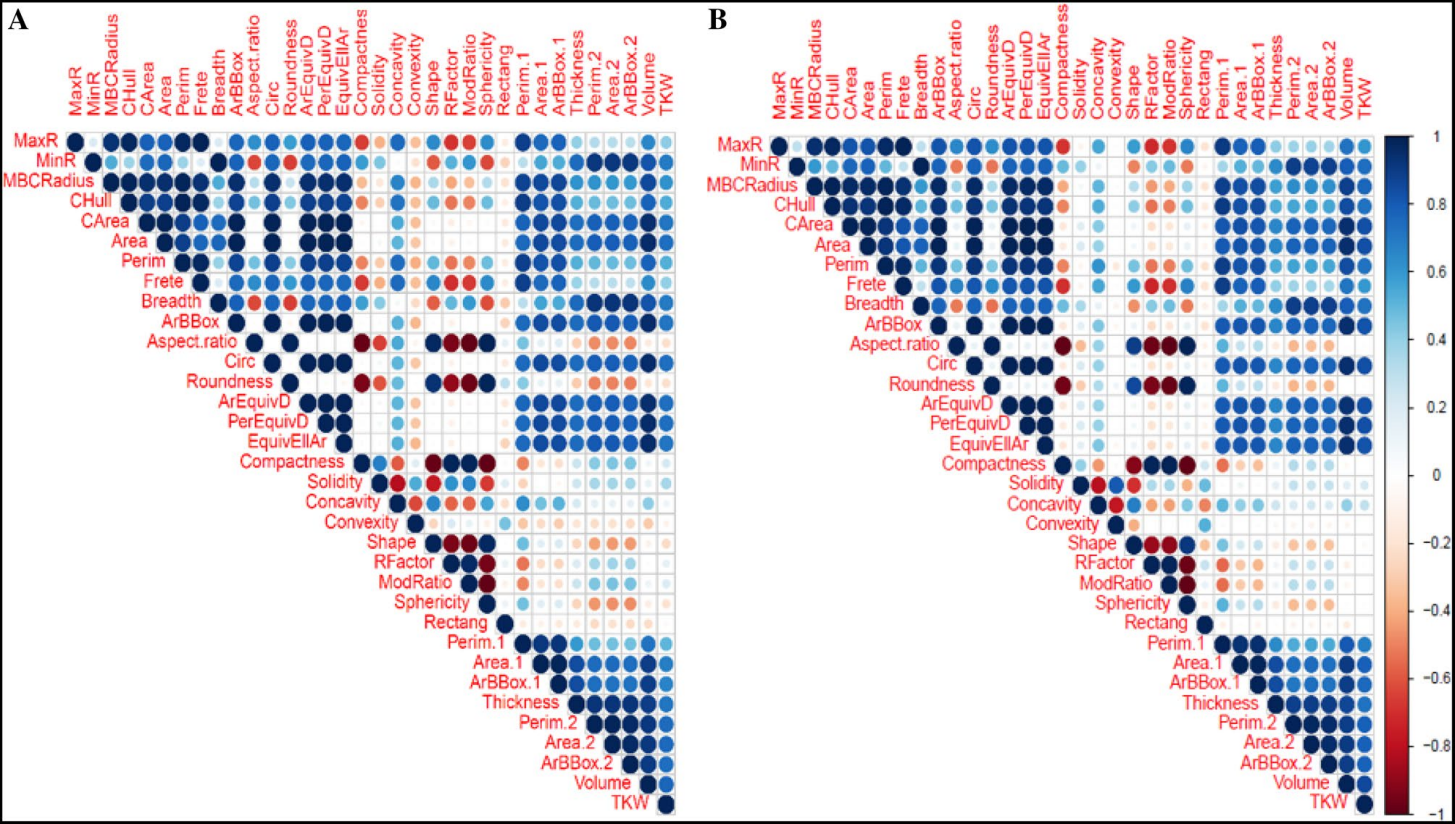
Correlation coefficients between morphometric seed traits for Iranian wheat landraces and cultivars in the well-watered (**A**) and rain-fed (**B**) environments.

The highest correlation was observed between TKW and volume under stress (r = 0.76**) and normal (r = 0.85**) conditions. There was also a high correlation between circ and TKW in rain-fed (r = 0.73**) and well-watered (r = 0.83**) environments, which indicates that the more round the seed, the heavier it will be (Fig. 2; Supplementary Table 1).

### Assessment of SNPs

**Figure 3:**
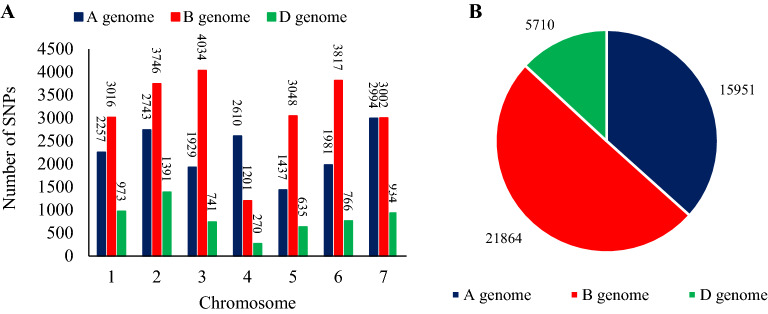
Number of imputed SNPs used in wheat chromosomes (**A**) and genomes (**B**).

The number of imputed SNPs includes 15,951, 21,864, and 5710 markers for genomes A, B, and D, respectively, including 36.7, 50.2, and 13.1% of total SNPs (Fig. 3A). The highest number of markers used in all chromosomes except chromosome 4 is related to genome A. The highest number of markers 4034 is related to chromosome 3A and the lowest number of markers 270 is related to chromosome 4D (Fig. 3B).

### Linkage disequilibrium (LD)

LD assessment indicated that this indicator varies between chromosomes and across each chromosome and it usually decreases with rising distances between SNP locations. A total of 1,858,425 marker pairs with r^2^ = 0.211 were identified in cultivars, of which 700,991 (37.72%) harbored significant linkages at *P* < 0.001. The strongest LD was recorded between marker pairs on chromosome 4A (r^2^ = 0.367). Based on the observations, most of the significant marker pairs were found at a distance of < 10 cM. Genomes D and B possessed the lowest and highest number of significant marker pairs (63,924) and (370,359), respectively. A similar analysis on landraces identified a total of 1,867,575 marker pairs with r^2^ = 0.182, of which 847,725 (45.39%) harbored significant linkages at *P* < 0.001. Similar to cultivars, marker pairs on chromosome 4A showed the strongest LD (r^2^ = 0.369). Moreover, most of the significant marker pairs were found at a distance of < 10 cM. Genomes D and B possessed the lowest and highest number of marker pairs (92,702 and 427,017), respectively (Supplementary Table 2).

### Population structure and Kinship matrix

**Figure 4:**
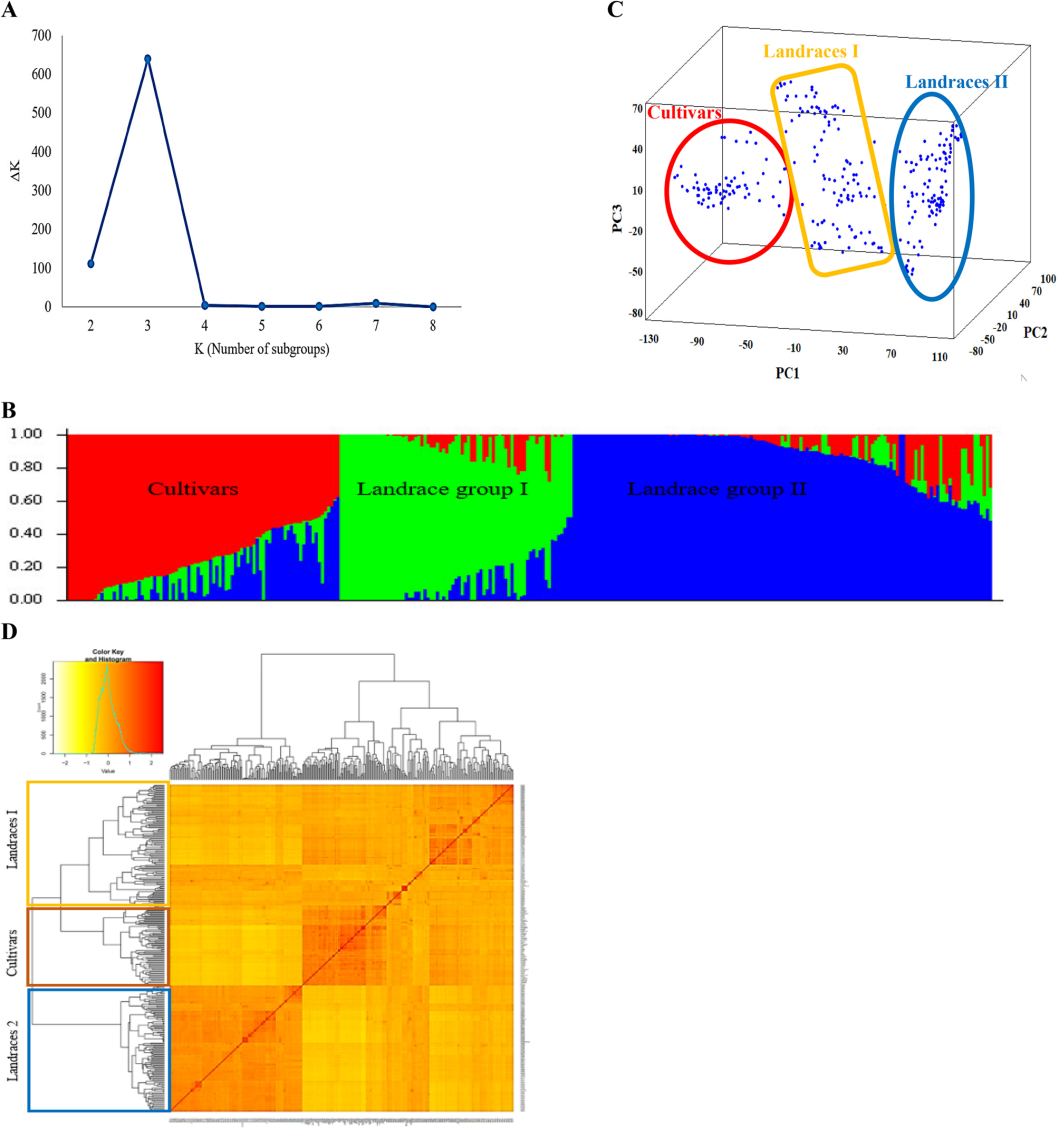
Determination of subpopulations number in wheat genotypes based on ΔK values (**A**), A structure plot of the 298 wheat genotypes and landraces determined by K = 3 (**B**). Principle component analysis (PCA) for a total of 298 Iranian bread wheat accessions (**C**). Cluster analysis using Kinship matrix of imputed data for Iranian wheat accessions (**D**).

The genetic relationship of accessions in the wheat population was assayed via the Kinship matrix derived from imputed SNPs. Population structure analysis indicated the highest value of ΔK for K = 3 (Fig. 4A,B). The estimated principal components for the population revealed that PC1, PC2, and PC3 explain 16.94, 6.34, and 2.30% of genotypic variations, respectively (Fig. 4C). As expected, a population structure was identified in the Iranian wheat landraces, with the first five eigenvalues accounting for 30.50% of genetic diversity.

The clustering analysis determined three major groups with different levels of admixture, where Group I consists of 6 cultivars and 107 landraces, Group II includes 4 landraces and 70 cultivars, Group III includes 14 cultivars, and 97 landraces (Fig. 4D). From the imputed SNP data, a total of 19 Cultivars appeared to be mixed with the two native landrace groups. The admixed Cultivars originated include the Sivand, Neishabour, Ghods, Azadi, Mahdavi, 4820, and Shahi. A neighbor-joining tree indicated that both cultivars and landraces were divided into two groups based on the imputed SNPs (Supplementary Fig. 1). In an analysis based on cultivars, two groups with 42 and 48 accessions were obtained. Native landraces were also divided into two groups with 98 and 110 accessions. The reason for each group's location can be due to the characteristics of the parents and the place where they came from.

### Genome-wide association studies for morphometric seed traits using mrMLM, 3VmrMLM, and MLM

**Figure 5:**
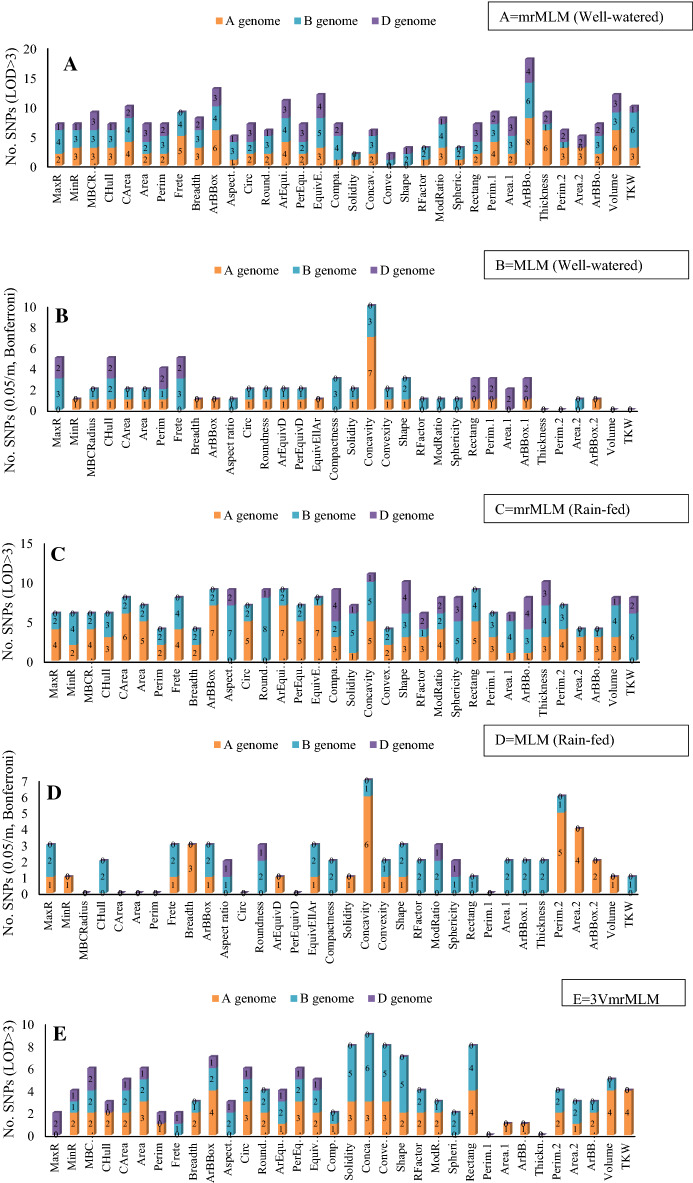
GWAS results for seed traits in Iranian wheat landraces and cultivars. (**A**) mrMLM (Well-watered), (**B**) MLM (Well-watered), (**C**) mrMLM (Rain-fed), (**D**) MLM (Rain-fed), (**E**) 3VmrMLM.

A total of 257 and 74 MTAs were identified by mrMLM and MLM models under well-watered conditions, respectively, using the imputed SNPs at a significance value of LOD > 3 (mrMLM) and 0.05/m (MLM). Of the total MTAs in the mrMLM method, 95, 99, and 63 MTAs were related to genomes A, B, and D, respectively. Out of 74 MTAs in the MLM method, 27, 31, and 16 MTAs belonged to genomes A, B, and D, respectively. Genome B with 38.5% (mrMLM) and 41.9% (MLM) had the highest number of significant MTAs. Therefore, the mrMLM approach led to the most MATs. The number of significant MTAs for Frete, Breadth, Thickness, Area, Perim, Circ, Volume, and TKW traits by using the mrMLM method were 9, 8, 9, 7, 7, 7, 12, and 10, respectively, and according to the MLM method were 5, 1, 0, 2, 4 2, 0, and zero, respectively. Based on mrMLM and MLM methods, the highest number of significant MTAs was related to ArBBox.1 and Concavity (18 and 10 MTAs, respectively) (Fig. 5A,B).

**Figure 6:**
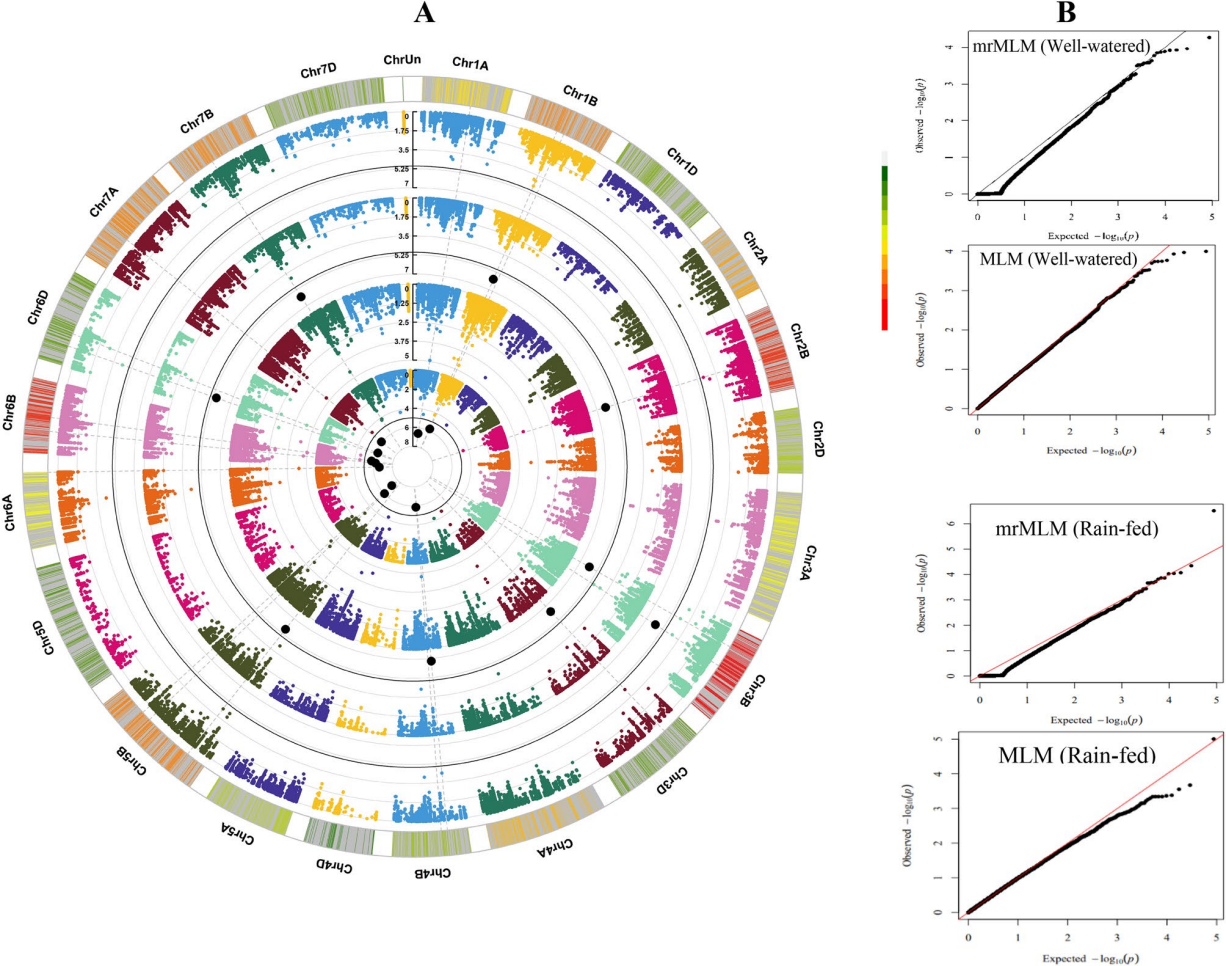
Circular Manhattan (**A**) and QQ-plots (**B**) to draw common regions associated with TKW in Iranian wheat landraces and cultivars. Inner to outer circles represents average trait for the mrMLM and MLM methods in the well-watered and rain-fed environments, respectively. The chromosomes are plotted at the outmost circle where thin dotted blue and red lines indicate significant levels at *P*-value < 0.00001 (0.05/m, Bonferroni), respectively. Black dots indicate genome-wide significantly associated SNPs at *P*-value < 0.00001 (0.05/m, Bonferroni)), probability levels. The scale between ChrUn and Chr1A indicates − log10 (p) values. Colored boxes outside on the top right side indicate SNP density across the genome where green to red indicates less dense to dense.

More significant MTAs were identified in rain-fed than well-irrigated conditions, i.e., a total of 246 and 67 MTAs were recorded based on mrMLM and MLM methods, respectively. Of these MTAs, 110, 105, and 31 mrMLM-based MTAs, as well as 30, 33, and 4 MLM-based MTAs were related to genomes A, B, and D, respectively. Genome A and B had the highest percentage of significant MTAs with 44.7% and 49.6% based on mrMLM and MLM, respectively. The number of significant MTAs for Frete, Breadth, Thickness, Area, Perim, Circ, Volume, and TKW traits according to the mrMLM method were 8, 4, 10, 7, 4, 7, 8, and 8, respectively, and according to the MLM method were 3, 3, 2, 0, 0, 0, 1, and 1, respectively. Based on mrMLM and MLM methods, the highest number of significant MTAs were related to Concavity (11 and 7 MTAs, respectively) (Fig. 5C,D). Circular Manhattan plots were plotted for common regions associated with seed traits (Fig. 6; Supplementary Fig. 2).

In this study, we adopted a three-variance component mixed model method, 3VmrMLM, for detecting QTNs and QTN-by-environment (QEIs). A total of 140 MTAs were identified by 3VmrMLM model, using the imputed SNPs at a significance value of LOD > 3. A total of 64, 60, and 16 MTAs based on 3VmrMLM, were related to genomes A, B, and D, respectively. Genome A with 45.7% had the highest number of significant MTAs. Therefore, the 3VmrMLM approach led to the most MATs. The number of significant MTAs for Frete, Breadth, Thickness, Area, Circ, Volume, and TKW traits by using the mrMLM method were 2, 3, 0, 6, 6, 5, and 4, respectively (Fig. 5E). QTN-by-environment interactions using 3VmrMLM for Area, Perim, Frete, Breadth, and TKW are reported in Table 2 and other traits in Supplementary Table 3.

**Table 2:** A summary of QTN-by-environment interactions for some seed traits of Iranian wheat using 3VmrMLM.

Trait name	Marker	Sequence	Chro	Position (cM)	LOD (QE)	add*env1	dom*env1	add*env2	dom*env2	add*env3	dom*env3	add*env4	dom*env4	variance	r^2^ (%)	P-value	Method	Transcript ID	Biological process	Molecular process	Cellular component
Area	rs38918	TGCAGCTGCCTGCTCATCCTCACCATCTCAATCTGTGTACGAGTAGATCCCATGGTTTACCAGT	2A	11.39	22.1318	− 0.3807	− 0.5515	− 0.2525	− 0.6141	0.3785	0.3653	0.2548	0.8002	0.1164	2.0318	9.99E−20	MLM	TraesCS2A02G045800		Protein binding	
	rs47935	TGCAGGCGCCACTTATCCTAATCGGCCAATAAGGCCAACTCCAACGCGCACCCCCATCATGTCT	2B	59.184	13.7438	0.3024	0.3799	0.2175	0.4805	− 0.296	− 0.4631	− 0.224	− 0.3972	0.0711	1.2412	9.63E−12	MLM	–	–	–	–
	rs24480	TGCAGCCCTGATGGCTCTGCTGAAACATTCTGGACCCGAAACATGAACTCTACTGCCAGTCGGA	2B	111.506	10.603	− 0.0823	0.0286	− 0.3229	− 0.5606	0.1143	− 0.2102	0.2909	0.7421	0.0545	0.9517	8.08E−09	–	–	–	–	–
	rs38828	TGCAGCTGCCGAGGTACGCTTCGACTTGAAGTTCGAGAATGTGCCCTTGGTGATCCTCTACATG	4A	147.563	6.9031	0.1636	0.0899	0.2148	− 0.0506	− 0.2024	0.0791	− 0.1759	− 0.1183	0.0352	0.6152	1.79E−05	–	–	–	–	–
	rs62825	TGCAGTTCAAAGGAGTTCAATGGAAAGGGCGGGTCGGGGCTTATAAACAGGTCCTGCCGCTCCT	7A	0.5685	5.5317	0.2002	0.0931	0.1358	− 0.1428	− 0.1489	− 0.0209	− 0.1871	0.0706	0.0282	0.4917	0.000279	–	–	–	–	–
	rs5421	TGCAGAGAGAAAAAGCACCATCATCAAAGCAATATCCAGCAATAACCAATGAAAAAAAACCCAC	7D	71.943	14.4177	0.3461	− 0.7677	0.1448	0.2718	− 0.1211	0.749	− 0.3697	− 0.253	0.0747	1.3038	2.24E−12	–	–	–	–	–
Frete	rs20215	TGCAGCATTATTGCTTGTTATGTCGGTGTCCCTTGCCGCACTAGAGGGTGTTCATGGCGTTTGC	2B	67.141	13.8519	-0.0533	-0.0424	-0.0708	0.0811	0.0623	0.0422	0.0618	− 0.0809	0.0039	1.335	7.63E−12	–	TraesCS2B02G461219	Systemic acquired resistance	Fatty acid binding	
	rs53985	TGCAGGTGCCGTGGGTGCAAAAACAACTCCTGCAACATTTTCATCCGTGCTTAAAGCTCTAAAT	7D	150.625	15.0054	0.0994	–	0.0148	–	− 0.0453	–	− 0.0689	–	0.0042	1.4494	6.65E−15	–	–	–	–	–
Breadth	rs2102	TGCAGAAGCTTGGGATACTTGGAGTATAGCAAAGGGCGGCATGTGTATAAAATTTAGTTTTGTA	1A	34.278	6.3891	0.005	− 0.1148	0.0005	− 0.191	0.0018	0.166	− 0.0072	0.1399	0.0006	0.5544	5.06E−05	–	–	–	–	–
	rs5425	TGCAGAGAGAAAGAGGGGTTTCGGGCTACGCGGTTAACCGTTGGAGTACCAAACGACCTCCAAA	2B	59.184	6.0309	0.0295	0.1095	0.0135	0.0079	− 0.0208	− 0.0358	− 0.0222	− 0.0816	0.0006	0.5229	0.000104	–	–	–	–	–
	rs33742	TGCAGCGTGCCTTGCAATGGCGTGCGCAACCTACAAGCAAAATTACACAAGATGCCAAATTACA	6A	99.391	10.1792	− 0.0216	− 0.023	− 0.0381	0.0122	0.0212	− 0.0233	0.0384	0.034	0.0009	0.8897	1.98E−08	mrMLM	TraesCS6A02G402300	Proteolysis	–	–
	rs35884	TGCAGCTCACGTAGAAGGAGACCCGACCGACAGCGCGATTCGCAAGACAGTCGACGAGCGCTTT	1B	75.148	16.9999	− 0.0001	0.0074	− 0.0001	− 0.0222	0.0004	0.0071	− 0.0002	0.0077	0	3.2964	8.08E−15	–	–	–	–	–
	rs13332	TGCAGCACAACAACACAAGATGCGCCACAAAGATTAGAAACAGGCCCGAAAGCAAGCAAGTAAA	3A	43.437	6.3256	− 0.0002	− 0.0086	− 0.0001	0.0004	0.0001	0.0025	0.0002	0.0058	0	1.2014	5.75E−05	–	TraesCS3A02G084300	snRNA import into nucleus	-	Nucleus, cytoplasm
	rs7643	TGCAGATACCCCTACCGACACCATGTCTCTTTCTTACTTATTTCTTAATCAATTGTGATCTTTT	3B	22.764	39.7108	0.0028	− 0.0007	− 0.0001	0.0022	− 0.0011	0.0005	− 0.0015	− 0.002	0	8.052	8.34E−37	–	–	–	–	–
	rs10599	TGCAGATTTTGGGTTTAGTCCCACTTTTAACATTGTAGATTTGAAGCCTTATTTGGGTGACGAG	3B	48.935	12.9176	− 0.0008	− 0.0239	0.0002	0.0086	0.0004	0.0071	0.0003	0.0082	0	2.485	5.73E−11	–	–	–	–	–
	rs15115	TGCAGCACGGAAGGAGAGGGCCTAGCCACCTGGTACACTTGCTGGGGCGCGGGCAGACGCGGAC	5B	145.902	12.7877	− 0.0003	− 0.015	0	0.0087	0.0003	0.0037	0	0.0025	0	2.4594	7.57E−11	–	–	–	–	–
	rs31905	TGCAGCGGCCGCCTAGACACCCAGGGCCAGGGATCTGACGGTTAGCTTTAGCTTAATCGAACGG	5B	145.902	6.0769	0.0004	− 0.0065	0.0003	0.0023	− 0.0005	0.002	− 0.0002	0.0022	0	1.1536	9.46E−05	–	–	–	–	–
	rs49346	TGCAGGCTTTCCAGCTGAACAGCTGAATCACAGGGTATACACTGCGTTAGGATTCTTAACTTTG	7A	69.63	11.3531	0	− 0.0148	0.0004	0.0133	− 0.0002	-0.0018	− 0.0002	0.0034	0	2.1774	1.64E−09	–	–	–	–	–
TKW	rs56585	TGCAGTACTTGCGTCAAACCGCCTCCATCTTGCACTGCATCAGGGCCTCCGTCTCTGCTGGCGG	1A	44.512	25.3101	0.9973	1.4715	1.0725	1.2849	− 1.033	− 1.3295	− 1.0368	− 1.427	1.0857	1.3583	8.62E−23	MLM	–	–	–	–
	rs29065	TGCAGCGATATCTCCCTACAACAATCAGACGGCTCCACGACGGGATGGCGGCTCCTCCATGAGG	6A	25.146	11.9358	− 0.1777	− 3.2922	− 0.0918	-3.3345	0.0989	3.5286	0.1706	3.0981	0.4988	0.624	4.71E−10	–	–	–	–	–
	rs31607	TGCAGCGGCAAGAACATGGATTAGTCCTTCCAGGAGACAGAAACCACACGGATCGATCGACCCG	6A	99.391	15.5325	− 0.8601	− 0.0068	− 0.8259	0.0254	0.8562	− 0.0187	0.8299	0.0001	0.6537	0.8178	1.99E−13	MLM	TraesCS6A02G399400	Phosphorelay signal transduction system, signal transduction, response to ethylene	Protein binding, phosphorelay sensor kinase activity, metal ion binding, ethylene binding	Endoplasmic reticulum membrane, integral component of membrane
	rs56117	TGCAGTACAATTTTCCCACGGCCACACCAGCCAACCAGGATACAACACAGGTAACGCAAGGAAA	7A	63.946	14.2069	− 0.577	3.2412	− 0.5815	3.0985	0.5794	− 3.2171	0.5791	− 3.1226	0.5963	0.746	3.54E−12	–	TraesCS7B02G121200	Protein complex oligomerization, iron ion homeostasis	ATPase activator activity, chaperone binding	–

### Gene ontology

The markers with the highest significance and pleiotropy were studied in more detail. A total of 10 high-significance markers were identified in well-watered plants, most of which were located on chromosomes 1A, 1D, 2B, 2D, 3B, 4A, and 7A. Genes encoding proteins from MTAs were involved in molecular/biological processes such as metal ion binding, ATP binding, calcium ion binding, DNA binding, positive regulation of protein catabolic process, protein ubiquitination, ionotropic glutamate receptor, ligand-gated ion channel, lipid binding, and transport, protein phosphorylation, protein kinase, oxidation–reduction, and lipid biosynthesis (Table 3). In the rain-fed plants, 10 high-significance markers were identified with the highest pleiotropy, most of those were located on the wheat chromosomes 1A, 1B, 2A, 2D, 3B, 4A, and 6A. Protein-encoded genes from MTAs were responsible for molecular/biological processes such as metal ion binding, Fe ion binding, lipid binding, and transport, oxidation–reduction, lipid biosynthesis, oxidoreductase activity, and DNA-binding transcription factor (Table 3).

**Table 3:** Description of expected MTAs by using the imputed SNPs for seed morphometric traits of Iranian wheat accessions exposed to the well-watered and rain-fed environments.

No.	Environments	SNP	Sequence	Chromosome	Position	Trait- Index	Transcript ID	Biological process	Molecular process	Cellular component	P-value	QTL reported in previous studies	Identified genes in QTL region in Triticum species
1	Well-watered	rs5540	TGCAGAGAGGAAACGGTGGCCGTGCCTGATATCTCGGCGTTGGCTCTCGCTTTGAGGCTCCTGA	1A	3415	Concavity, Convexity, Perim	TraesCS1A02G008500	-	Acyltransferase activity, transferring groups other than amino-acyl groups	–	0.0000946	27	Gamma gliadin-A1, gamma gliadin-A3, gamma gliadin-A4, and LMW-A2 genes
2		rs14628	TGCAGCACCTCTTTACTGAAGCTAGTGGTACTGCTCGCTGCGTATGATGTGGACCGCCATGGCA	1A	31,851	Rfactor, Roundness, ModRatio	TraesCS1A02G399700	transcription, DNA-templated, regulation of transcription, DNA-templated	DNA binding	Nucleus	0.00000146	28	B3 domain-containing protein Os03g0619600-like (LOC123183123)
3		rs15519	TGCAGCACTCTGCAAGAAAAACGTCAAAGTAAGAACCACCTACCCACATCTGCTCCAATTCAAA	1D	47,767	Feret, Perim,Volume, TKW, CArea, Chull, Area, Circ, PerEquivD, ArEquivD, MBCRadius, ArBBox, EquivEllAr	TraesCS1D02G147800	Lipid biosynthetic process	Iron ion binding, oxidoreductase activity	Integral component of membrane	0.0000297551	29	Very-long-chain aldehyde decarbonylase GL1-5-like (LOC123181004)
4		rs2432	TGCAGAATAAGAATATTAAGTTGATCAACATCCAGATCAACGCGCCCGAGAACAGCCCAAACAC	2B	69,414	Perim.1	TraesCS2B02G491300	Carbohydrate metabolic process, cell wall organization	Polygalacturonase activity	Extracellular region	0.000441697	27	Exopolygalacturonase-like (LOC123042122)
5		rs59777	TGCAGTCTTTCAGAAGTGCAGATGTAAACGTATTGCTATATCAGTGGTTTGAACTACATGGTAA	2D	58,883	Area.2, Perim.2, Feret, Volume, TKW	TraesCS2D02G152500	Protein ubiquitination, positive regulation of protein catabolic process	Protein binding	–	0.0000444241	29,30,31,32	Exopolygalacturonase-like (LOC123042122)
6		rs51999	TGCAGGGTTCTGGTGGCGATGCCGACCGTCGTCGAGAGGTTCGTGGACTGGGCGGTGTTGTGGC	3B	67,127	ArBBox, EquivEllAr, MBCRadius, Perim, Chull, Carea, Area, Circ, PerEquivD, ArEquivD, Perim.1, MaxR, Frete	TraesCS2D02G480600	–	–	Integral component of membrane	0.000248572	27,33	HVA22-like protein i (LOC123103004)
7		rs7487	TGCAGATAAACAGCATCAGCTCAGTTCACGGATCGATCGAGCATGTAAAATGGCGACAAACAGT	4A	61,015	Solidity	TraesCS4A02G186500	l-Arabinose metabolic process	Alpha-l-arabinofuranosidase activity	–	0.000230482	29,34	Alpha-l-arabinofuranosidase 1-like (LOC123086376), transcript variant X2,
8		rs31569	TGCAGCGGATGGCTTCAACGTGCTGATGCCCAGCAACATAGACACAACCATAGCCGAGATCGGA	4A	61,015	Solidity	TraesCS4A02G187400	–	Protein binding	Nucleus	0.000143907	29,33	Protein HEAT STRESS TOLERANT DWD 1-like (LOC123086390)
9		rs52301	TGCAGGTACGAAACACCGAGGTGCTGCTGCTGCTGATGATGAACATTTTCGCTCCCAAAGGCCG	7A	0	ArBBox.2, Area.2, Breadth, Perim.2, MinR, Concavity, Solidity, Convexity	TraesCS7A02G015000	Autophagy, protein transport, COPII vesicle coating	–	Golgi membrane, endoplasmic reticulum	0.000228123	28	Protein transport protein SEC16A homolog (LOC123150363)
10		rs1418	TGCAGAACGAAAAACAGAGCATGTACTCAGTTTCTTATAATAAAAAGCTTCAAATATCATCAGA	4A	152,121	Concavity, Perim	TraesCS4A02G495100	Triterpenoid biosynthetic process	Lanosterol synthase activity, intramolecular transferase activity, beta-amyrin synthase activity	Lipid droplet	0.000313328 0.000179158	35	Cycloartenol Synthase-like (LOC123088646), transcript variant X3
1	Rain-fed	rs61378	TGCAGTGGCAGGAGAAATTCTAACGTTTTGTGGCGTGCGATAGCGAGACTGGCGGGAAAGTACC	1A	75,213	Frete, Perim, Chull, Perim.1, MaxR , MBCRadius	TraesCS1A02G361100	Protein-glycosylation	Glycosyltransferase-activity	Integral-component-of-membrane	0.000660236	27	Glucosamine inositolphosphorylceramide transferase 1
2		rs40522	TGCAGCTTACGTGCTCCACATTGGTACTCTTGCGCTGATAATCATGAACCGCAAACTCTATGCA	1B	45,574	MaxR, Frete, CHull	TraesCS1B02G176000	Cytokinin-metabolic-process	Catalytic-activity,-oxidoreductase-activity,-cytokinin-dehydrogenase-activity,-flavin-adenine-dinucleotide-binding,-FAD-binding	Extracellular-space	0.000485937	28	Cytokinin oxidase/dehydrogenase 8 (CKX8) gene
3		rs39898	TGCAGCTGGGGCTGTAGTGCCCCAGCGAACGCCCCTGGATGCGGCGAGTACCACGGCAGGGCCG	1B	45,574	MaxR, Chull, Frete, Perim	TraesCS1B02G178900	–	Kinase-activity	–	0.000335132	27,28	antifreeze protein Maxi-like (LOC123110907)
4		rs37640	TGCAGCTCGTCATCACCGCTCGCCCGCCCGCGTGGATGCAGAAGTGCTCGAACGCCGTGCGGAA	6A	55,893	TKW	TraesCS1B02G421100	Fatty-acid-biosynthetic-process	Acyltransferase-activity,acyltransferase-activity,-transferring-groups-other-than-aminoacyl-groups	Membrane	0.000503086	27,28,28	3-ketoacyl-CoA synthase 5-like (LOC123128295)
5		rs45754	TGCAGGATTTTTATTCAAGTTTGACGTACTTATTTAGCTATATATCCTGATGAATATGGGAAGT	2A	52,385	Aspect.ratio,Compactness,Concavity,ModRatio,Rfactor,Roundness,Shape,Sphericity	TraesCS2A02G115700		–	Membrane,integral-component-of-membrane	0.0000000503	27	Protein SRC2-like (LOC123184774)
6		rs51900	TGCAGGGTGGGGGCGGAGAAAAAGGAGGAGGGGCGGCCGAGATCGGAAGAGCGGGATCACCGA	2D	28,183	TKW	TraesCS2D02G082900	Vesiclemediated-transport	Protein-binding	Plasma-membrane,-integral-component-of-membrane	0.0000429131	29,27,28,29	Syntaxin-binding protein 5-like (LOC123043515)
7		rs16023	TGCAGCAGAGGTGGTTTGGAGGTTTGGTGGCGGCAGGATTCCCCTCCCGCGGGCGGCTCGGCTC	3B	56,892	TKW	TraesCS3B02G373500	Auxinactivated-signaling-pathwa,-transmembrane-transport,-intracellular-auxin-transport		Membrane,-integral-component-of-membrane	0.0000599241	27,29	Protein PIN-LIKES 6-like (LOC123071203)
8		rs1934	TGCAGAAGCAGTCCATCCCCACCAACCCAGCCAGCGCCGCCGCAACTACTCCTACGAGCGAAGC	3B	114,516	ArBBox,Area,ArEquivD,Carea,Circ,EquivEllAr,MBCRadius,PerEquivD	TraesCS3B02G581600	–	Metal-ion-binding	–	0.000605311	27	PH, RCC1 and FYVE domains-containing protein 1-like (LOC123072730)
9		rs15576	TGCAGCACTGGAAATTCTGGAGATGTGTAGGTCCAGACATAGTTTCTGTCGTCAATCAACTGTC	4A	149,842	Perim, Chull, MBCRadius, MaxR , Frete	TraesCS4A02G494000	–	Protein-binding	–	0.000454063	27	F-box protein At4g00755-like (LOC123083653), transcript variant X3
10		rs15577	TGCAGCACTGGAAATTCTGGAGATGTGTAGGTCCAGACATAGTTTCTGTCGTCAATTAACCGTC	4A	149,842	ArBBox,ArEquivD,Carea,Chull,EquivEllAr,Frete,MaxR,MBCRadius,Perim	TraesCS4A02G494000		Protein-binding	–	0.0000166	27,29	F-box protein At4g00755-like (LOC123083653), transcript variant X3

**Figure 7:**
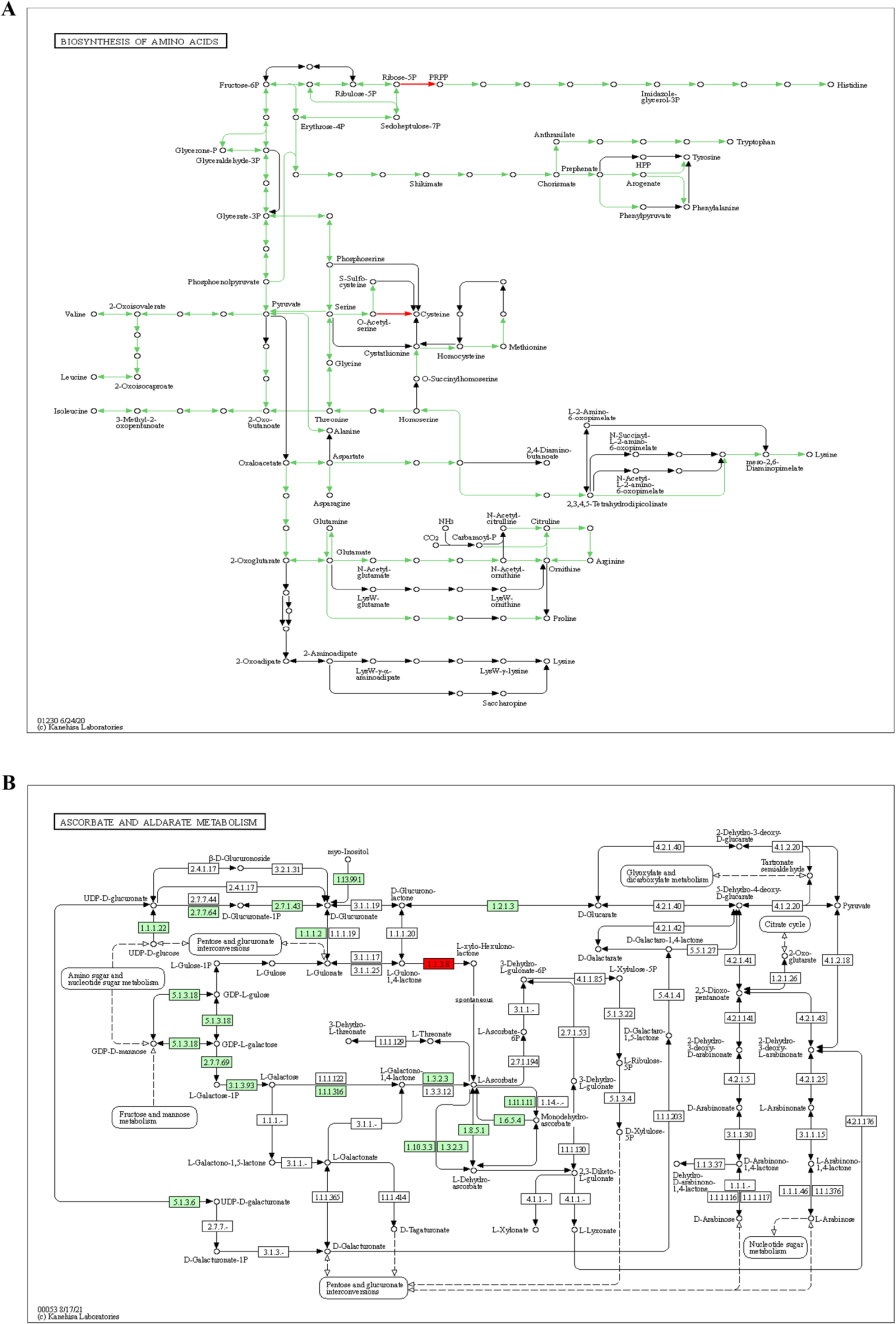
The KEGG pathway of biosynthesis of amino acids (**A**), the KEGG pathway of ascorbate and aldarate metabolism (**B**).

Based on blast gene IDs identified from the wheat reference genome, the following pathways were discovered: metabolic pathways (Supplementary Fig. 3), ubiquitin-mediated proteolysis (Supplementary Fig. 4), oxidative phosphorylation (Supplementary Fig. 5), carbon metabolism (Supplementary Fig. 6), biosynthesis of amino acids (Fig. 7a), pentose phosphate (Supplementary Fig. 7), ascorbate and aldarate metabolism (Fig. 7b), sulfur metabolism (Supplementary Fig. 8), and fatty acid elongation (Supplementary Fig. 9)^23–25^ (www.kegg.jp/kegg/kegg1.html).

In addition, using RNA-seq data from Rahimi et al.^26^ the DGEs belonging to the different transcription factor (TFs) families totaled 1,377. In this study, 443 genes encoding transcription factors were identified that showed differential expression between stress and normal treatments, Approximately the same number of TFs were identified among susceptible and tolerant genotypes (356 and 328 TFs, respectively). The difference between 9 and 18 days of water deficit was associated with 250 TFs. As genotype specific TFs, the majority of these TFs belong to the MYB, AP2/ERF-ERF related, MADS-M, B3, and, bHLH classes. There were, however, other TFs that were specific to long and short-term water deficits, including bZIP, C2H2, WRKY, NAC, and MYB. Furthermore, transcriptional regulators such as TAZ, TRAF, SNF2, and mTERF were identified. A summary of identified TFs among the different sets of DEGs in wheat is given in Supplementary Table 4.

## Discussion

A total of 298 Iranian wheat accessions including 208 landraces and 90 cultivars were assembled as a natural population for mapping QTLs related to seed traits using GWAS. A high level of variation found in wheat seed traits suggests the potential of GWAS for uncovering QTLs, as reported by Rahimi et al.^21^.

Most plant populations are structured because of artificial selection, isolation, or, nonrandom mating. As a result, genetic loci may be falsely related to traits when there is no authentic associations^15^. The possibility of false positives can increase in GWAS if population structure is not suitably accounted for, therefore evaluation of population structure is critical for any association mapping^36,37^. The panel of Iranian wheat accessions in this study was stratified into three groups. Cultivars made up one group, while landraces made up the other two groups, regardless of their geographic origins. Rahimi et al.^21^ observed the same groups on these Iranian wheat accessions. This mixture can be derived from grain exchanges between farmers in different local markets throughout the country^15^. As reported previously^21^, most Iranian cultivars originated from the International Maize and Wheat Improvement Center and only a small number of the cultivars derived from Iran, suggesting relatively narrow exploitation of native landraces in developing the new/old cultivars. Therefore, Iranian cultivars are suffered from a remarkable genetic bottleneck.

In accordance with previous reports, genome D indicated a low number of SNPs while most SNPs we located on the genomes B and A^21,38^. A similar situation was also uncovered for the number of marker pairs in LD, i.e., SNPs mapped to the genome B were about four times more common than those located on the genome D. The 3B and 2B chromosomes possess the most significant marker pairs, as reported previously^21^. The higher variation uncovered in the B and A genomes can be due to two reasons: (i) gene flow from *T. turgidum* as opposed to its absence from *Ae. tauschii* to *T. aestivum*; (ii) the output of older evolutionary history of the genomes B and A relative to genome D^39,40^. Furthermore, bottleneck impacts have likely happened owing to intense selection in native landraces during breeding schedules and this might lead to further impacts on genome D^15^. These impacts lead to a decrease in the effective population sizes, which in turn increase the loss of rare alleles in genomes B and A. A higher rate of low-frequency alleles in the D genome indicates a decrease in its allelic variant^41^. Of the observations in this study, most of the significant markers were present at a distance of less than 10 cM. Marker distances and LD throughout the genomes B and A were much lower than in the D genome. The higher level of linkage across three genomes in wheat cultivars reflects the impact of selection in the breeding history of those Cultivars. Population relatedness, mating systems, genetic drift, mutation, recombination, and selection are major forces affecting LD^42–44^. The fact that cultivars revealed higher LD in contrast to landraces, particularly in the genome D, is presumably a consequence of selection throughout the time of breeding efforts for key traits^45^.

A total of 10 and 10 MTAs by mrMLM, 3VmrMLM and MLM methods in the well-watered and rain-fed environments, respectively, were found within coding regions with *P*-value < 0.001. To remove any false-positive association, the most strongly markers were selected. Some MTAs discovered in this study are in line with previous reports.The GWAS identified 8 MTAs underlying seven putative QTL associated with grain perim on chromosomes 1A^27^, 1B^27,28^, 1D^29^, 2B^27^, 2D^29–32^, 3B^27,33^, and 7A^28^. Thus, MTA on Ch. 4A has not been reported and they are new for wheat seed perim. Six MTAs for area were found on Ch. 4A, 7A, 3B, 1D, 2D, and 3D. Earlier reports have detected MTAs/QTLs for area on Ch. 4A^27,29^, 7A^28^, 5B, 3B^27^, 1D^29^, 2D^30,31^. Therefore, MTAs on Ch. 3D are novel for area. Four MTAs for grain frete were recorded on Ch. 1A, 1B, 1D, and 4A in this study. Earlier research efforts have discovered MTAs/QTLs for frete on wheat Ch. 1A^28^, 1B^28^, 1D^29^, and 4A^27^. For seed breadth, two MTAs were revealed on Ch. 4A, and 7A. Previous research exhibited that this trait is linked with genomic regions on Ch. 2B, 4A^29,33,34^, 4B, 6A, and 7A^28^. The GWAS identified 4 MTAs underlying seven putative QTL associated with grain compactness and solidity on chromosomes 1A^27^, 2A^27^, 4A^29,33,34^, and 7A^28^. For instance, we detected QTLs on chromosomes 1A, 2A, 2B, 2D, 3A, 4A, 5A, 5B, and 7A for TKW under well-irrigated conditions. These observations agree with previously determined QTLs for TKW^46^. For rain-fed conditions, we also detected QTLs on chromosomes 1A, 1B, 2B, 2D, 3B, 4A, 5B, 6A, and 6B for TKW. These outputs are in agreement with the report by Ain et al.^47^ for TKW. Moreover, Gao et al.^48^ mapped a TKW QTL, namely QTKW.caas-7AL, in various conditions using an F8 population of Chinese spring wheat. Yan et al.^22^ revealed the *TaGW8* gene is associated with seed size in wheat by using GWAS. Breseghello and Sorrells^35^ revealed a QTL on chromosome 5B that affects seed length, with a moderate impact on seed size, under normal and stress conditions. They also reported QTLs for seed sphericity on 2D, 5B, QTLs for surface on1B, 2B, 4A, and QTLs for volume on 1B, 4A, 5B, 7B in wheat. Ma et al.^49^ located the *TaCYP78A3* gene, encoding cytochrome CYP78A3 P_450_, on the 7DS, 7BS, and 7AS, related to wheat seed shape and size. The authors demonstrated that silencing the *TaCYP78A3* gene could reduce the seed shape and size. Earlier reports have detected MTAs/QTLs for seed traits on Ch. 7D^50^, 7B^51^, 5B^52^, 3B^50^, 3A^52,53^, 2D^54^, 2B^50,51,54^, 2A^50^, and 1A^51–54^. Therefore, MTAs on Ch. 5A, 1B, 6B, and 1D are novel for seed traits.

In the recent study, the flanking sequences of imputed SNPs were identified and aligned versus the RefSeq v2.0^55^. The results indicated that most genes detected are responsible for key biosynthetic pathways. In a closer look, the proteins encoded by these genes are responsible for metal ion binding, peroxidase activity, ATP-binding, DNA-binding, protein kinase activity, enzyme inhibitor activity, etc. Such marker-trait associations have also been uncovered in previous reports^56,57^. These genes are found in genomic regions, which exhibit strong associations with key seed characteristics, suggesting that the genes can be regarded as favorable target genes for breeding efforts in future programs.

Analysis of RNA sequencing revealed genetic variations among genotypes as well as drought-responsive genes^26^. Our goal is to identify wheat genes that respond consistently to drought in dry, long-term conditions. Interestingly, we found a significantly higher number of genotype-specific DEGs in the susceptible genotype under normal and stress environments than in the tolerant genotypes, which is consistent with previous findings by Mia et al.^58^, and Fracasso et al.^59^ who both found similar expression pattern changes in susceptible materials.

From the gene network, several pathways were discovered in this study. Synthesis and elongation of fatty acids also are useful in response to drought in oats^60^. Protein phosphorylation contributes to a key role in wheat response to drought conditions^61^. Peptidase activity, DNA repair, DNA-binding transcription factor activity, and transmembrane transport were possibly responsible for drought tolerance^26^. Wheat avoids from oxidative stress and maintains cellular functions under drought by non-enzymatic antioxidants (ascorbate, etc.) and ROS scavenging enzymes (SOD, CAT, etc.)^62^. The role of ubiquitination in metabolic pathways of tea in response to drought has also been proved by Xie et al.^63^. Such essential roles for the biosynthesis of secondary metabolites have also been reported^64^. A metabolic pathway that is associated with drought stress tolerance involves genes such as ABA-responsive element-binding factor, sucrose synthase, and sucrose-phosphate synthase in the metabolism of ascorbate and aldarate^65^. Drought stimulates energy-intensive processes such as osmolyte production and oxidative phosphorylation, as well as increases respiratory rates^66^. Proline is an amino acid produced by the amino acid pathway. Proline has been linked to a number of osmoprotective properties, such as the ability to regulate humidity and activate genes that produce antioxidizing enzymes that scavenge reactive oxygen species (ROS)^67,68^. In drought-stressed genotypes, proline levels increased faster and by a greater proportion than those of their sensitive counterparts, emphasizing its importance for drought tolerance breeding. Proline-controlling genes have cumulative effects on proline content^69,70^. These findings are similar to the previous report^21^. Oxidative damage is induced by the production of the reactive oxygen species (ROSs), including OH^–^, O_2_^–^, and H_2_O_2_^67^. These ROSs in high concentrations are detrimental and degrade photosynthetic pigments, proteins, etc. In the context of osmotic tolerance, crops generate proline osmolyte to adjust water status^69,70^. Crops also adopt tissue tolerance by using the scavenging system to alleviate ROSs effects. The first enzyme committed to remove ROSs is the superoxide dismutase (SOD), which can dismutate O_2_^–^ to H_2_O_2_. H_2_O_2_, in turn, is catalyzed by peroxidase (POD) and catalase (CAT) to O_2_ and H_2_O ^67,68^. Expressed wheat-originated CAT and SOD in Arabidopsis can enhance tolerance to multiple abiotic stimuli, such as high-drought conditions^62^. APX, GPX, and PPO enzymes are other key components of non-enzymatic scavenging systems in crops^68^.

## Conclusion

Of the current findings, new QTLs were uncovered in the panel of Iranian wheat landraces in multi-environment phenotypic data, i.e., rain-fed (drought) and well-watered (normal). Data from multi-environment, multi-year phenotypic experiments could reveal QTL that are stable across environments. Major QTLs controlling seed traits were uncovered on the genome B (1B and 3B) and chromosomes 1A and 4A. QTL for grain shape traits were identified in chromosome regions in which major QTL or/and genes were detected in previous studies. Using digital image analysis is a non-invasive and inexpensive alternative to trait evaluations.

## Methods

### Plant materials and experimental conditions

A total of 208 wheat landraces and 90 cultivars (Supplementary Table 5) were analyzed in an alpha-lattice experiment with two repeats during two crop seasons (2018–2019 and 2019–2020) under rain-fed (drought) and well-watered (normal) conditions. In the field, the plots consisted of four rows (1*1 m^2^) at 0.5 m intervals. The irrigation threshold in the well-watered crops was considered according to 40 mm evaporation from an evaporation pan. The crop coefficient [*K*_C_] and reference crop evapotranspiration [ET_0_ = E_pan_ × K_pan_; where *E*_pan_ is the evaporation depth from the pan surface (40 mm) and *K*_pan_ is a pan coefficient (0.8) for each month] were utilized to measure evapotranspiration (ET_C_ = K_C_ × ET_0_). The irrigation time was determined according to the ratio of the assigned water for 1400 m^2^ (the cultivation area of 298 genotypes in two repeats) to water discharge (10.8 m^3^/h). The volume of water needed for each hectare (m^3^/ha) was determined by the depth of ET_0_ (mm) multiplied by 10. The wheat cultivated under the rain-fed regime was only exposed to rainfall, the only available water source. The pattern of monthly rainfall for the cropping seasons is presented in Table 4. The authors declare that all study complies with relevant institutional, national, and international guidelines and legislation for plant ethics in the “Methods” section. Samples are provided from the Gene Bank of Agronomy and Plant Breeding Group and these samples are available at USDA with the USDA PI number (Supplementary Table 5). The authors declare that all that permissions or licenses were obtained to collect the wheat plant.

**Table 4:** Climatic data in the studied environments and pattern of monthly precipitation and irrigation for the 2018–2019 and 2019–2020 cropping seasons.

Year	Month	Max Temperature °C	Min Temperature °C	Average Temperature °C	Average rainfall, mm	Average relative humidity	Sunny hours	Evaporation, mm
2018–2019	November	14.561	4.104	10.900	0.031	45.810	6.893	3.068
	December	9.242	− 0.119	4.671	1.326	60.134	5.065	0.000
	January	8.406	− 0.613	3.668	0.485	57.750	6.652	0.000
	February	7.871	− 2.254	2.536	0.965	61.429	6.868	0.000
	March	14.216	4.623	9.271	1.240	56.847	5.942	0.179
	April	21.093	9.563	15.110	1.555	49.954	6.587	4.497
	May	29.229	14.261	21.935	0.710	38.722	10.435	7.377
	June	34.159	17.597	26.083	0.000	32.304	12.763	11.676
2019–2020	November	17.080	6.383	11.520	0.021	43.479	6.960	3.189
	December	12.303	1.652	6.671	0.152	50.419	7.226	0.000
	January	9.077	− 0.055	4.052	0.640	54.476	6.526	0.000
	February	10.739	2.039	6.464	1.094	64.755	5.829	0.000
	March	20.558	8.377	14.652	0.455	38.952	7.303	0.000
	April	19.983	7.793	13.633	1.527	51.413	7.563	6.714
	May	25.513	12.061	18.432	1.841	54.907	8.287	6.161
	June	33.807	17.347	25.583	0.241	37.492	11.100	11.143

	Month	ET_0_ (mm)	K_C_	ET_C_ (mm)	Required water per ha (m^3^/ha)	Required water for 1377 m^2^ (m^3^)	Water discharge (m^3^/h)	Period of irrigation (h)
2019 and 2020	March	40	0.92	36.8	368	50.69	10.8	4.69
	April	40	1.33	53.2	532	73.28	10.8	6.79
	May	40	1.15	46	46	63.37	10.8	5.87
	June	40	0.58	23.2	232	31.96	10.8	2.96

### Digital image analysis

**Figure 8:**
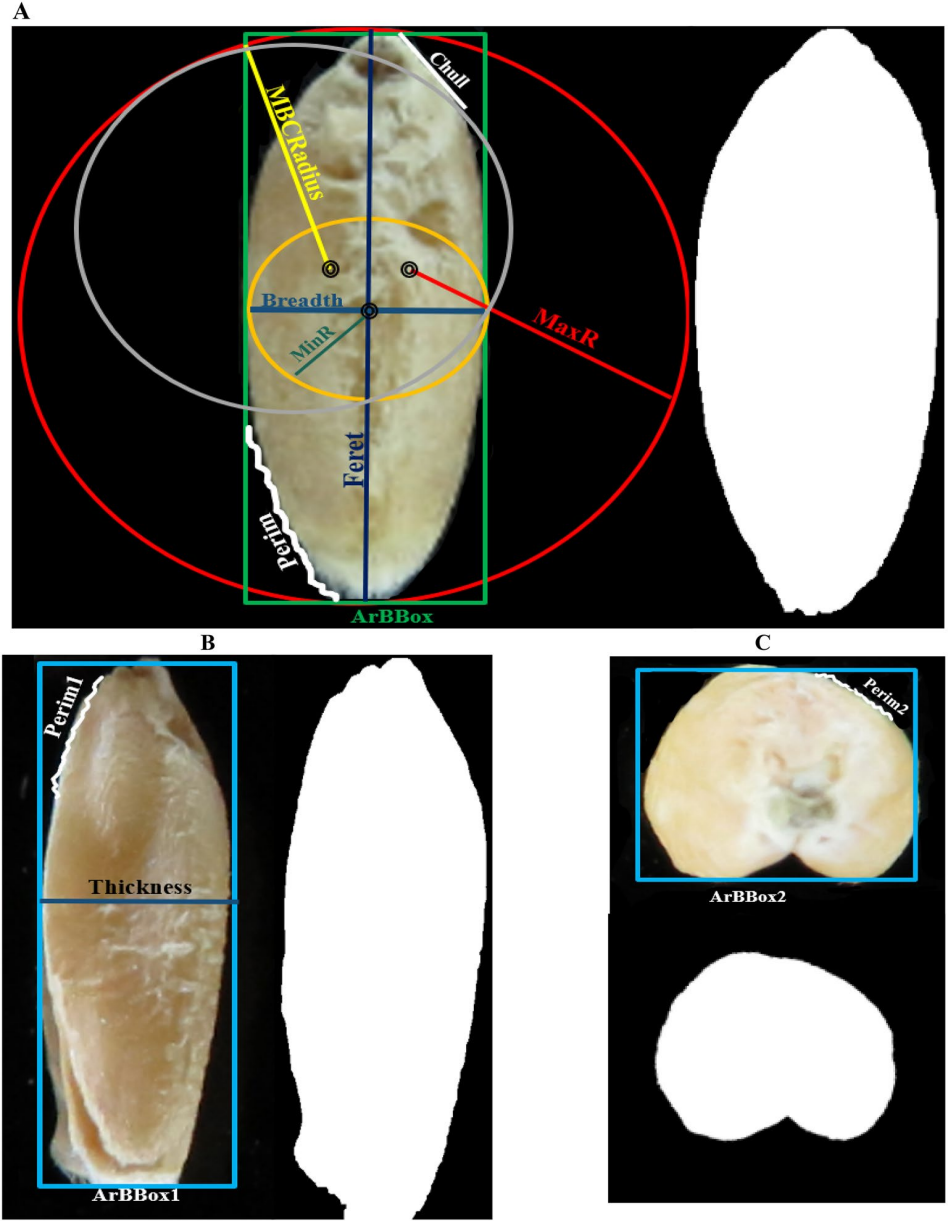
Graphical presentations of morphometric traits measured on wheat seeds (Refer to Table 5). (**A**) dorsal, (**B**) lateral, (**C**) vertical.

The digital images of wheat seeds were provided by a camera (Canon SX540 HS) equipped with 800 dpi resolution. After imaging, the pictures were analyzed and processed via the Python 3.7 software^6,71,72^ to evaluate a total of 34 morphometric variables in bread wheat seeds (Table 5; Fig. 8).

**Table 5:** Morphometric traits measured on wheat seeds.

Seed mode	Parameter (unit)	Description
Dorsal	Perim (mm)	Perimeter, calculated from the centers of the boundary pixel
	Area (mm^2^)	Area inside the polygon defined by the perimeter
	MinR (mm)	Radius of the inscribed circle centered at the middle of mass
	MaxR (mm)	Radius of the enclosing circle centered at the middle of mass
	Feret (mm)	Largest axis length
	Breadth (mm)	Largest axis perpendicular to the Feret
	Chull (mm)	Convex hull or convex polygon calculated from pixel centers
	CArea (mm^2^)	Area of the convex hull polygon
	MBCRadius (mm)	Radius of the minimal bounding circle
	AspRatio	Aspect ratio = Feret/Breadth
	Circ	Circularity = 4·π·Area/Perimeter^2^
	Roundness	Roundness = 4·Area/(π·Feret^2^ )
	ArEquivD	Area equivalent diameter = √((4/π)·Area)
	PerEquivD	Perimeter equivalent diameter = Area/π
	EquivEllAr	Equivalent ellipse area = (π·Feret·Breadth)/4
	Compactness	Compactness = √((4/π)·Area)/Feret
	Solidity	Solidity = Area/Convex_Area
	Concavity	Concavity = Convex_Area-Area
	Convexity	Convexity = Convex_hull/Perimeter
	Shape	Shape = Perimeter^2^/Area
	RFactor	RFactor = Convex_Hull/(Feret·π)
	ModRatio	Modification ratio = (2·MinR)/Feret
	Sphericity	phericity = MinR/MaxR
	ArBBox (mm^2^)	Area of the bounding box along the feret diameter = Feret·Breadth
	Rectang	Rectangularity = Area/ArBBox
Lateral	Perim.1 (mm)	Perimeter, calculated from the centers of the boundary pixel
	Area.1 (mm^2^)	Area inside the polygon defined by the perimeter
	ArBBox.1 (mm^2^)	Area of the bounding box along the feret diameter = Feret· Thickness
	Thickness (mm)	Largest axis perpendicular to the Feret
Vertical	Perim.2 (mm)	Perimeter, calculated from the centers of the boundary pixel
	Area.2 (mm^2^)	Area inside the polygon defined by the perimeter
	ArBBox.2 (mm^2^)	Area of the bounding box along the feret diameter = Breadth · Thickness
	Volume (mm^3^)	Volume = (4/3) π (Feret/2)(Breadth/2)( Thickness/2)
	TKW (gr)	The weight of one thousand seeds

### GBS and imputation

The establishment and sequencing of the sequence library for the wheat accessions were carried out following the procedure as elucidated by Alipour et al.^73^. After trimming reads to 64 bp and categorizing them into tags, single-nucleotide polymorphisms (SNPs) were discovered via internal alignments, which permitting for mismatch up to 3 bp. The pipeline UNEAKGBS was utilized for SNP calling, where SNPs with low minor allele frequency < 1% and reads with a low-quality score (< 15) were discarded to keep away from false-positive markers, which are derived from errors in the sequencing process. The imputation was performed according to available allele frequency calculated after accounting for the haplotype phase^74^ in BEAGLE version 3.3.2. The reference genome W7984 was specified that harboring the highest imputation accuracy^75^ among four various reference genomes during imputation. The linkage disequilibrium decay of various chromosomes was obtained based on LOESS regression and RStudio, the ggplot2 package^76^.

### Population structure and Kinship matrix

Population structure was assayed in the Iranian wheat landraces and cultivars through STRUCTURE version 2.3.4^37^. A simulation phase consisted of 10,000 steps for K = 1 up to 10 along with an admixture model was used in this study. ΔK was utilized to estimate the most likely number of subpopulations in this study. To measure LD among markers, the expected and observed allele frequencies were exerted in TASSEL version 5^77^. Q-matrix was used as a structural matrix for the association study. A neighbor-joining tree was formed according to a pairwise distance matrix counted in TASSEL^77^ and visualized using Archaeopteryx to explore the relationships between the Iranian wheat landraces and cultivars.

### Genome-wide association study

MLM^78^, mrMLM^79,80^ and 3VmrMLM^81^ approaches were used to estimate the marker effect. IIIVmrMLM^82^ was used to identify QTN and QEI in this study. The first approach led to the most accurate marker-trait association. The K, Q, and Q + K versions of the MLM approach were utilized to modulate both effects of more diffused relationships (K) among accessions and population structure (Q) via TASSEL. The association mapping for the MLM, mrMLM, and 3VmrMLM models was performed using the package GAPIT and IIIVmrMLM in Rstudio. In the MLM approach, accessions are regarded as a random effect and the relevance among them was transferred by a kinship matrix. The elements in this matrix were utilized as similarities and the resultant clusters were visualized using a UPGMA-based heatmap via the GAPIT package. A Manhattan plot was derived from a comparison scenario using the package GAPIT to explore the association between genotype and phenotype, SNPs were ordered according to their base-pair positions and chromosomes. In the Manhattan plot, the y-axis represented the negative logarithm of *P*-value derived from the F-test and the x-axis represented the SNP genomic position.

### Annotation of genes

Sequences around all significantly associated SNPs were provided from the 90 K SNP database of wheat. These sequences were utilized for the gene annotation via aligning to the IWGSC RefSeq V2.0 (URGI-INRA) using the database gramene (http://www.gramene.org/). The functions of putative genes were discovered via evaluating the pathways including the encoded enzymes. After aligning SNPs sequences to the reference, overlapped genes with the largest identity percentages and blast scores were picked out for further analysis. The ensemble-gramene database was used to extract the molecular functions and biological processes of genes in the gene ontology. Moreover, the sequences of significant SNPs were utilized in the enrichment analysis of gene ontology via KOBAS version 2.0 to test for statistically enriched pathways in the database KEGG (https://www.genome.jp/kegg/; www.kegg.jp/kegg/kegg1.html).

### Identification of candidate genes via BLASTn

Identification of gene IDs was based on sequences of genes associated with seed traits (https://plants.ensembl.org/index.html). An analysis of whole CDS sequences of candidate genes was conducted using BLASTn analysis (nucleotide Basic Local Alignment Search Tool) from the National Center for Biotechnology Information (NCBI; https://www.ncbi.nlm.nih.gov/). In this alignment, default settings are used to align these sequences to *Triticum aestivume* species. An ideal match or high similarity was determined when all queries were covered, the Expect (E) value was zero, and the identity was greater than 99%. In addition, RNA-seq data from Rahimi et al., which were based on the tolerant and sensitive genotypes selected from this field experiment, were used to identify the genes involved in the drought stress path.

### Statistical analysis

The descriptive statistics, variance analysis (ANOVA) and correlation of Seed imaging data were performed via SAS version 9.4 and RStudio separately for the two conditions, rain-fed (drought) and well-watered (normal). For advanced linear analysis, the adjusted means were derived from an alpha-lattice experiment using GLM and MLM models. Correlation and box-plot analysis were carried out in RStudio using the corrplot and ggpubr packages to assay the relationship and distribution of wheat seed morphometric traits.

### Permission for land study

The authors declare that all land experiments and studies were carried out according to authorized rules.

## Supplementary Information

Supplementary Information.

## Data Availability

The datasets generated and analyzed during the current study are available in the *Figshare* repository [10.6084/m9.figshare.18774476.v1].
