# Supplementary material for: Genome-wide association mapping for wheat morphometric seed traits in Iranian landraces and cultivars under rain-fed and well-watered conditions
Source: Sci Rep. 2022 Oct 25;12:17839. doi: 10.1038/s41598-022-22607-0 (PMC9596696; doi:10.1038/s41598-022-22607-0)
Supplement: Supplementary file 1 — Supplementary Information. [file 41598_2022_22607_MOESM1_ESM.docx]

**Supplementary Table 1.** Correlation coefficients between the morphometric seed traits for Iranian landraces and cultivars in the well-watered (above diagonal) and rain-fed (below diagonal) environments.

|  | MaxR | MinR | MBCRadius | CHull | CArea | Area | Perim | Frete | Breadth | ArBBox | Aspect ratio | Circ | Roundness | ArEquivD | PerEquivD | EquivEllAr | Compactness | Solidity | Concavity | Convexity | Shape | RFactor | ModRatio | Sphericity | Rectang | Perim.1 | Area.1 | ArBBox.1 | Thickness | Perim.2 | Area.2 | ArBBox.2 | Volume | TKW |
| --- | --- | --- | --- | --- | --- | --- | --- | --- | --- | --- | --- | --- | --- | --- | --- | --- | --- | --- | --- | --- | --- | --- | --- | --- | --- | --- | --- | --- | --- | --- | --- | --- | --- | --- |
| MaxR | 1.00 | 0.30 | 0.94 | 0.97 | 0.83 | 0.82 | 0.97 | 1.00 | 0.30 | 0.83 | 0.66 | 0.82 | 0.63 | 0.83 | 0.82 | 0.83 | -0.68 | -0.08 | 0.56 | -0.04 | 0.58 | -0.71 | -0.69 | 0.66 | -0.20 | 0.90 | 0.77 | 0.80 | 0.41 | 0.38 | 0.39 | 0.38 | 0.73 | 0.61 |
| MinR | 0.20 | 1.00 | 0.60 | 0.50 | 0.77 | 0.78 | 0.50 | 0.28 | 1.00 | 0.77 | -0.52 | 0.78 | -0.53 | 0.77 | 0.78 | 0.78 | 0.48 | 0.39 | 0.09 | -0.03 | -0.49 | 0.42 | 0.48 | -0.52 | -0.08 | 0.36 | 0.53 | 0.49 | 0.65 | 0.89 | 0.88 | 0.89 | 0.79 | 0.73 |
| MBCRadius | 0.94 | 0.53 | 1.00 | 0.99 | 0.97 | 0.96 | 0.98 | 0.94 | 0.60 | 0.97 | 0.37 | 0.96 | 0.35 | 0.96 | 0.96 | 0.97 | -0.40 | 0.07 | 0.50 | -0.04 | 0.32 | -0.45 | -0.41 | 0.37 | -0.19 | 0.88 | 0.83 | 0.84 | 0.57 | 0.63 | 0.64 | 0.63 | 0.89 | 0.76 |
| CHull | 0.98 | 0.38 | 0.98 | 1.00 | 0.93 | 0.93 | 0.99 | 0.97 | 0.50 | 0.93 | 0.48 | 0.93 | 0.46 | 0.93 | 0.93 | 0.93 | -0.50 | 0.02 | 0.53 | -0.04 | 0.41 | -0.53 | -0.51 | 0.48 | -0.15 | 0.90 | 0.83 | 0.84 | 0.53 | 0.56 | 0.57 | 0.55 | 0.84 | 0.73 |
| CArea | 0.79 | 0.74 | 0.95 | 0.89 | 1.00 | 1.00 | 0.93 | 0.82 | 0.77 | 1.00 | 0.13 | 1.00 | 0.12 | 1.00 | 1.00 | 1.00 | -0.16 | 0.18 | 0.43 | -0.04 | 0.09 | -0.21 | -0.18 | 0.14 | -0.10 | 0.81 | 0.83 | 0.82 | 0.66 | 0.77 | 0.77 | 0.77 | 0.94 | 0.83 |
| Area | 0.77 | 0.76 | 0.94 | 0.88 | 1.00 | 1.00 | 0.92 | 0.81 | 0.78 | 0.99 | 0.12 | 1.00 | 0.11 | 1.00 | 1.00 | 0.99 | -0.15 | 0.21 | 0.40 | -0.01 | 0.07 | -0.20 | -0.16 | 0.12 | -0.08 | 0.80 | 0.83 | 0.82 | 0.66 | 0.78 | 0.78 | 0.77 | 0.94 | 0.83 |
| Perim | 0.98 | 0.39 | 0.98 | 1.00 | 0.89 | 0.88 | 1.00 | 0.96 | 0.51 | 0.93 | 0.47 | 0.92 | 0.44 | 0.92 | 0.92 | 0.93 | -0.50 | -0.07 | 0.61 | -0.15 | 0.45 | -0.53 | -0.50 | 0.48 | -0.20 | 0.90 | 0.82 | 0.84 | 0.53 | 0.56 | 0.57 | 0.55 | 0.84 | 0.72 |
| Frete | 1.00 | 0.19 | 0.93 | 0.98 | 0.78 | 0.76 | 0.97 | 1.00 | 0.28 | 0.82 | 0.67 | 0.81 | 0.65 | 0.82 | 0.81 | 0.82 | -0.69 | -0.08 | 0.56 | -0.03 | 0.59 | -0.72 | -0.70 | 0.67 | -0.19 | 0.90 | 0.77 | 0.79 | 0.40 | 0.37 | 0.38 | 0.37 | 0.72 | 0.60 |
| Breadth | 0.21 | 1.00 | 0.54 | 0.39 | 0.75 | 0.77 | 0.39 | 0.19 | 1.00 | 0.78 | -0.52 | 0.78 | -0.53 | 0.77 | 0.78 | 0.78 | 0.48 | 0.35 | 0.14 | -0.08 | -0.47 | 0.42 | 0.48 | -0.51 | -0.11 | 0.37 | 0.53 | 0.49 | 0.65 | 0.89 | 0.88 | 0.89 | 0.80 | 0.73 |
| ArBBox | 0.77 | 0.78 | 0.94 | 0.87 | 0.99 | 0.99 | 0.87 | 0.76 | 0.79 | 1.00 | 0.13 | 0.99 | 0.10 | 0.99 | 0.99 | 1.00 | -0.17 | 0.16 | 0.44 | -0.07 | 0.10 | -0.22 | -0.17 | 0.13 | -0.19 | 0.80 | 0.82 | 0.81 | 0.65 | 0.78 | 0.78 | 0.77 | 0.94 | 0.82 |
| Aspect ratio | 0.62 | -0.63 | 0.31 | 0.47 | 0.03 | 0.00 | 0.46 | 0.64 | -0.63 | -0.02 | 1.00 | 0.12 | 0.99 | 0.13 | 0.12 | 0.13 | -0.99 | -0.36 | 0.40 | 0.03 | 0.90 | -0.97 | -0.99 | 1.00 | -0.09 | 0.52 | 0.27 | 0.32 | -0.15 | -0.36 | -0.35 | -0.36 | 0.02 | -0.03 |
| Circ | 0.77 | 0.76 | 0.94 | 0.88 | 1.00 | 1.00 | 0.88 | 0.76 | 0.77 | 0.99 | 0.00 | 1.00 | 0.11 | 1.00 | 1.00 | 0.99 | -0.15 | 0.21 | 0.40 | -0.01 | 0.07 | -0.20 | -0.16 | 0.12 | -0.08 | 0.80 | 0.83 | 0.82 | 0.66 | 0.78 | 0.78 | 0.77 | 0.94 | 0.83 |
| Roundness | 0.58 | -0.65 | 0.27 | 0.43 | 0.00 | -0.02 | 0.42 | 0.59 | -0.65 | -0.06 | 0.99 | -0.02 | 1.00 | 0.12 | 0.11 | 0.10 | -0.96 | -0.29 | 0.33 | 0.10 | 0.85 | -0.93 | -0.98 | 0.98 | 0.04 | 0.50 | 0.27 | 0.31 | -0.15 | -0.38 | -0.36 | -0.37 | 0.00 | -0.03 |
| ArEquivD | 0.78 | 0.76 | 0.95 | 0.89 | 1.00 | 1.00 | 0.89 | 0.77 | 0.76 | 0.99 | 0.01 | 1.00 | -0.01 | 1.00 | 1.00 | 0.99 | -0.15 | 0.20 | 0.40 | -0.01 | 0.08 | -0.21 | -0.17 | 0.13 | -0.09 | 0.81 | 0.83 | 0.82 | 0.65 | 0.77 | 0.77 | 0.77 | 0.94 | 0.83 |
| PerEquivD | 0.77 | 0.76 | 0.94 | 0.88 | 1.00 | 1.00 | 0.88 | 0.76 | 0.77 | 0.99 | 0.00 | 1.00 | -0.02 | 1.00 | 1.00 | 0.99 | -0.15 | 0.21 | 0.40 | -0.01 | 0.07 | -0.20 | -0.16 | 0.12 | -0.08 | 0.80 | 0.83 | 0.82 | 0.66 | 0.78 | 0.78 | 0.77 | 0.94 | 0.83 |
| EquivEllAr | 0.77 | 0.78 | 0.94 | 0.87 | 0.99 | 0.99 | 0.87 | 0.76 | 0.79 | 1.00 | -0.02 | 0.99 | -0.06 | 0.99 | 0.99 | 1.00 | -0.17 | 0.16 | 0.44 | -0.07 | 0.10 | -0.22 | -0.17 | 0.13 | -0.19 | 0.80 | 0.82 | 0.81 | 0.65 | 0.78 | 0.78 | 0.77 | 0.95 | 0.82 |
| Compactness | -0.66 | 0.59 | -0.36 | -0.50 | -0.06 | -0.03 | -0.49 | -0.67 | 0.58 | -0.04 | -0.98 | -0.03 | -0.94 | -0.05 | -0.03 | -0.04 | 1.00 | 0.41 | -0.46 | 0.05 | -0.92 | 0.99 | 0.99 | -0.99 | 0.23 | -0.53 | -0.29 | -0.34 | 0.12 | 0.33 | 0.31 | 0.33 | -0.06 | 0.01 |
| Solidity | -0.39 | 0.45 | -0.18 | -0.28 | 0.02 | 0.06 | -0.31 | -0.39 | 0.43 | 0.04 | -0.65 | 0.06 | -0.60 | 0.05 | 0.06 | 0.04 | 0.68 | 1.00 | -0.81 | 0.80 | -0.69 | 0.34 | 0.36 | -0.39 | 0.48 | -0.04 | 0.09 | 0.04 | 0.15 | 0.27 | 0.25 | 0.27 | 0.17 | 0.22 |
| Concavity | 0.75 | 0.05 | 0.67 | 0.72 | 0.54 | 0.50 | 0.75 | 0.75 | 0.07 | 0.52 | 0.54 | 0.50 | 0.49 | 0.51 | 0.50 | 0.52 | -0.59 | -0.81 | 1.00 | -0.77 | 0.69 | -0.43 | -0.43 | 0.43 | -0.50 | 0.51 | 0.40 | 0.45 | 0.25 | 0.21 | 0.22 | 0.20 | 0.40 | 0.28 |
| Convexity | -0.35 | -0.21 | -0.37 | -0.35 | -0.34 | -0.32 | -0.40 | -0.34 | -0.24 | -0.37 | -0.09 | -0.32 | -0.01 | -0.33 | -0.32 | -0.37 | 0.18 | 0.53 | -0.63 | 1.00 | -0.39 | 0.01 | 0.01 | -0.01 | 0.53 | -0.04 | -0.01 | -0.04 | -0.06 | -0.08 | -0.08 | -0.07 | -0.07 | -0.02 |
| Shape | 0.64 | -0.59 | 0.35 | 0.49 | 0.06 | 0.02 | 0.49 | 0.65 | -0.57 | 0.02 | 0.97 | 0.02 | 0.94 | 0.04 | 0.02 | 0.03 | -0.98 | -0.77 | 0.65 | -0.26 | 1.00 | -0.87 | -0.90 | 0.92 | -0.33 | 0.45 | 0.21 | 0.27 | -0.14 | -0.33 | -0.31 | -0.33 | 0.01 | -0.06 |
| RFactor | -0.69 | 0.51 | -0.41 | -0.53 | -0.12 | -0.10 | -0.53 | -0.70 | 0.50 | -0.11 | -0.94 | -0.10 | -0.89 | -0.11 | -0.10 | -0.11 | 0.98 | 0.61 | -0.56 | 0.21 | -0.93 | 1.00 | 0.98 | -0.96 | 0.26 | -0.56 | -0.32 | -0.38 | 0.09 | 0.28 | 0.26 | 0.28 | -0.11 | -0.03 |
| ModRatio | -0.65 | 0.61 | -0.34 | -0.49 | -0.06 | -0.04 | -0.49 | -0.66 | 0.60 | -0.02 | -0.99 | -0.04 | -0.97 | -0.05 | -0.04 | -0.02 | 0.99 | 0.65 | -0.56 | 0.13 | -0.96 | 0.97 | 1.00 | -0.99 | 0.12 | -0.55 | -0.31 | -0.36 | 0.10 | 0.32 | 0.30 | 0.32 | -0.07 | 0.00 |
| Sphericity | 0.63 | -0.63 | 0.32 | 0.47 | 0.04 | 0.01 | 0.47 | 0.64 | -0.62 | -0.01 | 1.00 | 0.01 | 0.98 | 0.02 | 0.01 | -0.01 | -0.98 | -0.67 | 0.56 | -0.11 | 0.98 | -0.94 | -0.99 | 1.00 | -0.12 | 0.52 | 0.27 | 0.32 | -0.15 | -0.36 | -0.34 | -0.36 | 0.02 | -0.03 |
| Rectang | -0.13 | -0.27 | -0.21 | -0.11 | -0.15 | -0.14 | -0.14 | -0.12 | -0.30 | -0.27 | 0.14 | -0.14 | 0.29 | -0.15 | -0.14 | -0.27 | 0.04 | 0.18 | -0.22 | 0.46 | -0.02 | 0.13 | -0.09 | 0.11 | 1.00 | -0.11 | -0.02 | -0.09 | -0.03 | -0.08 | -0.08 | -0.07 | -0.14 | -0.01 |
| Perim.1 | 0.88 | 0.30 | 0.87 | 0.89 | 0.77 | 0.76 | 0.89 | 0.88 | 0.31 | 0.75 | 0.46 | 0.76 | 0.42 | 0.77 | 0.76 | 0.76 | -0.49 | -0.27 | 0.63 | -0.31 | 0.48 | -0.52 | -0.48 | 0.47 | -0.13 | 1.00 | 0.93 | 0.95 | 0.64 | 0.54 | 0.55 | 0.54 | 0.80 | 0.68 |
| Area.1 | 0.76 | 0.56 | 0.85 | 0.83 | 0.86 | 0.86 | 0.82 | 0.75 | 0.56 | 0.84 | 0.15 | 0.86 | 0.13 | 0.86 | 0.86 | 0.85 | -0.18 | 0.01 | 0.46 | -0.23 | 0.16 | -0.22 | -0.18 | 0.16 | -0.10 | 0.91 | 1.00 | 0.99 | 0.84 | 0.73 | 0.75 | 0.74 | 0.90 | 0.78 |
| ArBBox.1 | 0.78 | 0.54 | 0.86 | 0.84 | 0.85 | 0.85 | 0.84 | 0.77 | 0.55 | 0.85 | 0.18 | 0.85 | 0.15 | 0.85 | 0.85 | 0.85 | -0.22 | -0.05 | 0.51 | -0.29 | 0.20 | -0.27 | -0.21 | 0.19 | -0.16 | 0.93 | 0.99 | 1.00 | 0.84 | 0.71 | 0.73 | 0.72 | 0.90 | 0.76 |
| Thickness | 0.40 | 0.75 | 0.61 | 0.53 | 0.73 | 0.74 | 0.53 | 0.39 | 0.76 | 0.74 | -0.28 | 0.74 | -0.30 | 0.74 | 0.74 | 0.75 | 0.25 | 0.25 | 0.21 | -0.19 | -0.26 | 0.20 | 0.25 | -0.27 | -0.14 | 0.60 | 0.85 | 0.84 | 1.00 | 0.88 | 0.90 | 0.90 | 0.86 | 0.73 |
| Perim.2 | 0.33 | 0.92 | 0.61 | 0.49 | 0.78 | 0.80 | 0.49 | 0.32 | 0.92 | 0.81 | -0.47 | 0.80 | -0.49 | 0.79 | 0.80 | 0.81 | 0.43 | 0.35 | 0.15 | -0.22 | -0.43 | 0.36 | 0.44 | -0.46 | -0.24 | 0.48 | 0.74 | 0.73 | 0.92 | 1.00 | 0.98 | 0.96 | 0.90 | 0.79 |
| Area.2 | 0.32 | 0.92 | 0.60 | 0.48 | 0.78 | 0.80 | 0.48 | 0.30 | 0.92 | 0.80 | -0.48 | 0.80 | -0.50 | 0.79 | 0.80 | 0.80 | 0.44 | 0.37 | 0.13 | -0.21 | -0.44 | 0.38 | 0.45 | -0.47 | -0.21 | 0.47 | 0.74 | 0.73 | 0.92 | 0.98 | 1.00 | 0.96 | 0.90 | 0.79 |
| ArBBox.2 | 0.31 | 0.92 | 0.60 | 0.47 | 0.77 | 0.79 | 0.48 | 0.30 | 0.93 | 0.80 | -0.49 | 0.79 | -0.51 | 0.78 | 0.79 | 0.80 | 0.45 | 0.35 | 0.15 | -0.23 | -0.44 | 0.38 | 0.46 | -0.47 | -0.23 | 0.47 | 0.74 | 0.73 | 0.92 | 0.96 | 0.96 | 1.00 | 0.90 | 0.78 |
| Volume | 0.65 | 0.82 | 0.86 | 0.77 | 0.95 | 0.95 | 0.78 | 0.64 | 0.83 | 0.96 | -0.14 | 0.95 | -0.17 | 0.95 | 0.95 | 0.96 | 0.10 | 0.13 | 0.42 | -0.31 | -0.10 | 0.03 | 0.11 | -0.13 | -0.22 | 0.73 | 0.90 | 0.89 | 0.90 | 0.91 | 0.91 | 0.91 | 1.00 | 0.85 |
| TKW | 0.42 | 0.69 | 0.61 | 0.53 | 0.72 | 0.73 | 0.53 | 0.41 | 0.70 | 0.72 | -0.22 | 0.73 | -0.21 | 0.73 | 0.73 | 0.72 | 0.21 | 0.35 | 0.11 | -0.09 | -0.23 | 0.15 | 0.20 | -0.21 | -0.05 | 0.50 | 0.68 | 0.66 | 0.71 | 0.74 | 0.76 | 0.73 | 0.76 | 1.00 |

**Supplementary Table 2**. A summary of LD observed among marker pairs and the number of significant marker pairs per genome and chromosome using imputed SNPs.

| Chromosome | Total | | | | Landrace | | | | Cultivar | | | |
| --- | --- | --- | --- | --- | --- | --- | --- | --- | --- | --- | --- | --- |
|  | TNSP | r^2^ | Distance (cM) | NSSP | TNSP | r^2^ | Distance (cM) | NSSP | TNSP | r^2^ | Distance (cM) | NSSP |
| 1A | 111575 | 0.111829 | 1.333712 | 49917 (44.74%) | 94575 | 0.116906 | 1.568634 | 34895 (36.9%) | 85625 | 0.148069 | 1.736676 | 27111 (31.66%) |
| 2A | 137150 | 0.251605 | 0.856962 | 79772 (58.16%) | 125450 | 0.289098 | 0.936772 | 68972 (54.98%) | 119450 | 0.288518 | 0.972951 | 57769 (48.36%) |
| 3A | 96450 | 0.130453 | 2.27878 | 44914 (46.57%) | 74950 | 0.134097 | 2.933748 | 28787 (38.41%) | 85000 | 0.15728 | 2.574908 | 25912 (30.48%) |
| 4A | 130500 | 0.317779 | 1.378513 | 79428 (60.86%) | 110850 | 0.369392 | 1.594492 | 66016 (59.55%) | 116700 | 0.36745 | 1.50704 | 58086 (49.77%) |
| 5A | 71850 | 0.132927 | 2.005721 | 32488 (45.22%) | 60100 | 0.146486 | 2.402626 | 24483 (40.74%) | 60600 | 0.166755 | 2.38547 | 18725 (30.9%) |
| 6A | 99050 | 0.158856 | 1.296073 | 52549 (53.05%) | 85850 | 0.178539 | 1.498357 | 40739 (47.45%) | 86550 | 0.178744 | 1.486057 | 29651 (34.26%) |
| 7A | 149700 | 0.193545 | 1.164988 | 78616 (52.52%) | 128550 | 0.211862 | 1.358487 | 64114 (49.87%) | 129900 | 0.232161 | 1.343972 | 49454 (38.07%) |
| 1B | 150800 | 0.154279 | 0.932852 | 80419 (53.33%) | 135600 | 0.154625 | 1.035051 | 64442 (47.52%) | 132400 | 0.20421 | 1.063407 | 49705 (37.54%) |
| 2B | 187300 | 0.156885 | 0.764253 | 102236 (54.58%) | 157350 | 0.176011 | 0.910909 | 79057 (50.24%) | 166950 | 0.19665 | 0.858127 | 66140 (39.62%) |
| 3B | 201700 | 0.210733 | 0.771726 | 119399 (59.2%) | 173200 | 0.220043 | 0.89872 | 90266 (52.12%) | 177550 | 0.243607 | 0.876084 | 78180 (44.03%) |
| 4B | 60050 | 0.115027 | 2.20477 | 23537 (39.2%) | 44800 | 0.09777 | 2.968273 | 12423 (27.73%) | 52600 | 0.142347 | 2.516753 | 13477 (25.62%) |
| 5B | 152400 | 0.15014 | 1.292476 | 80669 (52.93%) | 136300 | 0.14202 | 1.445522 | 57252 (42%) | 135650 | 0.202818 | 1.431617 | 55651 (41.03%) |
| 6 | 190850 | 0.13708 | 0.658245 | 99314 (52.04%) | 167500 | 0.135522 | 0.750676 | 71975 (42.97%) | 159700 | 0.203568 | 0.787671 | 66038 (41.35%) |
| 7B | 150100 | 0.121987 | 0.987127 | 70107 (46.71%) | 127550 | 0.12878 | 1.153868 | 51602 (40.46%) | 134150 | 0.155388 | 1.102364 | 41168 (30.69%) |
| 1D | 48650 | 0.238268 | 3.477302 | 26009 (53.46%) | 42500 | 0.226198 | 3.808863 | 20075 (47.24%) | 38350 | 0.285881 | 4.409069 | 16564 (43.19%) |
| 2D | 69550 | 0.183692 | 1.586178 | 31547 (45.36%) | 55400 | 0.163933 | 1.999469 | 21117 (38.12%) | 49600 | 0.228564 | 2.23156 | 16357 (32.98%) |
| 3D | 37050 | 0.116765 | 4.639072 | 5460 (14.74%) | 31800 | 0.165445 | 5.245984 | 11619 (36.54%) | 26800 | 0.137566 | 6.273779 | 5458 (20.37%) |
| 4D | 13500 | 0.122822 | 9.104484 | 4560 (33.78%) | 11800 | 0.130958 | 10.56137 | 3577 (30.31%) | 11550 | 0.154924 | 10.56621 | 2312 (20.02%) |
| 5D | 31750 | 0.130873 | 6.894582 | 12308 (38.77%) | 26250 | 0.134737 | 8.311197 | 9238 (35.19%) | 23700 | 0.147915 | 9.317761 | 5518 (23.28%) |
| 6D | 38300 | 0.123729 | 4.134238 | 15652 (40.87%) | 34900 | 0.136001 | 4.545476 | 12619 (36.16%) | 29750 | 0.137805 | 5.369092 | 6852 (23.03%) |
| 7D | 46700 | 0.150286 | 4.409549 | 17838 (38.2%) | 42300 | 0.147515 | 4.882439 | 14457 (34.18%) | 35850 | 0.201644 | 5.778975 | 10863 (30.3%) |
| A genome | 796275 | 0.195029 | 1.397647 | 417684 (52.45%) | 680325 | 0.220024 | 1.631824 | 328006 (48.21%) | 683825 | 0.232699 | 1.61945 | 266708 (39%) |
| B genome | 1093200 | 0.154972 | 0.95375 | 575681 (52.66%) | 942300 | 0.1588 | 1.106081 | 427017 (45.32%) | 959000 | 0.199661 | 1.084318 | 370359 (38.62%) |
| D genome | 285500 | 0.162046 | 4.054108 | 113374 (39.71%) | 244950 | 0.1634 | 4.684331 | 92702 (37.85%) | 215600 | 0.197637 | 5.369609 | 63924 (29.65%) |
| Whole genomes | 2174975 | 0.170566 | 1.523235 | 1106739 (50.89%) | 1867575 | 0.181706 | 1.766921 | 847725 (45.39%) | 1858425 | 0.211583 | 1.778371 | 700991 (37.72%) |

Abbreviations: r^2^: average squared allele frequency correlation TNSP: Total number of SNP pairs, NSSP: Number of significant SNP pairs (P<0.001).

**Supplementary Table 3**. A summary of QTN-by-environment interactions for some seed traits of Iranian wheat using 3VmrMLM

| Trait name | Marker | Sequence | Chro | Position (cM) | LOD (QE) | add*env1 | dom*env1 | add*env2 | dom*env2 | add*env3 | dom*env3 | add*env4 | dom*env4 | variance | r2(%) | P-value |  |  |  |  |
| --- | --- | --- | --- | --- | --- | --- | --- | --- | --- | --- | --- | --- | --- | --- | --- | --- | --- | --- | --- | --- |
| MaxR | rs4205 | TGCAGACGACGTGAGCGGCCGAGCCTTCGGCTTCATGGCACAGGGATGGCGGCGACACGGCAGT | 1D | 48.904 | 18.2016 | 0.0366 | 0.0425 | 0.0333 | 0.0521 | -0.0295 | -0.0775 | -0.0404 | -0.0172 | 0.0013 | 1.7722 | 5.80E-16 |  |  |  |  |
|  | rs53985 | TGCAGGTGCCGTGGGTGCAAAAACAACTCCTGCAACATTTTCATCCGTGCTTAAAGCTCTAAAT | 7D | 150.625 | 15.6859 | 0.0491 |  | 0.0098 |  | -0.0236 |  | -0.0353 |  | 0.0011 | 1.5197 | 1.42E-15 |  |  |  |  |
|  | rs2102 | TGCAGAAGCTTGGGATACTTGGAGTATAGCAAAGGGCGGCATGTGTATAAAATTTAGTTTTGTA | 1A | 34.278 | 7.0102 | 0.0058 | -0.0572 | 0.0009 | -0.0936 | -0.0004 | 0.0744 | -0.0063 | 0.0765 | 0.0002 | 0.5789 | 1.44E-05 |  |  |  |  |
|  | rs5425 | TGCAGAGAGAAAGAGGGGTTTCGGGCTACGCGGTTAACCGTTGGAGTACCAAACGACCTCCAAA | 2B | 59.184 | 7.557 | 0.0166 | 0.0518 | 0.0074 | 0.001 | -0.011 | -0.0154 | -0.0131 | -0.0374 | 0.0002 | 0.6247 | 4.71E-06 |  |  |  |  |
|  | rs33742 | TGCAGCGTGCCTTGCAATGGCGTGCGCAACCTACAAGCAAAATTACACAAGATGCCAAATTACA | 6A | 99.391 | 8.3549 | -0.0091 | -0.0108 | -0.0173 | 0.0064 | 0.0096 | -0.0117 | 0.0168 | 0.0161 | 0.0002 | 0.6918 | 9.07E-07 |  |  |  |  |
|  | rs5421 | TGCAGAGAGAAAAAGCACCATCATCAAAGCAATATCCAGCAATAACCAATGAAAAAAAACCCAC | 7D | 71.943 | 11.7923 | 0.0191 | -0.0423 | 0.008 | 0.0062 | -0.003 | 0.0576 | -0.0242 | -0.0215 | 0.0003 | 0.9829 | 6.41E-10 |  |  |  |  |
| MBCRadius | rs4205 | TGCAGACGACGTGAGCGGCCGAGCCTTCGGCTTCATGGCACAGGGATGGCGGCGACACGGCAGT | 1D | 48.904 | 19.1461 | 0.0506 | 0.0321 | 0.046 | -0.0117 | -0.0374 | -0.0396 | -0.0592 | 0.0192 | 0.0024 | 1.9759 | 7.28E-17 |  |  |  |  |
|  | rs38918 | TGCAGCTGCCTGCTCATCCTCACCATCTCAATCTGTGTACGAGTAGATCCCATGGTTTACCAGT | 2A | 11.39 | 24.6463 | -0.0602 | -0.1062 | -0.04 | -0.116 | 0.0609 | 0.0852 | 0.0392 | 0.137 | 0.0031 | 2.571 | 3.77E-22 |  |  |  |  |
|  | rs31109 | TGCAGCGCTCGAAGTACAGCGTCCACGGCGTTTCAATGTCGGACGCGTACCTCGGCTCGTTCCG | 2B | 111.506 | 14.7386 | -0.0208 | -0.0511 | -0.0571 | -0.0122 | 0.0225 | 0.0583 | 0.0554 | 0.0049 | 0.0018 | 1.508 | 1.12E-12 |  |  |  |  |
|  | rs38828 | TGCAGCTGCCGAGGTACGCTTCGACTTGAAGTTCGAGAATGTGCCCTTGGTGATCCTCTACATG | 4A | 147.563 | 8.248 | 0.0276 | -0.0102 | 0.036 | -0.0272 | -0.0339 | 0.0265 | -0.0297 | 0.0108 | 0.001 | 0.8333 | 1.13E-06 |  |  |  |  |
|  | rs5930 | TGCAGAGCATGATCAGCTTCAGCAGTTCGACAAGCACACGCACCATGGGAGAAAGGTTGCACAT | 4B | 93.598 | 15.3714 | -0.0334 | -0.2705 | -0.0244 | -0.122 | 0.0505 | 0.1797 | 0.0074 | 0.2128 | 0.0019 | 1.5747 | 2.82E-13 |  |  |  |  |
|  | rs5421 | TGCAGAGAGAAAAAGCACCATCATCAAAGCAATATCCAGCAATAACCAATGAAAAAAAACCCAC | 7D | 71.943 | 8.8372 | 0.0448 | -0.0675 | 0.0115 | 0.0806 | -0.0122 | 0.0237 | -0.0441 | -0.0369 | 0.0011 | 0.8939 | 3.32E-07 |  |  |  |  |
| CHull | rs4205 | TGCAGACGACGTGAGCGGCCGAGCCTTCGGCTTCATGGCACAGGGATGGCGGCGACACGGCAGT | 1D | 48.904 | 17.8821 | 0.1568 | 0.382 | 0.1453 | 0.1778 | -0.1188 | -0.3971 | -0.1833 | -0.1627 | 0.0242 | 1.8588 | 1.17E-15 |  |  |  |  |
|  | rs38828 | TGCAGCTGCCGAGGTACGCTTCGACTTGAAGTTCGAGAATGTGCCCTTGGTGATCCTCTACATG | 4A | 147.563 | 5.2778 | 0.0617 | -0.0123 | 0.1054 | 0.003 | -0.0815 | 0.0086 | -0.0856 | 0.0007 | 0.007 | 0.5353 | 0.000459 |  |  |  |  |
|  | rs62825 | TGCAGTTCAAAGGAGTTCAATGGAAAGGGCGGGTCGGGGCTTATAAACAGGTCCTGCCGCTCCT | 7A | 0.5685 | 6.3869 | 0.1052 | 0.0582 | 0.0806 | -0.0649 | -0.0921 | -0.0225 | -0.0937 | 0.0292 | 0.0085 | 0.6492 | 5.08E-05 |  |  |  |  |
| CArea | rs47935 | TGCAGGCGCCACTTATCCTAATCGGCCAATAAGGCCAACTCCAACGCGCACCCCCATCATGTCT | 2B | 59.184 | 18.3265 | 0.3714 | 0.4688 | 0.2556 | 0.5582 | -0.3626 | -0.5464 | -0.2644 | -0.4806 | 0.1039 | 1.7933 | 4.41E-16 |  |  |  |  |
|  | rs24480 | TGCAGCCCTGATGGCTCTGCTGAAACATTCTGGACCCGAAACATGAACTCTACTGCCAGTCGGA | 2B | 111.506 | 8.0127 | -0.0498 | 0.1133 | -0.3023 | -0.4949 | 0.0904 | -0.2613 | 0.2616 | 0.6429 | 0.0445 | 0.7685 | 1.84E-06 |  |  |  |  |
|  | rs38828 | TGCAGCTGCCGAGGTACGCTTCGACTTGAAGTTCGAGAATGTGCCCTTGGTGATCCTCTACATG | 4A | 147.563 | 5.2772 | 0.1345 | 0.0578 | 0.2076 | -0.0836 | -0.1765 | 0.1025 | -0.1657 | -0.0766 | 0.0292 | 0.5034 | 0.00046 |  |  |  |  |
|  | rs62825 | TGCAGTTCAAAGGAGTTCAATGGAAAGGGCGGGTCGGGGCTTATAAACAGGTCCTGCCGCTCCT | 7A | 0.5685 | 5.4302 | 0.2007 | 0.0594 | 0.1465 | -0.163 | -0.1495 | 0.0097 | -0.1977 | 0.094 | 0.03 | 0.5182 | 0.000341 |  |  |  |  |
|  | rs5421 | TGCAGAGAGAAAAAGCACCATCATCAAAGCAATATCCAGCAATAACCAATGAAAAAAAACCCAC | 7D | 71.943 | 17.0469 | 0.3904 | -0.6075 | 0.1818 | 0.295 | -0.1553 | 0.4889 | -0.4168 | -0.1764 | 0.0964 | 1.6639 | 7.29E-15 |  |  |  |  |
| ArBBox | rs34997 | TGCAGCTAGCGTGAGTGCGAGCAAGAGCCGAGATCGGAAGAGCGGGATCACCGACTGCCCATAG | 1A | 44.512 | 8.461 | 0.2052 | 0.829 | -0.001 | 1.162 | -0.1348 | -1.0561 | -0.0694 | -0.935 | 0.0661 | 0.7215 | 7.28E-07 |  |  |  |  |
|  | rs38918 | TGCAGCTGCCTGCTCATCCTCACCATCTCAATCTGTGTACGAGTAGATCCCATGGTTTACCAGT | 2A | 11.39 | 26.2039 | -0.5139 | -0.7426 | -0.3316 | -0.9077 | 0.505 | 0.5079 | 0.3405 | 1.1424 | 0.212 | 2.3134 | 1.18E-23 |  |  |  |  |
|  | rs24480 | TGCAGCCCTGATGGCTCTGCTGAAACATTCTGGACCCGAAACATGAACTCTACTGCCAGTCGGA | 2B | 111.506 | 24.1188 | -0.2739 | -0.4755 | -0.5555 | -1.0887 | 0.3163 | 0.3491 | 0.5131 | 1.2151 | 0.1943 | 2.1206 | 1.22E-21 |  |  |  |  |
|  | rs38828 | TGCAGCTGCCGAGGTACGCTTCGACTTGAAGTTCGAGAATGTGCCCTTGGTGATCCTCTACATG | 4A | 147.563 | 5.4724 | 0.1441 | -0.0858 | 0.2572 | -0.3235 | -0.2115 | 0.3541 | -0.1898 | 0.0551 | 0.0425 | 0.464 | 0.000313 |  |  |  |  |
|  | rs53693 | TGCAGGTGACCACGCTAGCACCGACGGCAGCGCGAGAAACGGACGACGACTCCCGTCGCGGTCC | 5B | 113.896 | 15.8508 | 0.4057 | -1.2326 | 0.2476 | -0.6665 | -0.2826 | 1.6959 | -0.3707 | 0.2032 | 0.1257 | 1.3713 | 9.93E-14 |  |  |  |  |
|  | rs14446 | TGCAGCACCGCCCGCCGCGCAGTGCGTGCCGCCACGCCCGACACTGCCGTGCACTGCCAAGCCG | 7A | 1.137 | 9.3593 | 0.3131 | 0.0203 | 0.2348 | 0.2475 | -0.2735 | -0.25 | -0.2744 | -0.0177 | 0.0733 | 0.7995 | 1.11E-07 |  |  |  |  |
|  | rs5421 | TGCAGAGAGAAAAAGCACCATCATCAAAGCAATATCCAGCAATAACCAATGAAAAAAAACCCAC | 7D | 71.943 | 15.491 | 0.4208 | -1.324 | 0.1839 | 0.043 | -0.1088 | 1.4162 | -0.4959 | -0.1352 | 0.1227 | 1.3392 | 2.18E-13 |  |  |  |  |
| Aspect_ratio | rs4507 | TGCAGACGGCGGTGAACGCCAACACGTGGCATATCGCCAAGTACCTTGACGGCTCGGTACAGAC | D | 29.561 | 9.3637 | -0.0058 | -0.1404 | 0.0032 | -0.2031 | 0.0026 | 0.1322 | 0.0001 | 0.2113 | 0.0005 | 0.6471 | 1.10E-07 |  |  |  |  |
|  | rs30886 | TGCAGCGCGTCCCCGCCCTTTCCCGCCACGCGTCTACACCGAGATCGGAAGAGCGGGATCACCG | 6B | 2.273 | 7.3351 | 0.0111 | -0.114 | 0.0095 | -0.0319 | -0.0199 | 0.0271 | -0.0007 | 0.1188 | 0.0004 | 0.5049 | 7.42E-06 |  |  |  |  |
|  | rs5644 | TGCAGAGATCCATTATATTACATCATCTGCAAGCTGCCCGAGATCGGAAGAGCGGGATCACCGA | 6B | 23.949 | 8.6365 | 0.0002 | -0.0924 | 0.01 | -0.1318 | -0.008 | 0.1449 | -0.0022 | 0.0793 | 0.0005 | 0.596 | 5.05E-07 |  |  |  |  |
| Circ | rs38918 | TGCAGCTGCCTGCTCATCCTCACCATCTCAATCTGTGTACGAGTAGATCCCATGGTTTACCAGT | 2A | 11.39 | 22.1318 | -4.7844 | -6.9299 | -3.1732 | -7.7168 | 4.7559 | 4.5909 | 3.2016 | 10.0558 | 18.3824 | 2.0318 | 9.99E-20 |  |  |  |  |
|  | rs47935 | TGCAGGCGCCACTTATCCTAATCGGCCAATAAGGCCAACTCCAACGCGCACCCCCATCATGTCT | 2B | 59.184 | 13.7438 | 3.8004 | 4.7735 | 2.7336 | 6.0381 | -3.7195 | -5.8199 | -2.8145 | -4.9917 | 11.2298 | 1.2412 | 9.63E-12 |  |  |  |  |
|  | rs24480 | TGCAGCCCTGATGGCTCTGCTGAAACATTCTGGACCCGAAACATGAACTCTACTGCCAGTCGGA | 2B | 111.506 | 10.603 | -1.0346 | 0.3595 | -4.0572 | -7.044 | 1.4365 | -2.6413 | 3.6553 | 9.3258 | 8.6107 | 0.9517 | 8.08E-09 |  |  |  |  |
|  | rs38828 | TGCAGCTGCCGAGGTACGCTTCGACTTGAAGTTCGAGAATGTGCCCTTGGTGATCCTCTACATG | 4A | 147.563 | 6.9031 | 2.0553 | 1.1292 | 2.6991 | -0.6363 | -2.5439 | 0.9936 | -2.2105 | -1.4865 | 5.5659 | 0.6152 | 1.79E-05 |  |  |  |  |
|  | rs62825 | TGCAGTTCAAAGGAGTTCAATGGAAAGGGCGGGTCGGGGCTTATAAACAGGTCCTGCCGCTCCT | 7A | 0.5685 | 5.5317 | 2.5158 | 1.1698 | 1.706 | -1.7941 | -1.8705 | -0.2631 | -2.3512 | 0.8874 | 4.4483 | 0.4917 | 0.000279 |  |  |  |  |
|  | rs5421 | TGCAGAGAGAAAAAGCACCATCATCAAAGCAATATCCAGCAATAACCAATGAAAAAAAACCCAC | 7D | 71.943 | 14.4176 | 4.3489 | -9.6468 | 1.8195 | 3.4149 | -1.5224 | 9.4117 | -4.6461 | -3.1798 | 11.7959 | 1.3038 | 2.24E-12 |  |  |  |  |
| Roundness | rs13949 | TGCAGCACCAACCACAAAACAAGACATAAACTGTCCG | 4A | 112.315 | 9.4972 | 0.0102 | -0.127 | 0.0111 | -0.2532 | -0.0195 | 0.1941 | -0.0017 | 0.1861 | 0.0005 | 0.6911 | 8.34E-08 |  |  |  |  |
|  | rs52026 | TGCAGGGTTGCTTGCAGGTTGTACCTTCACCGAAATGATCAGATGATCATAGCCCGAGATCGGA | 5A | 93.664 | 3.1574 | -0.0003 | -0.027 | 0 | -0.0507 | -0.0008 | 0.0814 | 0.0011 | -0.0037 | 0.0002 | 0.2269 | 0.024154 |  |  |  |  |
|  | rs30886 | TGCAGCGCGTCCCCGCCCTTTCCCGCCACGCGTCTACACCGAGATCGGAAGAGCGGGATCACCG | 6B | 2.273 | 8.1589 | 0.0092 | -0.127 | 0.0058 | -0.042 | -0.0179 | 0.0308 | 0.0029 | 0.1382 | 0.0005 | 0.5921 | 1.36E-06 |  |  |  |  |
|  | rs5644 | TGCAGAGATCCATTATATTACATCATCTGCAAGCTGCCCGAGATCGGAAGAGCGGGATCACCGA | 6B | 23.949 | 5.0047 | 0.0018 | -0.07 | 0.0101 | -0.0915 | -0.0106 | 0.0994 | -0.0012 | 0.0621 | 0.0003 | 0.361 | 0.000781 |  |  |  |  |
| ArEquivD | rs47935 | TGCAGGCGCCACTTATCCTAATCGGCCAATAAGGCCAACTCCAACGCGCACCCCCATCATGTCT | 2B | 59.184 | 18.1182 | 0.0517 | 0.0526 | 0.0378 | 0.0653 | -0.0517 | -0.0684 | -0.0378 | -0.0495 | 0.0021 | 1.7752 | 6.96E-16 |  |  |  |  |
|  | rs24480 | TGCAGCCCTGATGGCTCTGCTGAAACATTCTGGACCCGAAACATGAACTCTACTGCCAGTCGGA | 2B | 111.506 | 12.4391 | -0.0193 | 0.0006 | -0.0488 | -0.0703 | 0.0196 | -0.0365 | 0.0484 | 0.1062 | 0.0014 | 1.2053 | 1.60E-10 |  |  |  |  |
|  | rs38828 | TGCAGCTGCCGAGGTACGCTTCGACTTGAAGTTCGAGAATGTGCCCTTGGTGATCCTCTACATG | 4A | 147.563 | 7.0675 | 0.0248 | 0.0124 | 0.0321 | -0.0102 | -0.029 | 0.0045 | -0.028 | -0.0067 | 0.0008 | 0.6777 | 1.28E-05 |  |  |  |  |
|  | rs5421 | TGCAGAGAGAAAAAGCACCATCATCAAAGCAATATCCAGCAATAACCAATGAAAAAAAACCCAC | 7D | 71.943 | 15.4863 | 0.0529 | -0.0463 | 0.025 | 0.0593 | -0.0221 | 0.047 | -0.0558 | -0.06 | 0.0018 | 1.5096 | 2.20E-13 |  |  |  |  |
| PerEquivD | rs38918 | TGCAGCTGCCTGCTCATCCTCACCATCTCAATCTGTGTACGAGTAGATCCCATGGTTTACCAGT | 2A | 11.39 | 22.1318 | -0.1212 | -0.1755 | -0.0804 | -0.1955 | 0.1205 | 0.1163 | 0.0811 | 0.2547 | 0.0118 | 2.0318 | 9.99E-20 |  |  |  |  |
|  | rs47935 | TGCAGGCGCCACTTATCCTAATCGGCCAATAAGGCCAACTCCAACGCGCACCCCCATCATGTCT | 2B | 59.184 | 13.7438 | 0.0963 | 0.1209 | 0.0692 | 0.153 | -0.0942 | -0.1474 | -0.0713 | -0.1264 | 0.0072 | 1.2412 | 9.63E-12 |  |  |  |  |
|  | rs24480 | TGCAGCCCTGATGGCTCTGCTGAAACATTCTGGACCCGAAACATGAACTCTACTGCCAGTCGGA | 2B | 111.506 | 10.603 | -0.0262 | 0.0091 | -0.1028 | -0.1784 | 0.0364 | -0.0669 | 0.0926 | 0.2362 | 0.0055 | 0.9518 | 8.08E-09 |  |  |  |  |
|  | rs38828 | TGCAGCTGCCGAGGTACGCTTCGACTTGAAGTTCGAGAATGTGCCCTTGGTGATCCTCTACATG | 4A | 147.563 | 6.9031 | 0.0521 | 0.0286 | 0.0684 | -0.0161 | -0.0644 | 0.0252 | -0.056 | -0.0377 | 0.0036 | 0.6152 | 1.79E-05 |  |  |  |  |
|  | rs62825 | TGCAGTTCAAAGGAGTTCAATGGAAAGGGCGGGTCGGGGCTTATAAACAGGTCCTGCCGCTCCT | 7A | 0.5685 | 5.5317 | 0.0637 | 0.0296 | 0.0432 | -0.0454 | -0.0474 | -0.0067 | -0.0596 | 0.0225 | 0.0029 | 0.4917 | 0.000279 |  |  |  |  |
|  | rs5421 | TGCAGAGAGAAAAAGCACCATCATCAAAGCAATATCCAGCAATAACCAATGAAAAAAAACCCAC | 7D | 71.943 | 14.4177 | 0.1102 | -0.2444 | 0.0461 | 0.0865 | -0.0386 | 0.2384 | -0.1177 | -0.0806 | 0.0076 | 1.3038 | 2.24E-12 |  |  |  |  |
| EquivEllAr | rs38918 | TGCAGCTGCCTGCTCATCCTCACCATCTCAATCTGTGTACGAGTAGATCCCATGGTTTACCAGT | 2A | 11.39 | 25.1193 | -0.406 | -0.6749 | -0.2504 | -0.7858 | 0.3925 | 0.5007 | 0.264 | 0.96 | 0.1339 | 2.3666 | 1.32E-22 |  |  |  |  |
|  | rs24480 | TGCAGCCCTGATGGCTCTGCTGAAACATTCTGGACCCGAAACATGAACTCTACTGCCAGTCGGA | 2B | 111.506 | 27.8786 | -0.2606 | -0.4241 | -0.4708 | -1.148 | 0.2977 | 0.4991 | 0.4337 | 1.0729 | 0.1494 | 2.6408 | 2.82E-25 |  |  |  |  |
|  | rs53693 | TGCAGGTGACCACGCTAGCACCGACGGCAGCGCGAGAAACGGACGACGACTCCCGTCGCGGTCC | 5B | 113.896 | 17.4429 | 0.3431 | -0.9125 | 0.2268 | -0.4914 | -0.2517 | 1.2949 | -0.3182 | 0.1091 | 0.0916 | 1.6188 | 3.06E-15 |  |  |  |  |
|  | rs14446 | TGCAGCACCGCCCGCCGCGCAGTGCGTGCCGCCACGCCCGACACTGCCGTGCACTGCCAAGCCG | 7A | 1.137 | 11.9257 | 0.274 | 0.0834 | 0.225 | 0.3101 | -0.2528 | -0.3142 | -0.2462 | -0.0793 | 0.062 | 1.095 | 4.82E-10 |  |  |  |  |
|  | rs5421 | TGCAGAGAGAAAAAGCACCATCATCAAAGCAATATCCAGCAATAACCAATGAAAAAAAACCCAC | 7D | 71.943 | 16.1615 | 0.3613 | -0.9047 | 0.1559 | 0.2879 | -0.1162 | 0.8807 | -0.401 | -0.2639 | 0.0847 | 1.4962 | 5.04E-14 |  |  |  |  |
| Compactness | rs125 | TGCAGAAAACAGAGCTTCCCTCGTCTCTGCTTTAGCTGCCCAGTTTGACTTATGCACCTTGTAT | 6A | 0 | 33.6616 | -0.0034 | -0.0114 | -0.0075 | 0.0017 | 0.0028 | 0.007 | 0.0081 | 0.0026 | 0 | 2.1552 | 6.74E-31 |  |  |  |  |
|  | rs30886 | TGCAGCGCGTCCCCGCCCTTTCCCGCCACGCGTCTACACCGAGATCGGAAGAGCGGGATCACCG | 6B | 2.273 | 7.3428 | -0.0012 | 0.0164 | -0.0009 | 0.0069 | 0.002 | -0.0067 | 0.0002 | -0.0165 | 0 | 0.4465 | 7.31E-06 |  |  |  |  |
| Perim | rs4205 | TGCAGACGACGTGAGCGGCCGAGCCTTCGGCTTCATGGCACAGGGATGGCGGCGACACGGCAGT | 1D | 48.904 | 17.0228 | 0.1831 | 0.3589 | 0.1567 | 0.1485 | -0.1376 | -0.4066 | -0.2022 | -0.1008 | 0.0302 | 2.0284 | 7.68E-15 |  |  |  |  |
|  | rs38828 | TGCAGCTGCCGAGGTACGCTTCGACTTGAAGTTCGAGAATGTGCCCTTGGTGATCCTCTACATG | 4A | 147.563 | 6.0332 | 0.0667 | -0.0411 | 0.1354 | -0.0135 | -0.0994 | 0.0423 | -0.1027 | 0.0123 | 0.0105 | 0.7037 | 0.000103 |  |  |  |  |
| Solidity | rs15614 | TGCAGCACTTAGTGGCATAATACAAGAGCAGAAATAGGCACACACTATTCTCGTGGGCGGACAT | 1B | 45.574 | 5.0577 | 0.0001 | -0.0055 | 0.0001 | -0.0009 | -0.0001 | 0.0039 | -0.0001 | 0.0026 | 0 | 0.5913 | 0.000705 |  |  |  |  |
|  | rs8556 | TGCAGATCGATCAGTTCCCAGAGGCAAACAACTTGGGAGCCAGCTCGAGCAGGGCATCGAGCGC | 3A | 17.079 | 7.4031 | 0.0007 | 0.0011 | 0.0004 | 0.0003 | -0.0006 | -0.0009 | -0.0005 | -0.0005 | 0 | 0.8695 | 6.46E-06 |  |  |  |  |
|  | rs27833 | TGCAGCGAAACCCAGGTGGGCTGCAACGGGAGTCGACCATGAGAACTTCTGCCGTGGGGTCACA | 3B | 75.087 | 12.6913 | 0.0009 | 0.0016 | 0.0005 | 0.0002 | -0.0007 | -0.0001 | -0.0007 | -0.0017 | 0 | 1.5059 | 9.32E-11 |  |  |  |  |
|  | rs15115 | TGCAGCACGGAAGGAGAGGGCCTAGCCACCTGGTACACTTGCTGGGGCGCGGGCAGACGCGGAC | 5B | 145.902 | 6.4669 | -0.0001 | -0.0092 | 0.0001 | 0.0022 | 0 | 0.0028 | 0.0001 | 0.0042 | 0 | 0.7581 | 4.33E-05 |  |  |  |  |
|  | rs8994 | TGCAGATGACGCGGGATGTCGTCCCCGTTCGAACAAGACCATCCGTCGTCGTTTTGGTCATAGT | 6B | 48.967 | 13.9506 | 0.001 | 0.0021 | 0.0001 | 0.0019 | -0.0006 | -0.0054 | -0.0005 | 0.0014 | 0 | 1.6594 | 6.16E-12 |  |  |  |  |
|  | rs8644 | TGCAGATCGGGTTCTACCACGACAGGTGCCCCTAGACGGAGGCCATCGTCAAGGGCGTTATGAT | 7A | 32.091 | 12.4389 | 0.0012 | -0.0012 | -0.0001 | 0.0032 | -0.0005 | -0.003 | -0.0006 | 0.001 | 0 | 1.4753 | 1.60E-10 |  |  |  |  |
|  | rs49346 | TGCAGGCTTTCCAGCTGAACAGCTGAATCACAGGGTATACACTGCGTTAGGATTCTTAACTTTG | 7A | 69.63 | 13.1062 | 0.0001 | -0.016 | -0.0001 | 0.0054 | -0.0002 | 0.0068 | 0.0002 | 0.0038 | 0 | 1.5564 | 3.81E-11 |  |  |  |  |
|  | rs25362 | TGCAGCCGCCGCGTGGGCCGCCCACAGCTGGTGGAACCTCTCCCGAGATCGGAAGAGCGGGATC | 7B | 52.33 | 14.5416 | 0.0001 | 0.0045 | -0.0001 | 0.0027 | 0 | -0.0032 | 0.0001 | -0.004 | 0 | 1.7317 | 1.71E-12 |  |  |  |  |
| Concavity | rs8180 | TGCAGATCCAAAGCTACCCCGATCTCCACCTTGGACCATTCCAAGGCGATGCAGAGGGCAAGCT | 1B | 66.042 | 16.7437 | -0.014 | -0.0829 | -0.0002 | -0.0487 | 0.0111 | 0.0493 | 0.003 | 0.0824 | 0.0002 | 2.2085 | 1.41E-14 |  |  |  |  |
|  | rs65526 | TGCAGTTTTTGCACGCGGCACAAACATCTTCGTCTTCCTCTCTCATGTGAGCTCGAGCGTTTAG | 2B | 25.594 | 10.5749 | -0.0054 | -0.0891 | -0.0007 | -0.0328 | -0.0038 | 0.0628 | 0.0099 | 0.0591 | 0.0001 | 1.3782 | 8.57E-09 |  |  |  |  |
|  | rs20199 | TGCAGCATTACAAACCACCACCATCCTCGACCCGAGCGCGACAGCGTGGGGGCCGAGATCGGAA | 2B | 59.184 | 22.0029 | -0.0119 | -0.157 | -0.0023 | -0.0353 | 0.0049 | 0.0892 | 0.0093 | 0.1031 | 0.0002 | 2.9322 | 1.33E-19 |  |  |  |  |
|  | rs13332 | TGCAGCACAACAACACAAGATGCGCCACAAAGATTAGAAACAGGCCCGAAAGCAAGCAAGTAAA | 3A | 43.437 | 17.7009 | 0.0039 | 0.1711 | -0.0085 | 0.0279 | 0.0029 | -0.0659 | 0.0017 | -0.133 | 0.0002 | 2.3391 | 1.74E-15 |  |  |  |  |
|  | rs27833 | TGCAGCGAAACCCAGGTGGGCTGCAACGGGAGTCGACCATGAGAACTTCTGCCGTGGGGTCACA | 3B | 75.087 | 11.6935 | -0.0153 | -0.0265 | -0.0056 | -0.0083 | 0.0101 | 0.0121 | 0.0108 | 0.0226 | 0.0001 | 1.5273 | 7.91E-10 |  |  |  |  |
|  | rs15115 | TGCAGCACGGAAGGAGAGGGCCTAGCCACCTGGTACACTTGCTGGGGCGCGGGCAGACGCGGAC | 5B | 145.902 | 9.7898 | 0.0095 | 0.1405 | 0.0023 | -0.0539 | -0.0067 | -0.029 | -0.005 | -0.0576 | 0.0001 | 1.2739 | 4.51E-08 |  |  |  |  |
|  | rs62410 | TGCAGTGTTTTGTAAAAGAGCGGACGTGTGAGGCGAACTAGGCAAATCAATCAATGGTTACACG | 6A | 0 | 6.697 | 0.0016 | 0.0921 | -0.001 | -0.0027 | -0.003 | -0.0698 | 0.0024 | -0.0197 | 0.0001 | 0.8662 | 2.72E-05 |  |  |  |  |
|  | rs50928 | TGCAGGGGAGCGCGGCGAGCGGTGGCTGCGTCATGACGCGGCGGCCAGATCCGAGATCGGAAGA | 6B | 94.461 | 9.1817 | -0.0121 | 0.0202 | -0.0071 | -0.0144 | 0.0085 | -0.0153 | 0.0106 | 0.0094 | 0.0001 | 1.1934 | 1.62E-07 |  |  |  |  |
|  | rs49346 | TGCAGGCTTTCCAGCTGAACAGCTGAATCACAGGGTATACACTGCGTTAGGATTCTTAACTTTG | 7A | 69.63 | 23.1858 | 0.0067 | 0.3265 | -0.0003 | -0.1351 | 0.0005 | -0.1033 | -0.007 | -0.0881 | 0.0003 | 3.097 | 9.66E-21 |  |  |  |  |
| Convexity | rs9654 | TGCAGATGTACGAGTGGGGACGAATCTTAGCCGTTGCATAAAAATCTGACGGTACTTAAAAAAC | 1A | 87.736 | 10.5415 | 0.0006 | -0.012 | 0.0003 | 0.0053 | -0.0003 | 0.0018 | -0.0005 | 0.0049 | 0 | 2.0185 | 9.20E-09 |  |  |  |  |
|  | rs35884 | TGCAGCTCACGTAGAAGGAGACCCGACCGACAGCGCGATTCGCAAGACAGTCGACGAGCGCTTT | 1B | 75.148 | 16.9999 | -0.0001 | 0.0074 | -0.0001 | -0.0222 | 0.0004 | 0.0071 | -0.0002 | 0.0077 | 0 | 3.2964 | 8.08E-15 |  |  |  |  |
|  | rs13332 | TGCAGCACAACAACACAAGATGCGCCACAAAGATTAGAAACAGGCCCGAAAGCAAGCAAGTAAA | 3A | 43.437 | 6.3256 | -0.0002 | -0.0086 | -0.0001 | 0.0004 | 0.0001 | 0.0025 | 0.0002 | 0.0058 | 0 | 1.2014 | 5.75E-05 |  |  |  |  |
|  | rs7643 | TGCAGATACCCCTACCGACACCATGTCTCTTTCTTACTTATTTCTTAATCAATTGTGATCTTTT | 3B | 22.764 | 39.7108 | 0.0028 | -0.0007 | -0.0001 | 0.0022 | -0.0011 | 0.0005 | -0.0015 | -0.002 | 0 | 8.052 | 8.34E-37 |  |  |  |  |
|  | rs10599 | TGCAGATTTTGGGTTTAGTCCCACTTTTAACATTGTAGATTTGAAGCCTTATTTGGGTGACGAG | 3B | 48.935 | 12.9176 | -0.0008 | -0.0239 | 0.0002 | 0.0086 | 0.0004 | 0.0071 | 0.0003 | 0.0082 | 0 | 2.485 | 5.73E-11 |  |  |  |  |
|  | rs15115 | TGCAGCACGGAAGGAGAGGGCCTAGCCACCTGGTACACTTGCTGGGGCGCGGGCAGACGCGGAC | 5B | 145.902 | 12.7877 | -0.0003 | -0.015 | 0 | 0.0087 | 0.0003 | 0.0037 | 0 | 0.0025 | 0 | 2.4594 | 7.57E-11 |  |  |  |  |
|  | rs31905 | TGCAGCGGCCGCCTAGACACCCAGGGCCAGGGATCTGACGGTTAGCTTTAGCTTAATCGAACGG | 5B | 145.902 | 6.0769 | 0.0004 | -0.0065 | 0.0003 | 0.0023 | -0.0005 | 0.002 | -0.0002 | 0.0022 | 0 | 1.1536 | 9.46E-05 |  |  |  |  |
|  | rs49346 | TGCAGGCTTTCCAGCTGAACAGCTGAATCACAGGGTATACACTGCGTTAGGATTCTTAACTTTG | 7A | 69.63 | 11.3531 | 0 | -0.0148 | 0.0004 | 0.0133 | -0.0002 | -0.0018 | -0.0002 | 0.0034 | 0 | 2.1774 | 1.64E-09 |  |  |  |  |
| Shape | rs9654 | TGCAGATGTACGAGTGGGGACGAATCTTAGCCGTTGCATAAAAATCTGACGGTACTTAAAAAAC | 1A | 87.736 | 8.6539 | -0.0405 | 1.7902 | 0.0354 | -0.5559 | -0.0533 | -0.7687 | 0.0584 | -0.4657 | 0.0131 | 0.6138 | 4.87E-07 |  |  |  |  |
|  | rs15614 | TGCAGCACTTAGTGGCATAATACAAGAGCAGAAATAGGCACACACTATTCTCGTGGGCGGACAT | 1B | 45.574 | 5.8216 | 0.0353 | 1.0429 | -0.005 | 0.2526 | -0.0199 | -0.6233 | -0.0104 | -0.6722 | 0.0088 | 0.4106 | 0.000157 |  |  |  |  |
|  | rs8980 | TGCAGATGACATTGTGCTCTCTGTCGCAGCAAGAGATACATAGTCGTGCCCGAGATCGGAAGAG | 2A | 76.592 | 6.2342 | 0.1011 | -0.3989 | 0.0167 | -0.1149 | -0.0453 | 0.187 | -0.0725 | 0.3268 | 0.0094 | 0.4401 | 6.91E-05 |  |  |  |  |
|  | rs15115 | TGCAGCACGGAAGGAGAGGGCCTAGCCACCTGGTACACTTGCTGGGGCGCGGGCAGACGCGGAC | 5B | 145.902 | 6.67 | -0.0315 | 1.6178 | -0.0139 | -0.1716 | 0.0389 | -0.5711 | 0.0065 | -0.8751 | 0.0101 | 0.4712 | 2.87E-05 |  |  |  |  |
|  | rs30886 | TGCAGCGCGTCCCCGCCCTTTCCCGCCACGCGTCTACACCGAGATCGGAAGAGCGGGATCACCG | 6B | 2.273 | 6.2919 | 0.0202 | -0.6262 | 0.0516 | -0.0879 | -0.0802 | 0.1061 | 0.0083 | 0.608 | 0.0095 | 0.4442 | 6.15E-05 |  |  |  |  |
|  | rs5644 | TGCAGAGATCCATTATATTACATCATCTGCAAGCTGCCCGAGATCGGAAGAGCGGGATCACCGA | 6B | 23.949 | 6.1169 | -0.0145 | -0.3329 | 0.0231 | -0.6071 | -0.0149 | 0.7453 | 0.0062 | 0.1947 | 0.0092 | 0.4317 | 8.73E-05 |  |  |  |  |
|  | rs36297 | TGCAGCTCCACGTCGGTAGTGTCGCGGGTGAGGCTTTTCTGTTCATCAGCACGGACCGAGATCG | 6B | 48.967 | 10.4225 | 0.1544 | 0.0372 | 0.095 | 0.0383 | -0.1394 | 0.0172 | -0.11 | -0.0927 | 0.0159 | 0.7417 | 1.18E-08 |  |  |  |  |
| RFactor | rs49637 | TGCAGGGACGCACACAAAATATTCAGGATCCAAAACACCAAGACAGTGACTTGGACTACTAAAC | 3B | 60.303 | 5.7404 | -0.0016 | 0.0015 | -0.0004 | 0.0031 | 0.0006 | -0.0031 | 0.0014 | -0.0014 | 0 | 0.3715 | 0.000185 |  |  |  |  |
|  | rs52197 | TGCAGGTAAGGGAGGGAAGGGTCGAAGGGGGATGCGCCACCCGAGACGCTGGCGAAGTGGTGGC | 4A | 146.426 | 4.6341 | 0 | 0 | 0.0018 | -0.0013 | -0.001 | 0.0014 | -0.0009 | -0.0001 | 0 | 0.2993 | 0.001594 |  |  |  |  |
|  | rs64260 | TGCAGTTGGAAGGAGGGAGTTGTCATCTTCCTTTGGTGGAACCTAGCATGCGCGCTTCACCACT | 5B | 155.004 | 7.9055 | 0.0009 | 0.001 | 0.0019 | 0.001 | -0.001 | -0.001 | -0.0018 | -0.001 | 0 | 0.5138 | 2.30E-06 |  |  |  |  |
|  | rs125 | TGCAGAAAACAGAGCTTCCCTCGTCTCTGCTTTAGCTGCCCAGTTTGACTTATGCACCTTGTAT | 6A | 0 | 37.588 | -0.0014 | -0.0068 | -0.0046 | 0.0007 | 0.0019 | 0.0008 | 0.0041 | 0.0053 | 0 | 2.5895 | 9.93E-35 |  |  |  |  |
| ModRatio | rs125 | TGCAGAAAACAGAGCTTCCCTCGTCTCTGCTTTAGCTGCCCAGTTTGACTTATGCACCTTGTAT | 6A | 0 | 39.3463 | -0.0053 | -0.0273 | -0.0113 | -0.0127 | 0.0047 | 0.0176 | 0.0118 | 0.0224 | 0.0001 | 2.7255 | 1.90E-36 |  |  |  |  |
|  | rs30886 | TGCAGCGCGTCCCCGCCCTTTCCCGCCACGCGTCTACACCGAGATCGGAAGAGCGGGATCACCG | 6B | 2.273 | 6.2513 | -0.0015 | 0.0222 | -0.0007 | 0.0069 | 0.0025 | -0.0081 | -0.0002 | -0.0211 | 0 | 0.4058 | 6.67E-05 |  |  |  |  |
|  | rs30814 | TGCAGCGCGGGAGGACCCTCAAGACTGAGCTCTCCACCAACCAAAGCCTTTCATGGCCACCGCG | 7A | 71.904 | 9.4608 | 0.0007 | -0.0401 | 0 | -0.0611 | -0.0012 | 0.0563 | 0.0005 | 0.0449 | 0 | 0.618 | 9.01E-08 |  |  |  |  |
| Sphericity | rs30886 | TGCAGCGCGTCCCCGCCCTTTCCCGCCACGCGTCTACACCGAGATCGGAAGAGCGGGATCACCG | 6B | 2.273 | 9.2718 | 0.0103 | -0.1257 | 0.0107 | -0.0465 | -0.0195 | 0.0296 | -0.0016 | 0.1426 | 0.0005 | 0.6504 | 1.34E-07 |  |  |  |  |
|  | rs5644 | TGCAGAGATCCATTATATTACATCATCTGCAAGCTGCCCGAGATCGGAAGAGCGGGATCACCGA | 6B | 23.949 | 7.2866 | -0.0027 | -0.0866 | 0.0051 | -0.1201 | -0.0039 | 0.1391 | 0.0014 | 0.0676 | 0.0004 | 0.5092 | 8.20E-06 |  |  |  |  |
| Rectang | rs60571 | TGCAGTGCAGGGACGAGTACACGCTGGCATATATGTATTACATTCCGTTCACGGGGGCCAAGCC | 1B | 21.612 | 9.8683 | -0.0015 | -0.0053 | 0 | -0.0059 | 0.0007 | 0.0049 | 0.0008 | 0.0063 | 0 | 1.3827 | 3.82E-08 |  |  |  |  |
|  | rs48276 | TGCAGGCGGAGCAGTAGGCCATCCTCAATTCCATCCGCTCGGAGTCGGATGCGGAGGCCAGATG | 1B | 88.795 | 13.3025 | 0.001 | -0.0061 | 0.0022 | -0.0042 | -0.001 | 0.0051 | -0.0022 | 0.0053 | 0 | 1.8764 | 2.50E-11 |  |  |  |  |
|  | rs50993 | TGCAGGGGATCCAAGAGGCGAGGGAGATCGCGGCGGGTAAGGCTTTTCCCATGTAAAGTAAGTA | 3A | 171.063 | 7.7497 | -0.0004 | 0.0161 | -0.0011 | 0.0053 | 0.0009 | -0.0098 | 0.0006 | -0.0116 | 0 | 1.0814 | 3.17E-06 |  |  |  |  |
|  | rs12618 | TGCAGCAAGGTAGCCCAATCCTCTTTGTAGCAAAGGTCAGGCCGAGATCGGAAGAGCGGGATCA | 3B | 47.798 | 10.1767 | -0.0017 | 0.0017 | -0.0013 | 0.0046 | 0.0015 | -0.004 | 0.0015 | -0.0023 | 0 | 1.4267 | 1.99E-08 |  |  |  |  |
|  | rs1241 | TGCAGAACATGCGCTTGATAATGTAGCGTGTCGGCCGAGGATGTTACCCGCGCCGACCTGAATC | 3B | 76.224 | 5.3853 | 0.0016 | -0.0013 | 0 | -0.0034 | -0.0009 | 0.0026 | -0.0008 | 0.0021 | 0 | 0.748 | 0.000372 |  |  |  |  |
|  | rs49609 | TGCAGGGACCGTACGATGTCCATCCAACGGCTGTAGTGCTTCTTAACCCTCTGGTCTTCTTGTT | 4A | 61.015 | 18.4223 | -0.0029 |  | -0.0008 |  | 0.0007 |  | 0.003 |  | 0 | 2.6246 | 2.82E-18 |  |  |  |  |
|  | rs12060 | TGCAGCAAGACCCATAATGCTGCACAATCTCTTCTTCCCTATTTTCTACAACAAGACTCTTGTT | 7A | 0 | 12.3663 | -0.002 | -0.0001 | -0.0013 | -0.0009 | 0.001 | -0.0043 | 0.0022 | 0.0053 | 0 | 1.7411 | 1.87E-10 |  |  |  |  |
|  | rs6760 | TGCAGAGGCTCTCACTGCCGCCACGGGCTCGAGCAGCCTCCGCCGTGAGGTTCTCTCCATGTTG | 7A | 32.091 | 7.2548 | 0.0011 | -0.0086 | 0.0004 | -0.0012 | -0.0013 | 0.0039 | -0.0003 | 0.0059 | 0 | 1.0113 | 8.75E-06 |  |  |  |  |
| Area.1 | rs64886 | TGCAGTTTCGAAGTTATAGGACTAAAGGACACATCACCAATAGTTTTAGGACTTACGATGCATT | 1A | 44.512 | 7.0324 | 0.0727 | -2.7458 | 0.0945 | -1.1927 | -0.1173 | 2.2254 | -0.0498 | 1.713 | 0.0499 | 0.7287 | 1.38E-05 |  |  |  |  |
| ArBBox.1 | rs64886 | TGCAGTTTCGAAGTTATAGGACTAAAGGACACATCACCAATAGTTTTAGGACTTACGATGCATT | 1A | 44.512 | 6.795 | 0.1074 | -3.7796 | 0.0963 | -1.6259 | -0.1033 | 3.1521 | -0.1004 | 2.2533 | 0.0907 | 0.7432 | 2.23E-05 |  |  |  |  |
| Perim.2 | rs60836 | TGCAGTGCCTCTCTGAATTAAAAGAGGTAATAGCACCCCAGGTACCCTAACTTGCATAAGATGT | 1A | 54.745 | 5.5406 | -0.0524 | -0.0725 | -0.0784 | 0.0743 | 0.066 | -0.0733 | 0.0647 | 0.0715 | 0.0044 | 0.4358 | 0.000274 |  |  |  |  |
|  | rs40016 | TGCAGCTGGTCATAGGACACCTGGAGTCGGTCGAACGTCTCGACGGAGAGGACATTGAGCCCTG | 2A | 59.228 | 16.1318 | 0.1264 | -0.1695 | 0.097 | -0.1545 | -0.106 | 0.1078 | -0.1175 | 0.2162 | 0.0131 | 1.2954 | 5.38E-14 |  |  |  |  |
|  | rs45360 | TGCAGGATGAACAGGTAAGGTGGGAGGTGATCCAGGGGGTATGGGTCGAGATGCTATGCTATTC | 6B | 9.097 | 9.4423 | -0.0247 | 0.2515 | -0.0803 | 0.3021 | 0.0658 | -0.2139 | 0.0391 | -0.3397 | 0.0075 | 0.7484 | 9.36E-08 |  |  |  |  |
|  | rs57125 | TGCAGTAGTCCTGACGAGCAAATACCACGTCTCGTTCGCGCTCCTGTCGACAAACTTGAGGAAC | 6B | 92.187 | 5.5271 | 0.0761 | 0.1662 | 0.0271 | 0.1099 | -0.0125 | -0.0285 | -0.0907 | -0.2476 | 0.0044 | 0.4348 | 0.000281 |  |  |  |  |
| Area.2 | rs5425 | TGCAGAGAGAAAGAGGGGTTTCGGGCTACGCGGTTAACCGTTGGAGTACCAAACGACCTCCAAA | 2B | 59.184 | 9.471 | 0.1508 | 0.3801 | 0.0915 | 0.2985 | -0.0993 | -0.2598 | -0.1431 | -0.4188 | 0.0168 | 0.7484 | 8.82E-08 |  |  |  |  |
|  | rs20983 | TGCAGCCACACCCAGTCACCCACCGAAAACTCTGCCTCGCGGTGGTGGGCGTCGTAGTATTTCT | 4A | 62.152 | 8.3191 | 0.1839 | 0.2627 | 0.0271 | -0.0352 | -0.1096 | -0.07 | -0.1014 | -0.1575 | 0.0147 | 0.6559 | 9.77E-07 |  |  |  |  |
|  | rs41782 | TGCAGCTTTCAGGCATGCGTGCATGTCCTAAGAGTGTATGCCTCCTGTATTTTCCCTGTATGTG | 7B | 71.663 | 10.6678 | 0.0674 | -0.0306 | 0.1928 | -0.2099 | -0.1395 | 0.1227 | -0.1207 | 0.1179 | 0.0189 | 0.8449 | 7.04E-09 |  |  |  |  |
| ArBBox.2 | rs2102 | TGCAGAAGCTTGGGATACTTGGAGTATAGCAAAGGGCGGCATGTGTATAAAATTTAGTTTTGTA | 1A | 34.278 | 7.8588 | -0.0136 | -0.8642 | 0.002 | -1.0278 | 0.0454 | 1.2161 | -0.0337 | 0.6759 | 0.0228 | 0.6049 | 2.53E-06 |  |  |  |  |
|  | rs16497 | TGCAGCAGCAGCTGCTCGAGGACCTGCACGAACTTTGGGGTTGCCTCTTGTCTGAGACACGCCG | 6A | 99.391 | 17.2863 | -0.2745 | -0.0575 | -0.1593 | -0.1085 | 0.1279 | 0.1439 | 0.3059 | 0.0221 | 0.051 | 1.3552 | 4.31E-15 |  |  |  |  |
|  | rs41782 | TGCAGCTTTCAGGCATGCGTGCATGTCCTAAGAGTGTATGCCTCCTGTATTTTCCCTGTATGTG | 7B | 71.663 | 10.9297 | 0.1578 | 0.1401 | 0.1964 | -0.2051 | -0.1989 | -0.1597 | -0.1552 | 0.2247 | 0.0319 | 0.8463 | 4.03E-09 |  |  |  |  |
| Volume | rs56585 | TGCAGTACTTGCGTCAAACCGCCTCCATCTTGCACTGCATCAGGGCCTCCGTCTCTGCTGGCGG | 1A | 44.512 | 12.1739 | 0.8498 | 1.8736 | 0.6657 | 1.4703 | -0.781 | -2.5976 | -0.7344 | -0.7463 | 0.6234 | 0.987 | 2.83E-10 |  |  |  |  |
|  | rs38918 | TGCAGCTGCCTGCTCATCCTCACCATCTCAATCTGTGTACGAGTAGATCCCATGGTTTACCAGT | 2A | 11.39 | 24.5753 | -1.1715 | -2.5804 | -0.9312 | -1.6618 | 1.1749 | 1.6433 | 0.9279 | 2.5989 | 1.2893 | 2.0414 | 4.42E-22 |  |  |  |  |
|  | rs38828 | TGCAGCTGCCGAGGTACGCTTCGACTTGAAGTTCGAGAATGTGCCCTTGGTGATCCTCTACATG | 4A | 147.563 | 4.9265 | 0.3302 | 0.1767 | 0.6503 | -0.016 | -0.5655 | 0.1612 | -0.415 | -0.3218 | 0.2487 | 0.3938 | 0.000909 |  |  |  |  |
|  | rs62825 | TGCAGTTCAAAGGAGTTCAATGGAAAGGGCGGGTCGGGGCTTATAAACAGGTCCTGCCGCTCCT | 7A | 0.5685 | 7.9072 | 0.6892 | 0.1288 | 0.5959 | 0.2859 | -0.6126 | -0.4137 | -0.6725 | -0.001 | 0.4015 | 0.6358 | 2.29E-06 |  |  |  |  |
|  | rs41782 | TGCAGCTTTCAGGCATGCGTGCATGTCCTAAGAGTGTATGCCTCCTGTATTTTCCCTGTATGTG | 7B | 71.663 | 7.238 | 0.2701 | -0.4453 | 0.819 | -1.5678 | -0.587 | 0.7576 | -0.5021 | 1.2555 | 0.3671 | 0.5812 | 9.05E-06 |  |  |  |  |
|  | rs29065 | TGCAGCGATATCTCCCTACAACAATCAGACGGCTCCACGACGGGATGGCGGCTCCTCCATGAGG | 6A | 25.146 | 11.9358 | -0.1777 | -3.2922 | -0.0918 | -3.3345 | 0.0989 | 3.5286 | 0.1706 | 3.0981 | 0.4988 | 0.624 | 4.71E-10 |  |  |  |  |
|  | rs31607 | TGCAGCGGCAAGAACATGGATTAGTCCTTCCAGGAGACAGAAACCACACGGATCGATCGACCCG | 6A | 99.391 | 15.5325 | -0.8601 | -0.0068 | -0.8259 | 0.0254 | 0.8562 | -0.0187 | 0.8299 | 0.0001 | 0.6537 | 0.8178 | 1.99E-13 |  |  |  |  |
|  | rs56117 | TGCAGTACAATTTTCCCACGGCCACACCAGCCAACCAGGATACAACACAGGTAACGCAAGGAAA | 7A | 63.946 | 14.2069 | -0.577 | 3.2412 | -0.5815 | 3.0985 | 0.5794 | -3.2171 | 0.5791 | -3.1226 | 0.5963 | 0.746 | 3.54E-12 |  |  |  |  |

**Supplementary Table 4**. A summary of identified TFs among the different sets of DEGs in wheat.

| Transcription factors | | | | | |
| --- | --- | --- | --- | --- | --- |
| AP2/ERF-ERF(38) | ELI (1) | MYB (15) | Trihelix (4) | GeBP (1) | OFP (2) |
| AP2/ERF-RAV (10) | FAR1 (1) | MYB-related (23) | WRKY (28) | GRAS (5) | PLATZ (1) |
| B3 (6) | GRAS (6) | NAC (28) | AP2/ERF-ERF(19) | HB-other (2) | RWP-RK (1) |
| bHLH (21) | HB-HD-ZIP (8) | NF-YC (4) | B3 (11) | HB-HD-ZIP (5) | TCP Tify (2) |
| bZIP (12) | HB-other (7) | NF-X1 (1) | bHLH (12) | HSF | Trihelix (2) |
| C2C2-CO-Dof (1) | HB-WOX (1) | NF-YB (1) | bZIP (3) | MADS-MIKC (1) | TUB (2) |
| C2C2-Dof (5) | HSF (15) | NF-YA (9) | C2C2-GATA (1) | MADS-M-type (6) | Whirly (3) |
| C2C2-GATA (1) | LOB (2) | OFP (1) | C2C2-CO-like(1) | MYB (6) | WRKY zf-HD (1) |
| C2H2 (16) | MADS-MIKC (2) | RWP-RK (2) | C2H2 (6) CPP (4) | MYB-related (17) |  |
| C3H (1) | MADS-M-type (14) | SBP TCP (4) | GARP-G2-like (5) | NAC NF-YC (2) |  |

**Supplementary Table 5.** Overview on the landraces and cultivars of Iranian wheat studied

| Genetic background: Landraces | | | | | | |
| --- | --- | --- | --- | --- | --- | --- |
| No. | Region of origin (Province) | USDA_PI_NO |  | No. | Region of origin (Province) | USDA_PI_NO |
| 1 | Gilan | 625281 |  | 105 | Tehran | 621669 |
| 2 | Mazandaran | 625362 |  | 106 | Gazvin | 621704 |
| 3 | Khorasan | 625433 |  | 107 | Gazvin | 621706 |
| 4 | Khorasan | 625661 |  | 108 | Gazvin | 621712 |
| 5 | Khorasan | 625810 |  | 109 | Gazvin | 621716 |
| 6 | Kerman | 626156 |  | 110 | Azarbayjan-Gharbi | 620903 |
| 7 | Kerman | 626158 |  | 111 | Hamadan | 621420 |
| 8 | Kerman | 626215 |  | 112 | Hamadan | 621421 |
| 9 | Sistan-Balouchestan | 626223 |  | 113 | Bakhtaran | 621492 |
| 10 | Sistan-Balouchestan | 626226 |  | 114 | Hamadan | 621565 |
| 11 | Sistan-Balouchestan | 626234 |  | 115 | Mazandaran | 622084 |
| 12 | Markazi | 625080 |  | 116 | Gilan | 622098 |
| 13 | Markazi | 625081 |  | 117 | Gilan | 622099 |
| 14 | Markazi | 625123 |  | 118 | Gilan | 622105 |
| 15 | Markazi | 625127 |  | 119 | Mazandaran | 622247 |
| 16 | Markazi | 625139 |  | 120 | Mazandaran | 622264 |
| 17 | Mazandaran | 625263 |  | 121 | Mazandaran | 622272 |
| 18 | Sistan-Balouchestan | 626260 |  | 122 | Khorasan | 622311 |
| 19 | Sistan-Balouchestan | 626261 |  | 123 | Gazvin | 621717 |
| 20 | Esfahan | 626358 |  | 124 | Gazvin | 621735 |
| 21 | Esfahan | 626360 |  | 125 | Gazvin | 621736 |
| 22 | Esfahan | 626565 |  | 126 | Markazi | 621869 |
| 23 | Esfahan | 626566 |  | 127 | Markazi | 621908 |
| 24 | Esfahan | 626573 |  | 128 | Zanjan | 622063 |
| 25 | Ilam | 626699 |  | 129 | Yazd | 623109 |
| 26 | Hamadan | 626706 |  | 130 | Fars | 623123 |
| 27 | Khorasan | 626736 |  | 131 | Fars | 623125 |
| 28 | Yazd | 626747 |  | 132 | Fars | 623127 |
| 29 | Yazd | 626764 |  | 133 | Esfahan | 623008 |
| 30 | Khorasan | 626776 |  | 134 | Esfahan | 623069 |
| 31 | Esfahan | 626814 |  | 135 | Bakhtaran | 623090 |
| 32 | Esfahan | 626825 |  | 136 | Khorasan | 623091 |
| 33 | Yazd | 626846 |  | 137 | Khorasan | 622379 |
| 34 | Markazi | 626855 |  | 138 | Esfahan | 622894 |
| 35 | Fars | 626872 |  | 139 | Azarbayjan-Gharbi | 623266 |
| 36 | Kerman | 626908 |  | 140 | Bakhtaran | 623274 |
| 37 | Gilan | 626923 |  | 141 | Hamadan | 623291 |
| 38 | Gilan | 626924 |  | 142 | Yazd | 623318 |
| 39 | Hormozgan | 626932 |  | 143 | Fars | 623338 |
| 40 | Azarbayjan-Shargi | 626881 |  | 144 | Bakhtaran | 623344 |
| 41 | Fars | 626883 |  | 145 | Kordestan | 623345 |
| 42 | Azarbayjan-Shargi | 626895 |  | 146 | Kerman | 623377 |
| 43 | Azarbayjan-Shargi | 626904 |  | 147 | Kerman | 623379 |
| 44 | Zanjan | 627072 |  | 148 | Azarbayjan-Gharbi | 623136 |
| 45 | Khouzestan | 627099 |  | 149 | Fars | 623139 |
| 46 | Zanjan | 627102 |  | 150 | Azarbayjan-Gharbi | 623161 |
| 47 | Mazandaran | 627103 |  | 151 | Azarbayjan-Gharbi | 623162 |
| 48 | Khorasan | 627189 |  | 152 | Gilan | 623169 |
| 49 | Zanjan | 627055 |  | 153 | Khorasan | 623176 |
| 50 | Gilan | 627057 |  | 154 | Azarbayjan-Gharbi | 623510 |
| 51 | Markazi | 627061 |  | 155 | Bakhtaran | 623905 |
| 52 | Kerman | 627066 |  | 156 | Bakhtaran | 623908 |
| 53 | Hormozgan | 626933 |  | 157 | Bakhtaran | 623909 |
| 54 | Kerman | 626943 |  | 158 | Bakhtaran | 623953 |
| 55 | Azarbayjan-Gharbi | 626958 |  | 159 | Hamadan | 623980 |
| 56 | Esfahan | 626978 |  | 160 | Hamadan | 624215 |
| 57 | Khouzestan | 627036 |  | 161 | Ilam | 624240 |
| 58 | Khouzestan | 627038 |  | 162 | Ilam | 624251 |
| 59 | Azarbayjan-Gharbi | 627043 |  | 163 | Ilam | 623475 |
| 60 | Gilan | 627054 |  | 164 | Ilam | 623503 |
| 61 | Khorasan | 627236 |  | 165 | Bakhtaran | 623506 |
| 62 | Yazd | 627299 |  | 166 | Bakhtaran | 623507 |
| 63 | Hormozgan | 627356 |  | 167 | Bakhtaran | 623508 |
| 64 | Markazi | 627359 |  | 168 | Kerman | 623382 |
| 65 | Kerman | 627360 |  | 169 | Sistan-Balouchestan | 623417 |
| 66 | Bakhtaran | 627385 |  | 170 | Azarbayjan-Shargi | 623421 |
| 67 | Zanjan | 627399 |  | 171 | Azarbayjan-Shargi | 623428 |
| 68 | Azarbayjan-Shargi | 627410 |  | 172 | Ilam | 623473 |
| 69 | Bakhtaran | 627414 |  | 173 | Hamadan | 624596 |
| 70 | Bakhtaran | 627416 |  | 174 | Bakhtaran | 624804 |
| 71 | Bakhtaran | 627417 |  | 175 | Bakhtaran | 624805 |
| 72 | Hamadan | 627423 |  | 176 | Ilam | 624818 |
| 73 | Khorasan | 627460 |  | 177 | Ilam | 624837 |
| 74 | Yazd | 627484 |  | 178 | Ilam | 624838 |
| 75 | Azarbayjan-Shargi | 627787 |  | 179 | Ilam | 624846 |
| 76 | Kerman | 627842 |  | 180 | Ilam | 624849 |
| 77 | Sistan-Balouchestan | 627845 |  | 181 | Ilam | 624861 |
| 78 | Sistan-Balouchestan | 627849 |  | 182 | Kordestan | 624315 |
| 79 | Sistan-Balouchestan | 627852 |  | 183 | Bakhtaran | 624378 |
| 80 | Sistan-Balouchestan | 627853 |  | 184 | Bakhtaran | 624381 |
| 81 | Mazandaran | 627856 |  | 185 | Hamadan | 624576 |
| 82 | Zanjan | 627873 |  | 186 | Hamadan | 624580 |
| 83 | Esfahan | 627688 |  | 187 | Hamadan | 624582 |
| 84 | Yazd | 627723 |  | 188 | Hamadan | 624585 |
| 85 | Azarbayjan-Shargi | 627760 |  | 189 | Tehran | 624944 |
| 86 | Azarbayjan-Shargi | 627551 |  | 190 | Tehran | 624946 |
| 87 | Kordestan | 627587 |  | 191 | Tehran | 624947 |
| 88 | Esfahan | 627616 |  | 192 | Tehran | 624956 |
| 89 | Azarbayjan-Shargi | 627881 |  | 193 | Tehran | 624963 |
| 90 | Azarbayjan-Shargi | 627883 |  | 194 | Gazvin | 624980 |
| 91 | Mazandaran | 627905 |  | 195 | Gazvin | 624983 |
| 92 | Markazi | 627908 |  | 196 | Gazvin | 624985 |
| 93 | Markazi | 627948 |  | 197 | Gazvin | 624990 |
| 94 | Hamadan | 627963 |  | 198 | Markazi | 625047 |
| 95 | Zanjan | 627987 |  | 199 | Ilam | 624863 |
| 96 | Bakhtaran | 627990 |  | 200 | Ilam | 624864 |
| 97 | Bakhtaran | 628012 |  | 201 | Kordestan | 624894 |
| 98 | Mazandaran | 628084 |  | 202 | Kordestan | 624900 |
| 99 | Markazi | 628088 |  | 203 | Kordestan | 624901 |
| 100 | Esfahan | 628114 |  | 204 | Hamadan | 624910 |
| 101 | Ilam | 628189 |  | 205 | Hamadan | 624911 |
| 102 | Kordestan | 621619 |  | 206 | Hamadan | 624925 |
| 103 | Tehran | 621650 |  | 207 | Tehran | 624939 |
| 104 | Tehran | 621668 |  | 208 | Tehran | 624941 |

| Genetic background: Cultivars | | | |
| --- | --- | --- | --- |
| No. | Variety Name | Introduced year | Growth Habit |
| 209 | 4820 | 1951 | Spring |
| 210 | ADL | 1976 | Spring |
| 211 | AFLAK | 2010 | Spring |
| 212 | AKBARI | 2006 | Spring |
| 213 | ALBORZ | 1978 | Spring |
| 214 | ARTA | 2006 | Spring |
| 215 | AZADI | 1979 | Facultative |
| 216 | AZAR | 1957 | Winter |
| 217 | BAYAT | 1976 | Spring |
| 218 | BISTON | 1980 | Spring |
| 219 | CHAMRAN | 1997 | Spring |
| 220 | CHAMRAN-2 | 2013 | Spring |
| 221 | DARAB 1 | 1980 | Spring |
| 222 | DARAB 2 | 1995 | Spring |
| 223 | DASTJERDI | 1960 | Spring |
| 224 | DAYHIM | 1968 | Spring |
| 225 | DN-11 | --- | --- |
| 226 | FALAT | 1990 | Spring |
| 227 | FONG | --- | --- |
| 228 | FONTANA | --- | --- |
| 229 | GAHAR | 1996 | Spring |
| 230 | GHODS | 1988 | Spring |
| 231 | GOLESTAN | 1986 | Spring |
| 232 | HOMA | 2009 | Winter |
| 233 | KARAJ 1 | 1974 | Facultative |
| 234 | KARAJ 2 | 1974 | Winter |
| 235 | KARAJ 3 | 1974 | Winter |
| 236 | KARIM | 2011 | Spring |
| 237 | Gascogne | 1994 | --- |
| 238 | KAVEH | 1980 | Spring |
| 239 | KHAZAR 1 | 1974 | Spring |
| 240 | KOOHDASHT | 2002 | Spring |
| 241 | MAHDAVI | 1995 | Spring |
| 242 | MAROON | 1991 | Spring |
| 243 | MIHAN | 2010 | Winter |
| 244 | MOGHAN 1 | 1974 | Spring |
| 245 | MOGHAN 2 | 1974 | Spring |
| 246 | MOGHAN 3 | 2006 | Spring |
| 247 | MORVARID | 2009 | Spring |
| 248 | MV-17 | 1993 | Winter |
| 249 | NAVID 1990 | 1990 | Facultative |
| 250 | NAZ | 1978 | Spring |
| 251 | NEISHABOUR | 2006 | Spring |
| 252 | NICKNEJAD | 1995 | Spring |
| 253 | OFOGH | 2012 | Spring |
| 254 | OHADI | 2010 | Winter |
| 255 | PANJAMO 62 | 1968 | Spring |
| 256 | PISHGAM | 2008 | Facultative |
| 257 | QABOOS | 2014 | Spring |
| 258 | RAYHANI | 1942 | Spring |
| 259 | RIJAW | 2011 | Facultative |
| 260 | SIOSSON | 1994 | Spring |
| 261 | SHAHI | 1967 | Winter |
| 262 | SHAHPASSAND | 1942 | Winter |
| 263 | SIRVAN | 2012 | Spring |
| 264 | SISTAN | 2006 | Spring |
| 265 | TAK-AB | 2013 | Spring |
| 266 | TOUS | 2002 | Facultative |
| 267 | TOBARI 66 | 1969 | Spring |
| 268 | UROUM | 2009 | Winter |
| 269 | VEE/NAC | 1997 | Spring |
| 270 | ZARE | 2010 | Facultative |
| 271 | ZARRIN | 1995 | Spring |
| 272 | SHANGHAI #7 | --- | Spring |
| 273 | INIA 66 | 1969 | Spring |
| 274 | ARVAND 1 | 1974 | Spring |
| 275 | ROSHAN | 1960 | Spring |
| 276 | RASHID | 1968 | Facultative |
| 277 | SABALAN | 1980 | Spring |
| 278 | DARYA | 2006 | Spring |
| 279 | ATRAK | 1995 | Spring |
| 280 | BAHAR | 2007 | Spring |
| 281 | SEPAHAN | 2006 | Spring |
| 282 | BAM | 2006 | Spring |
| 283 | SHIRAZ | 2002 | Spring |
| 284 | PISHTAZ | 2002 | Spring |
| 285 | HAMOON | 2002 | Spring |
| 286 | DEZ | 2002 | Spring |
| 287 | SHIROODI | 1997 | Spring |
| 288 | MARVDASHT | 1999 | Spring |
| 289 | ZAGROS | 1996 | Spring |
| 290 | TAJAN | 1995 | Spring |
| 291 | ALVAND | 1995 | Facultative |
| 292 | KAVIR | 1997 | Spring |
| 293 | PARSI | 2009 | Spring |
| 294 | SIVAND | 2009 | Spring |
| 295 | BEZOSTAYA | 1969 | Winter |
| 296 | AKOVA | 1958 | Winter |
| 297 | SHAHRYAR | 2002 | Winter |
| 298 | AZAR 2 | 1997 | Winter |

**A B**


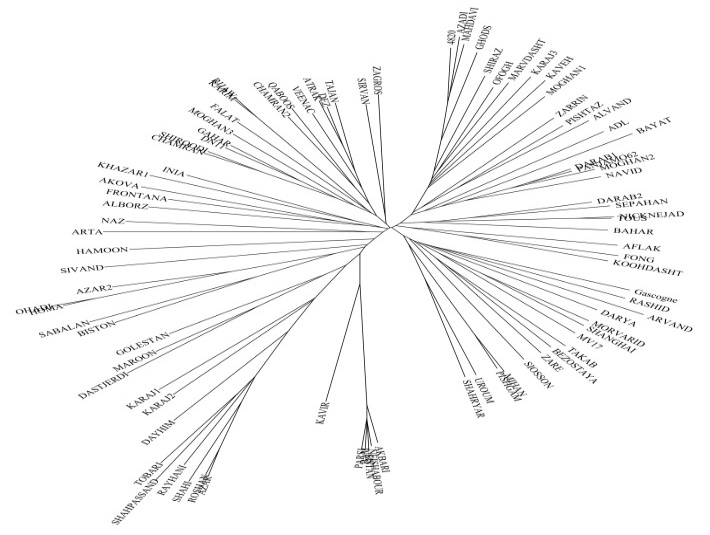

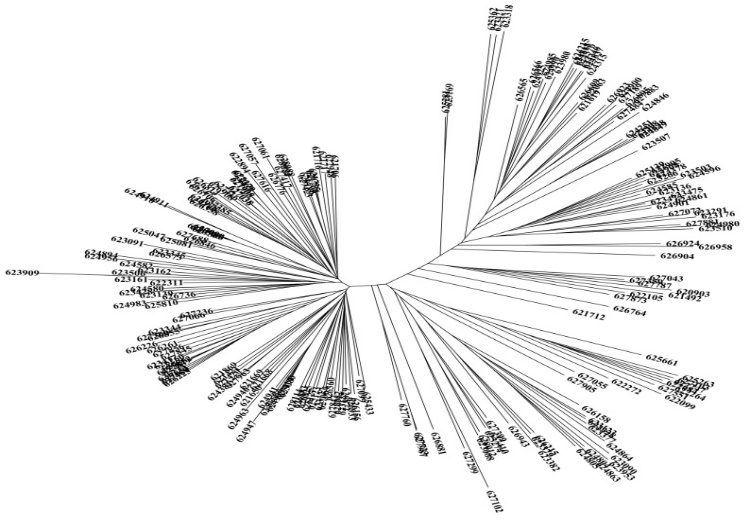


**Supplementary Fig. 1** The dendrogram of Neighbor-Joining clustering was constructed using 43,525 SNPs for 90 Iranian hexaploid wheat cultivars (A) and 208 wheat landraces (B) collected from different zones.

| MaxR |  |
| --- | --- |
| 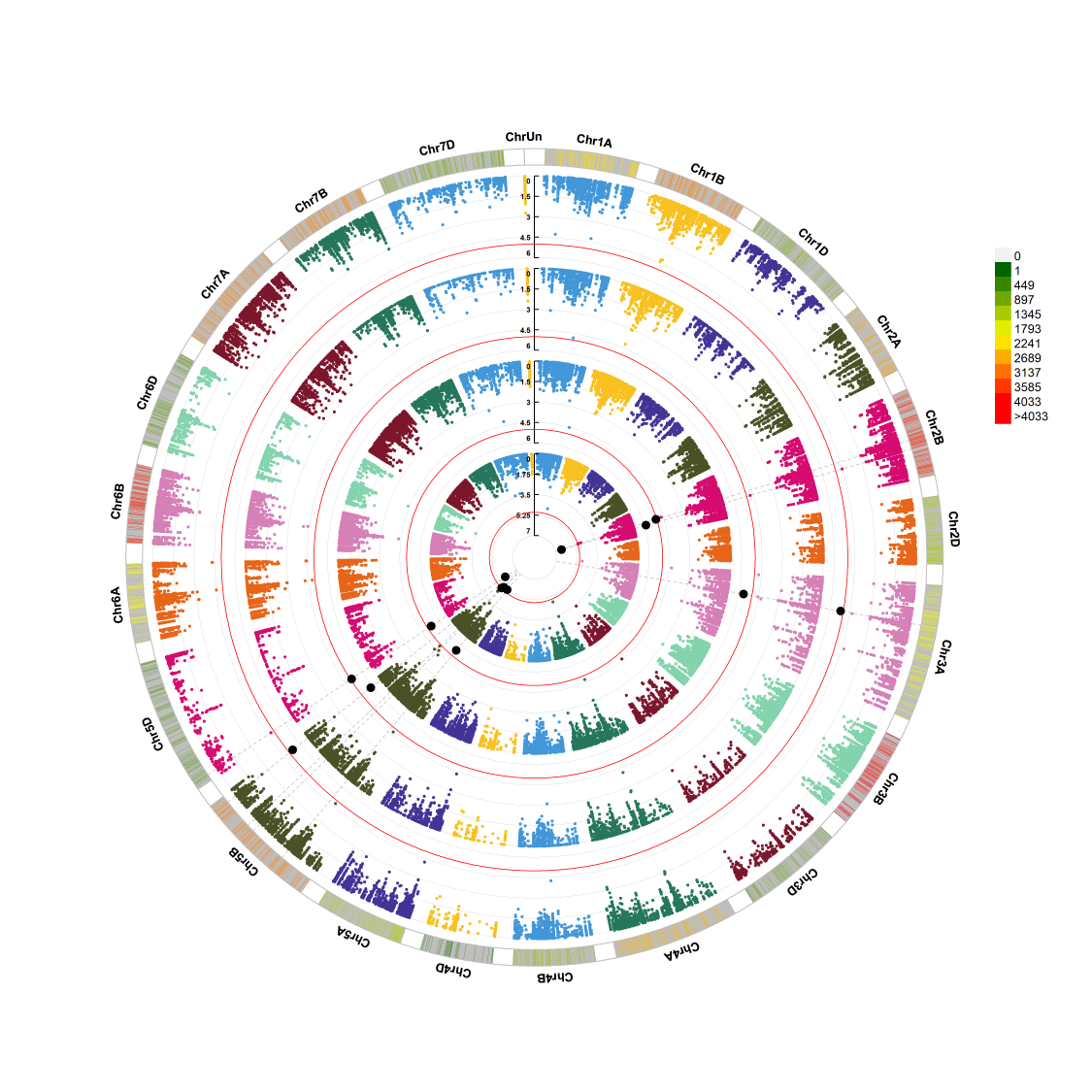 | mrMLM well-watered MLM well-watered   \| 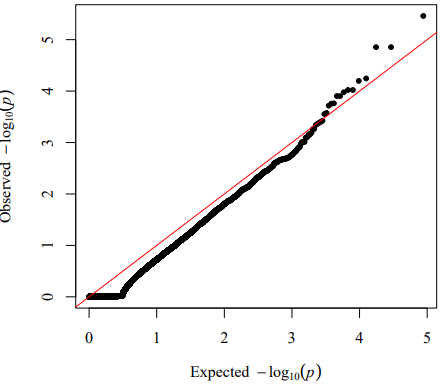 \| 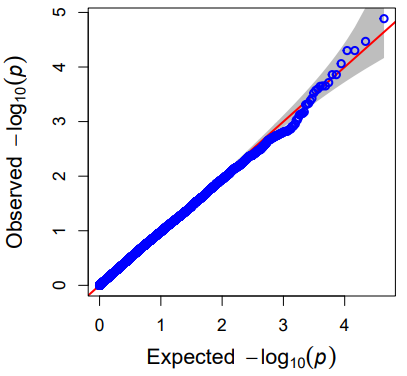 \| \| --- \| --- \| \| mrMLM rain-fed \| MLM rain-fed \| \| 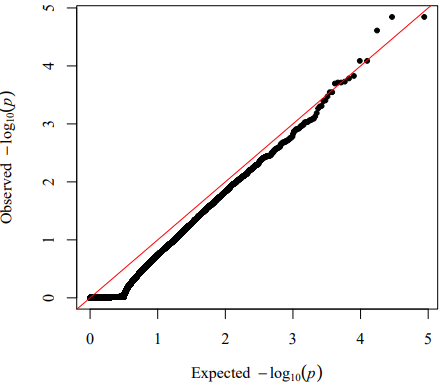 \| 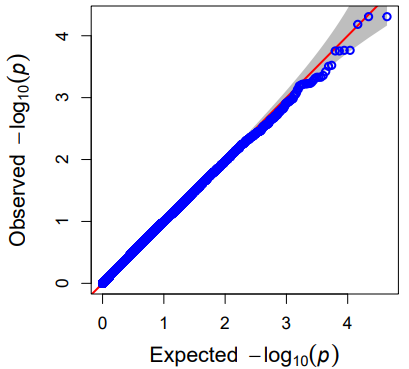 \| |
| MinR |  |
| 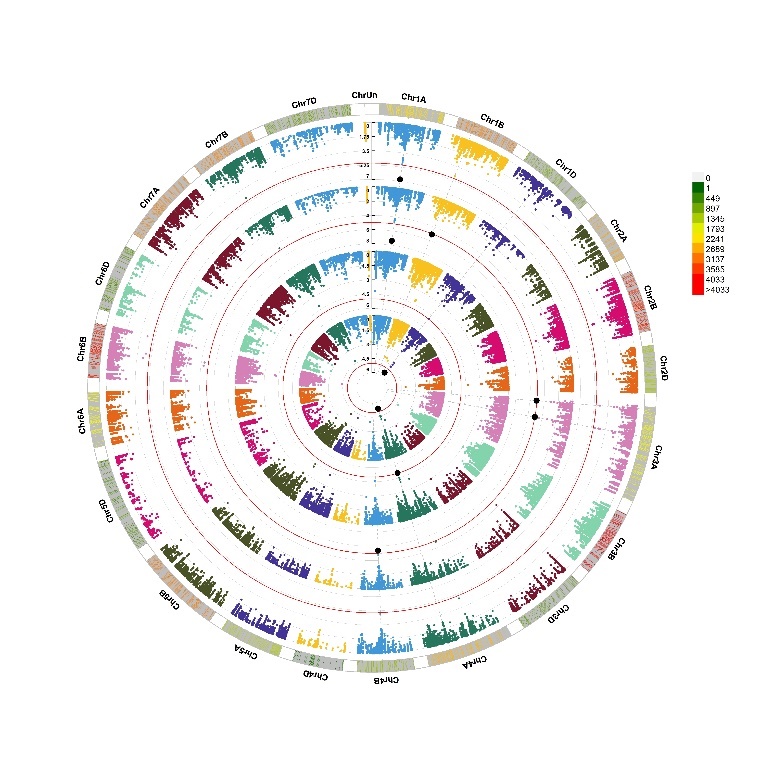 | mrMLM well-watered MLM well-watered   \| 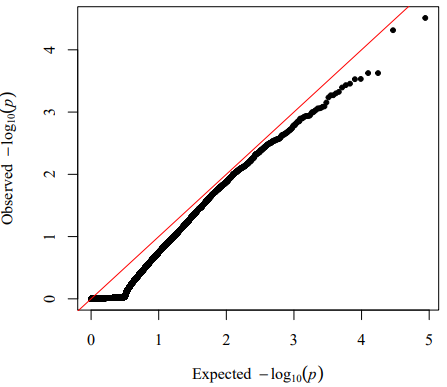 \| 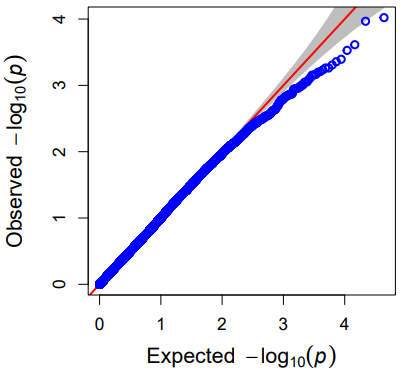 \| \| --- \| --- \| \| mrMLM rain-fed \| MLM rain-fed \| \| 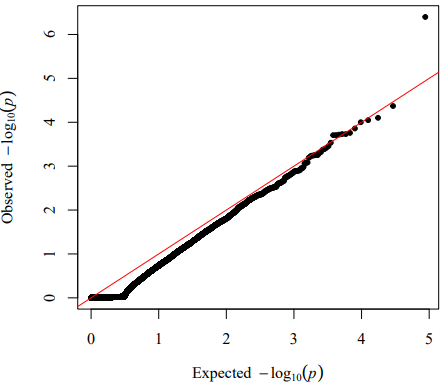 \| 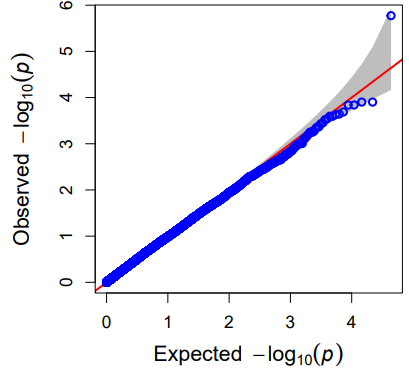 \| |
| MBCRadius |  |
| 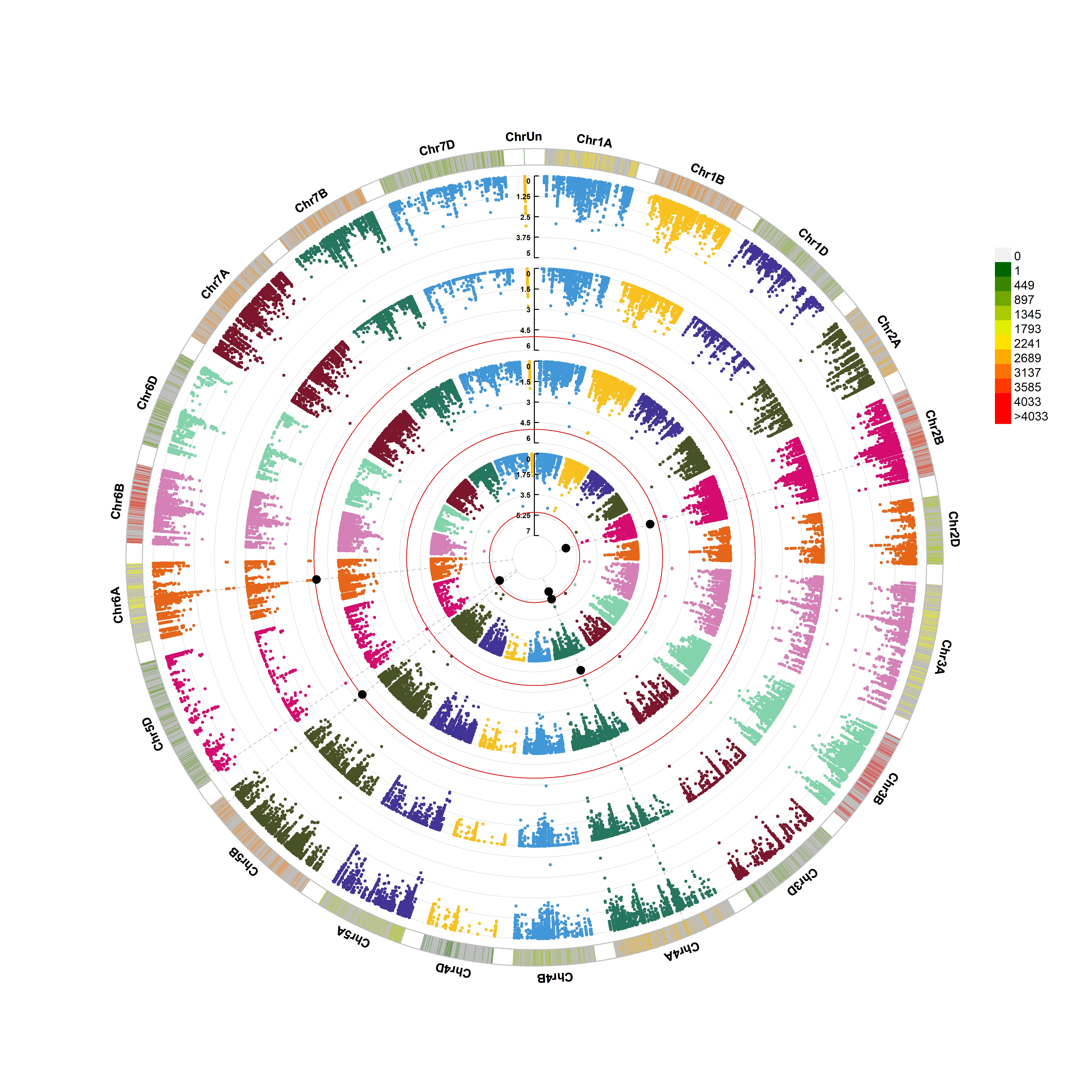 | mrMLM well-watered MLM well-watered   \| 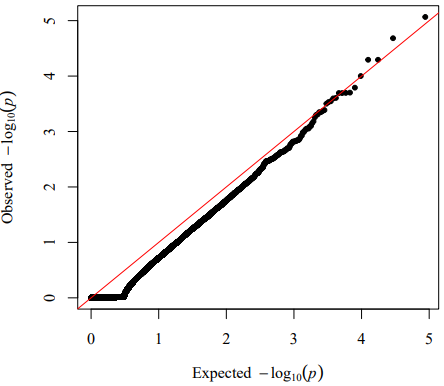  mrMLM rain-fed \| 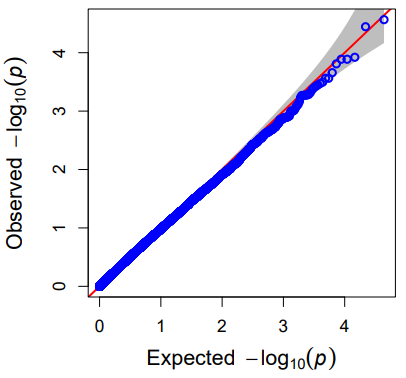  MLM rain-fed \| \| --- \| --- \| \| 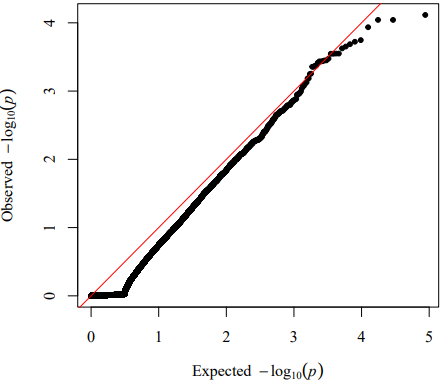 \| 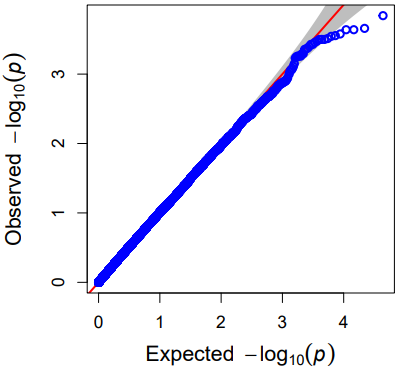 \| |
| CHull |  |
| 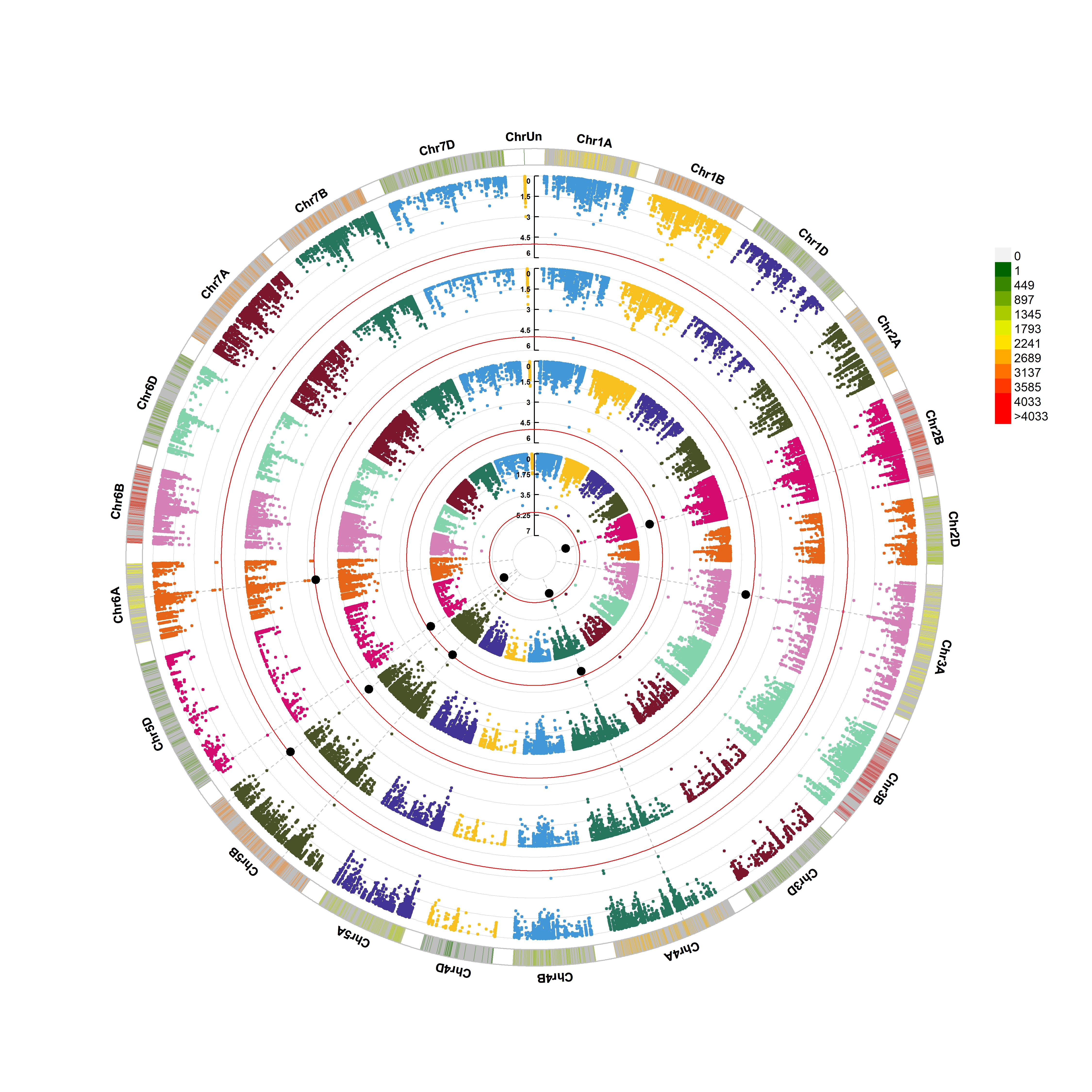 | mrMLM well-watered MLM well-watered   \| 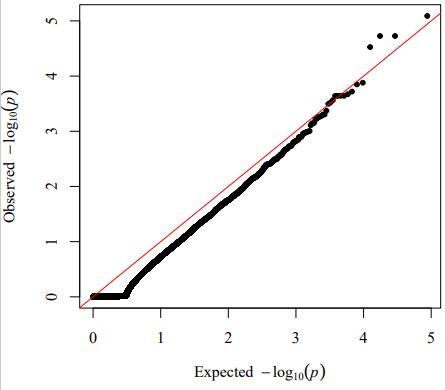  mrMLM rain-fed \| 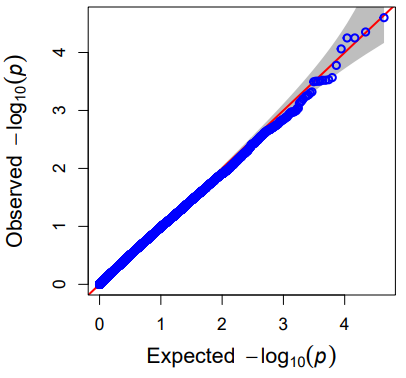  MLM rain-fed \| \| --- \| --- \| \| 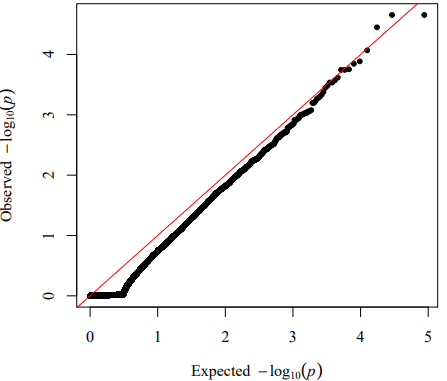 \| 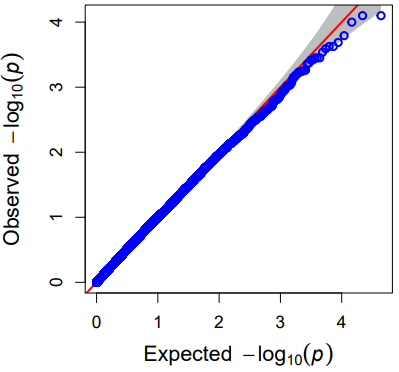 \| |
| CArea |  |
| 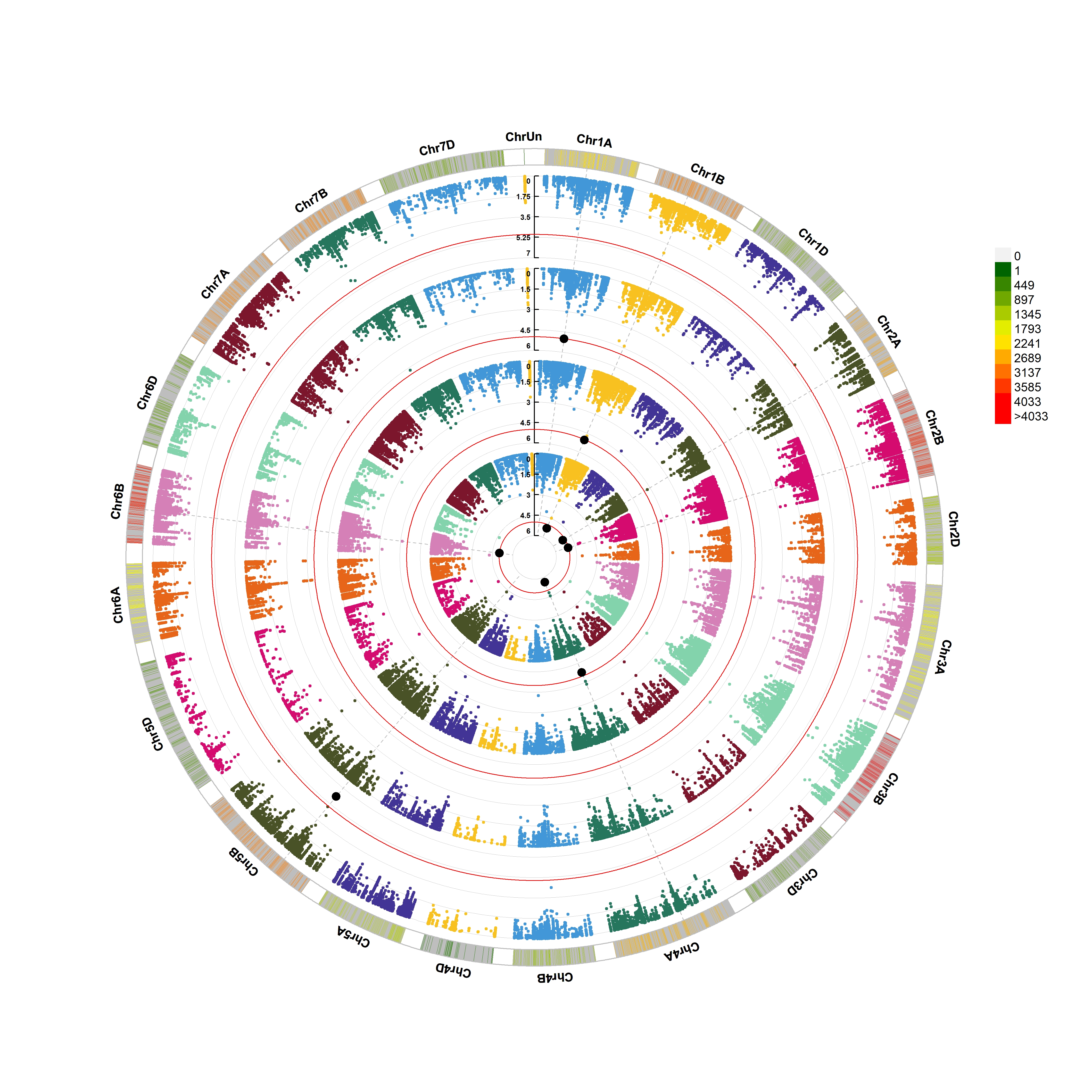 | mrMLM well-watered MLM well-watered   \| 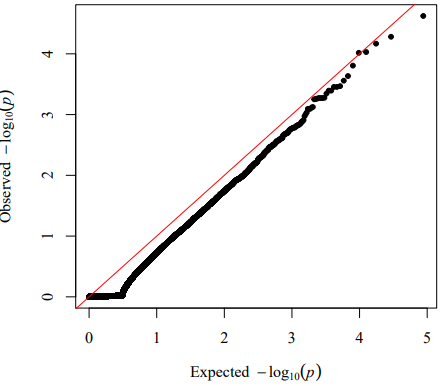 \| 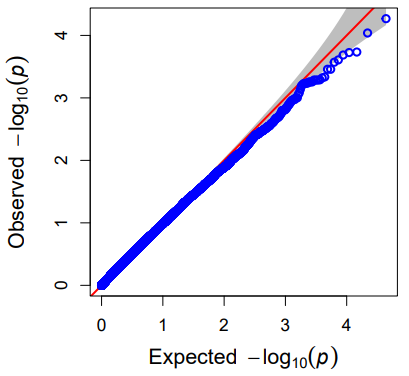 \| \| --- \| --- \| \| mrMLM rain-fed \| MLM rain-fed \| \| 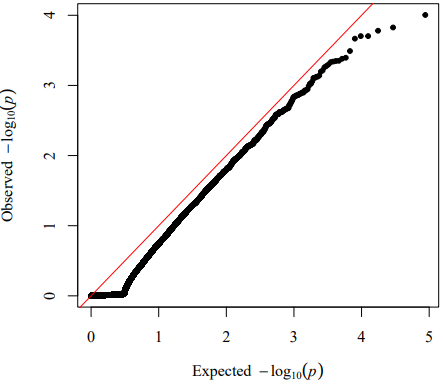 \| 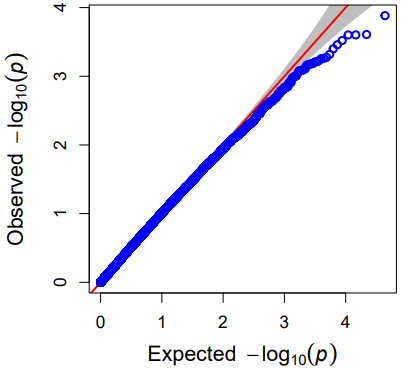 \| |
| Area |  |
| 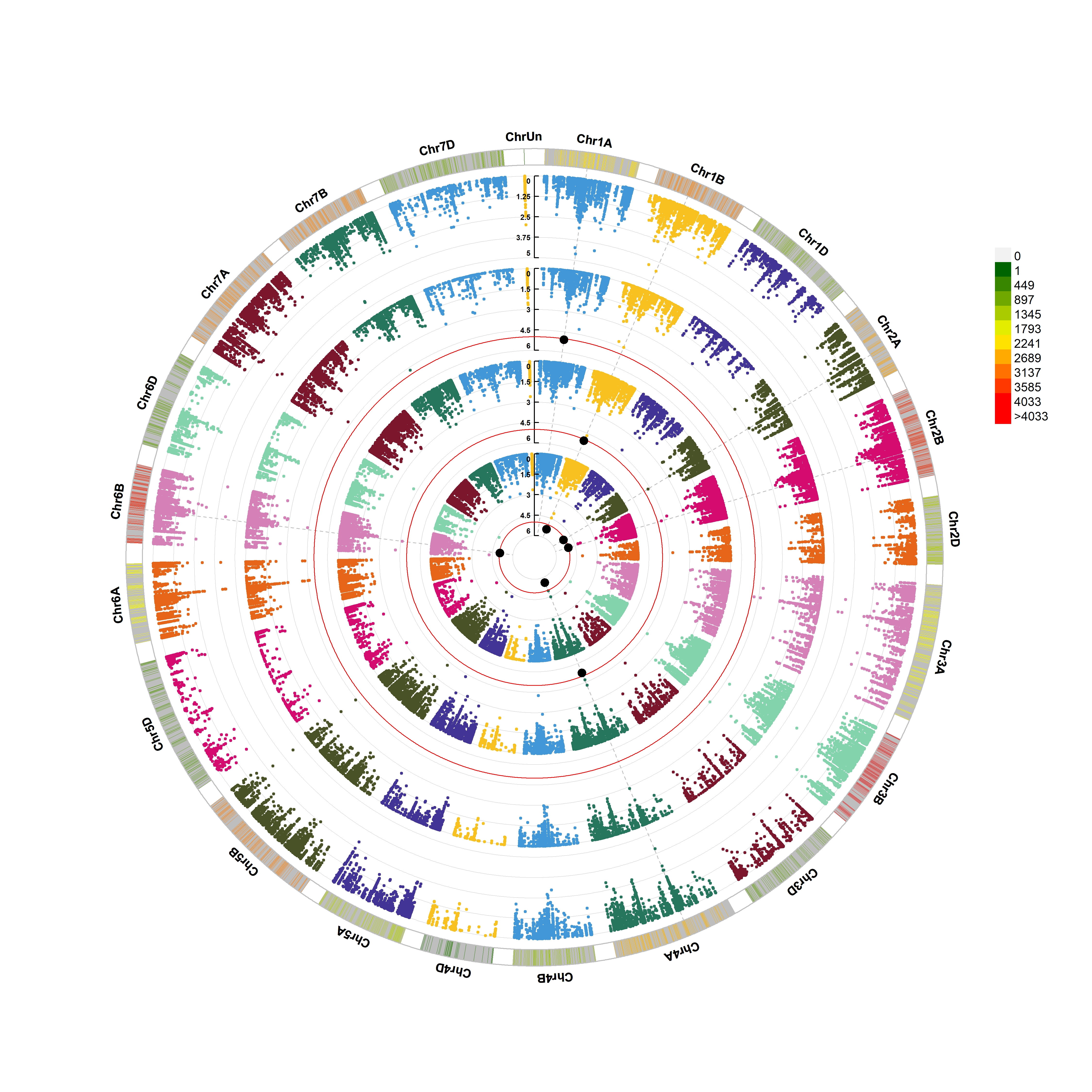 | mrMLM well-watered MLM well-watered   \| 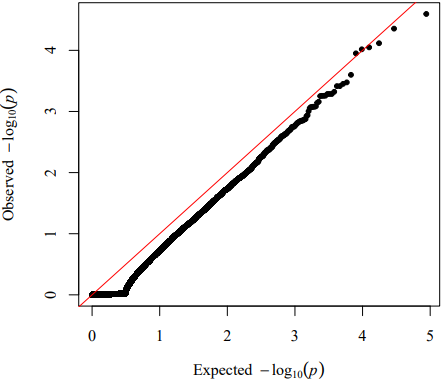  mrMLM rain-fed \| 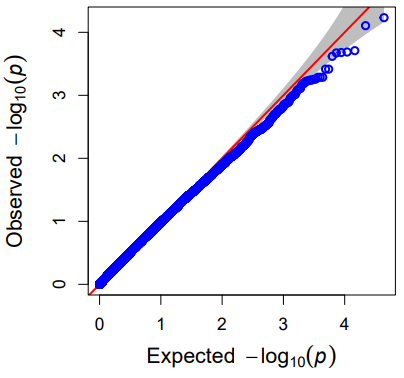  MLM rain-fed \| \| --- \| --- \| \| 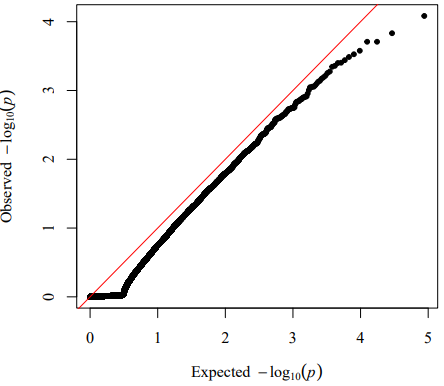 \| 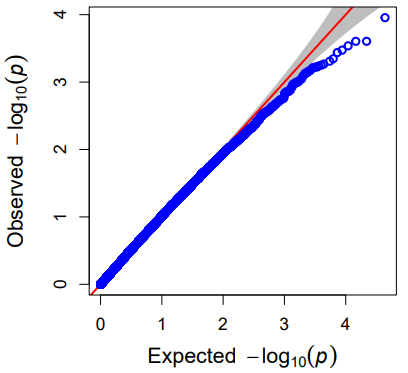 \| |
| Perim |  |
| 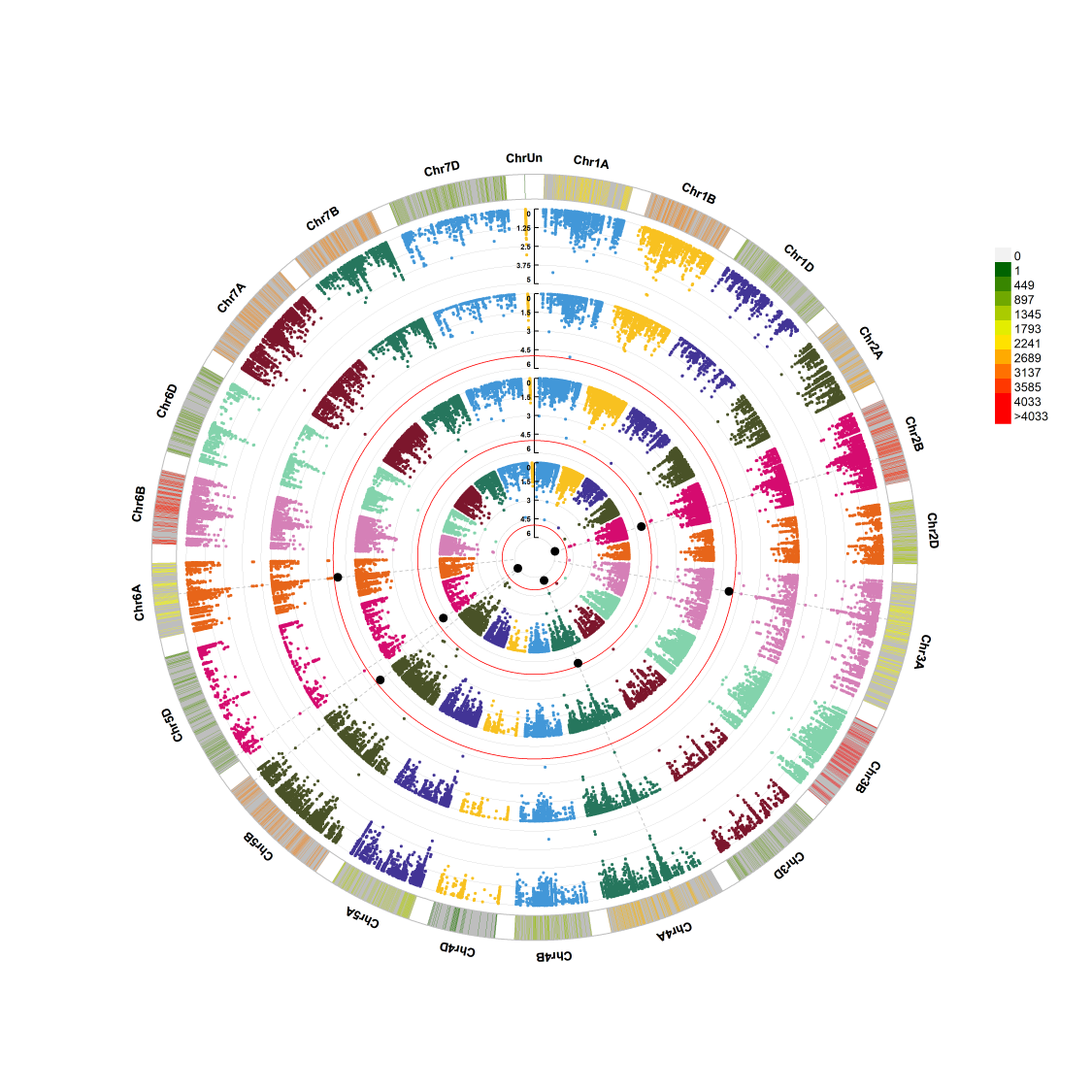 | mrMLM well-watered MLM well-watered   \| 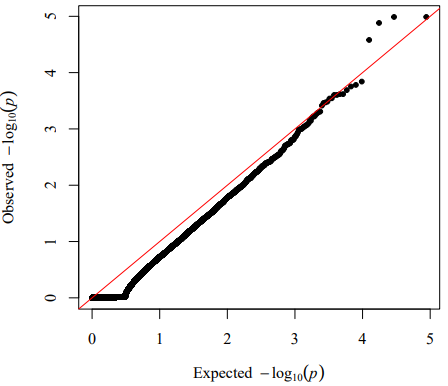 \| 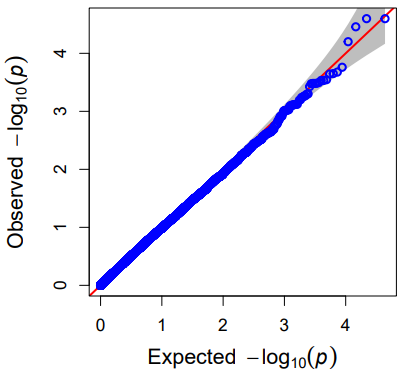 \| \| --- \| --- \| \| mrMLM rain-fed \| MLM rain-fed \| \| 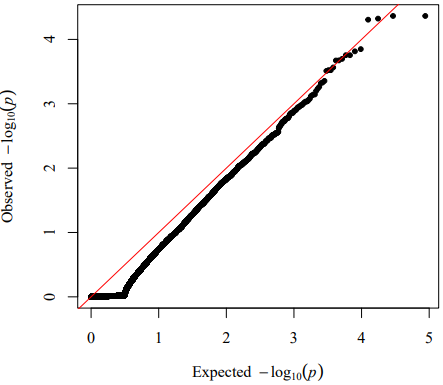 \| 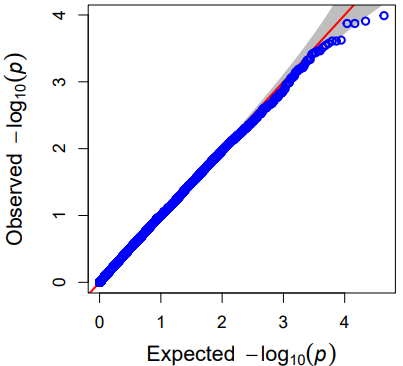 \| |
| Frete |  |
| 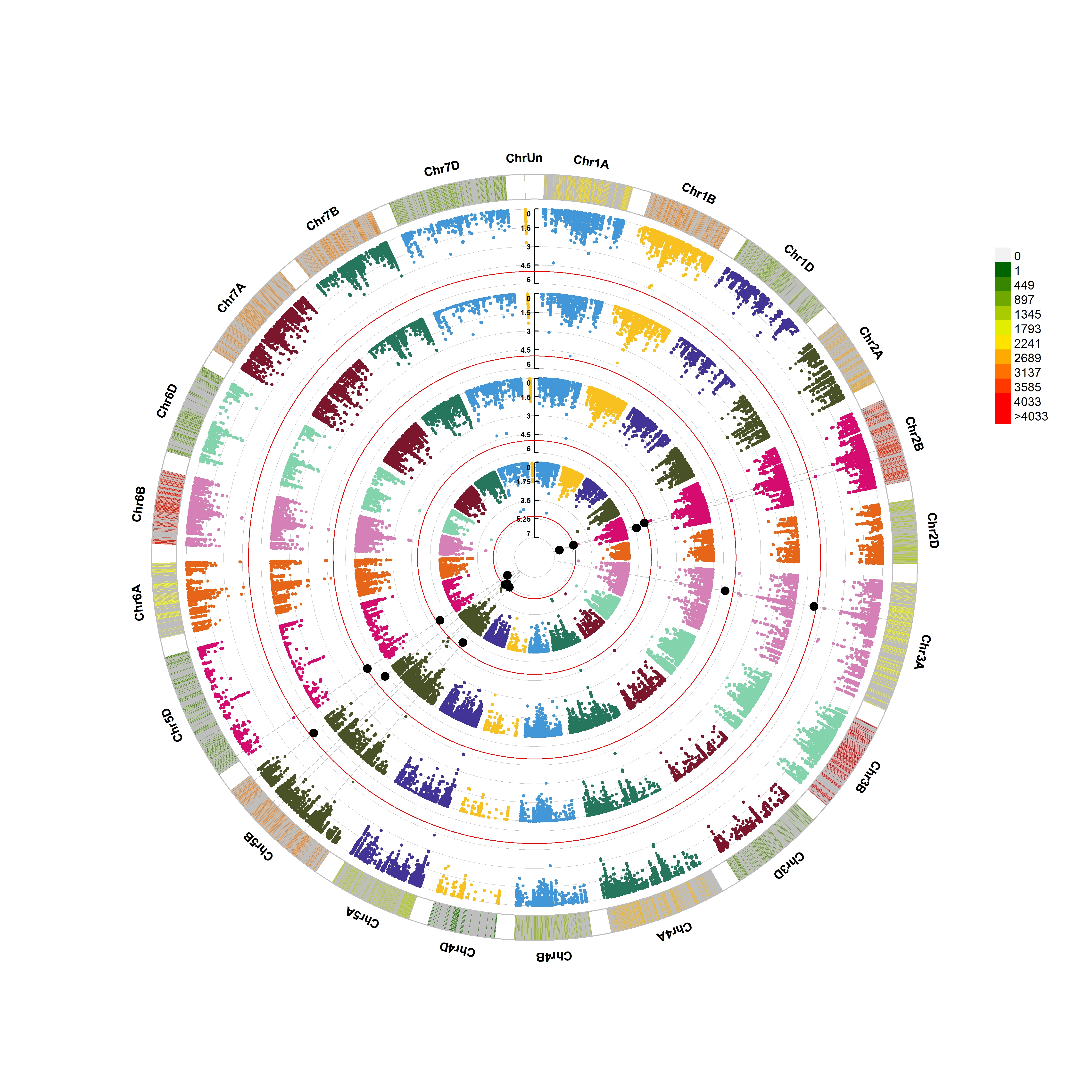 | mrMLM well-watered MLM well-watered   \| 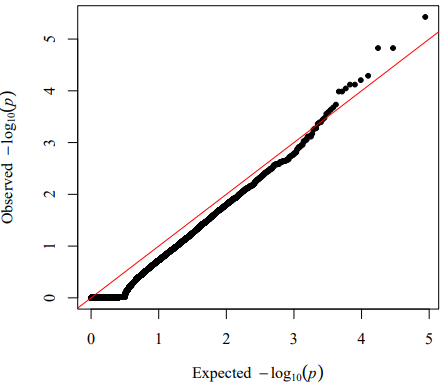  mrMLM rain-fed \| 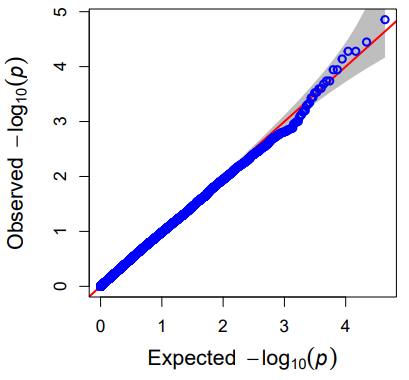 \| \| --- \| --- \| \|  \| MLM rain-fed \| \| 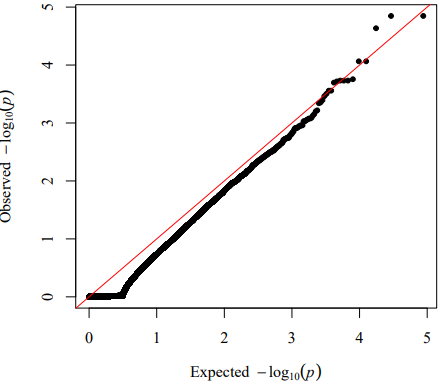 \| 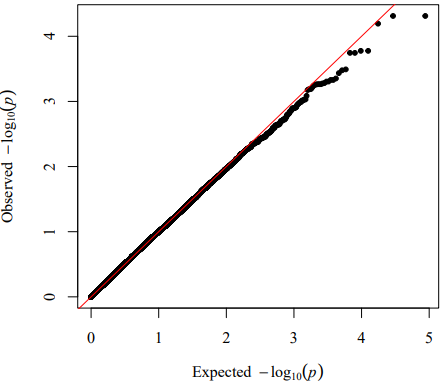 \| |
| Breadth |  |
| 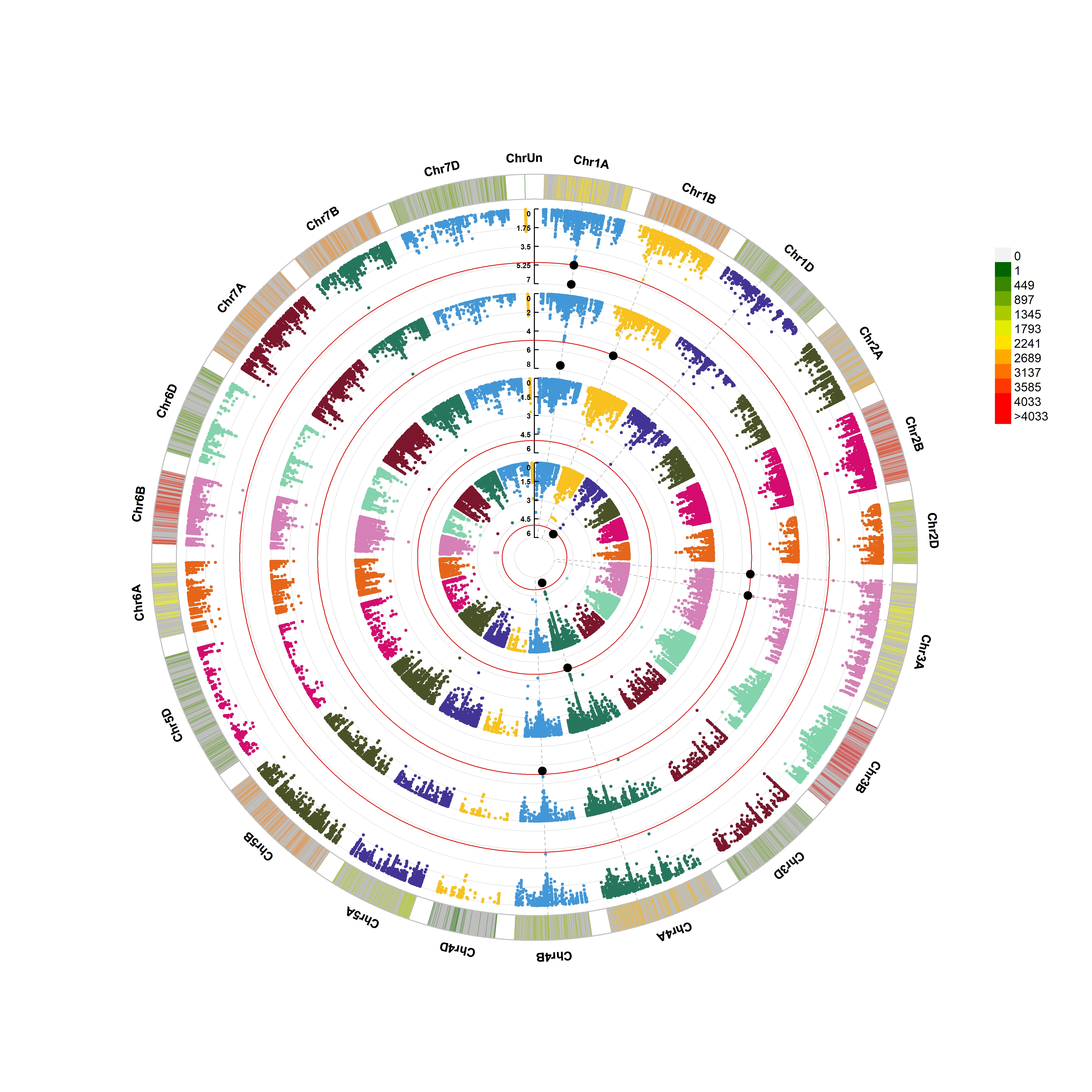 | mrMLM well-watered MLM well-watered   \| 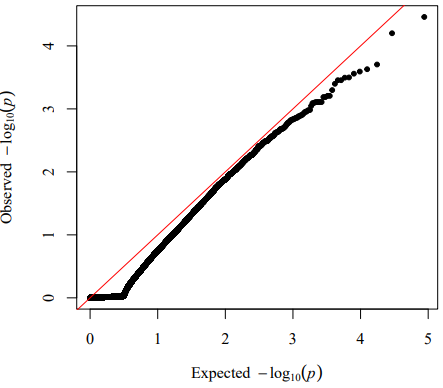  mrMLM rain-fed \| 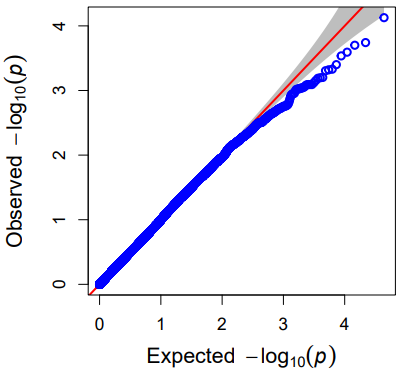 \| \| --- \| --- \| \|  \| MLM rain-fed \| \| 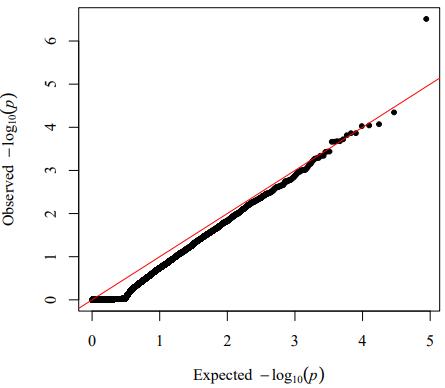 \| 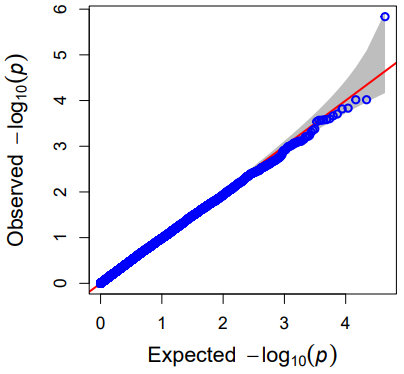 \| |
| ArBBox |  |
| 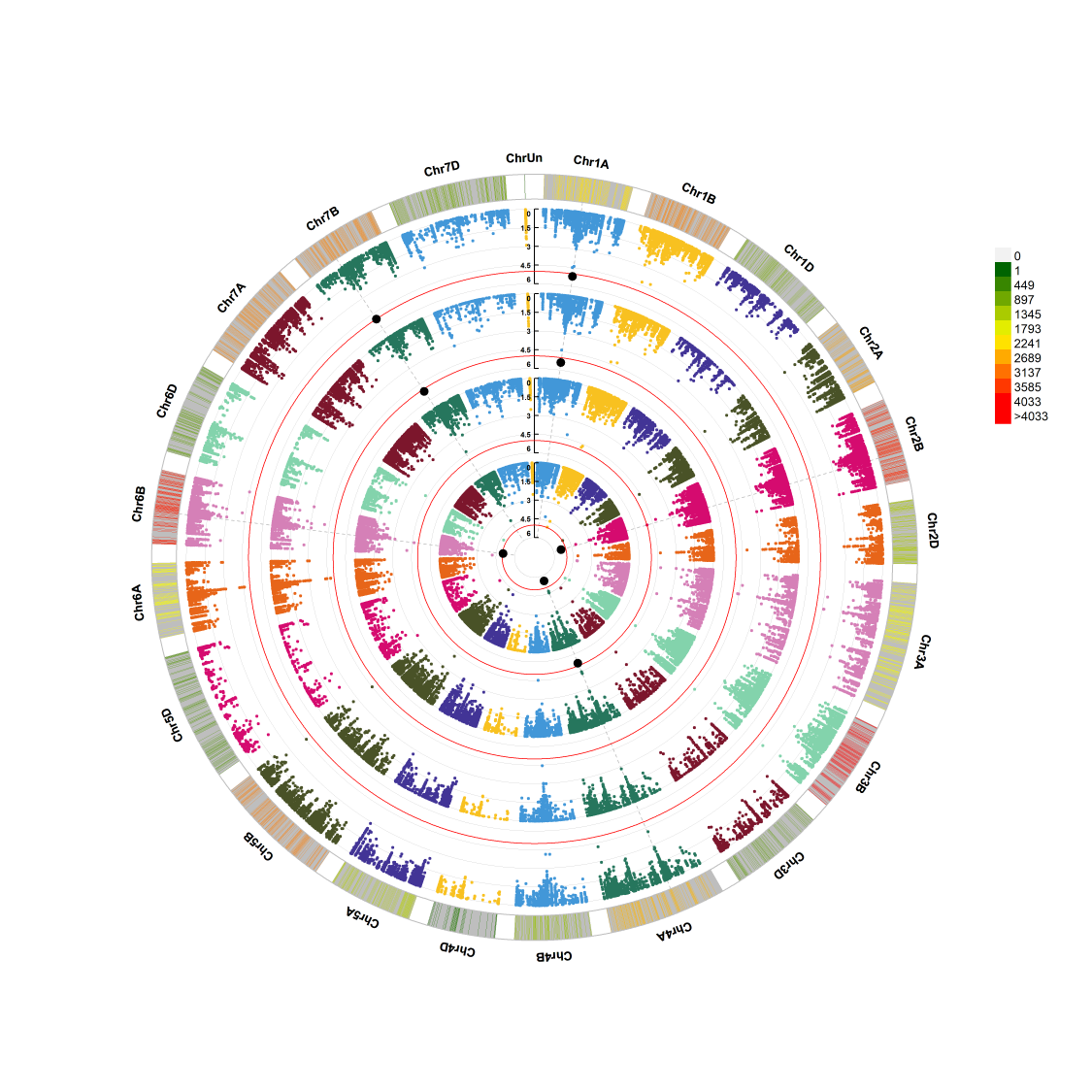 | mrMLM well-watered MLM well-watered   \| 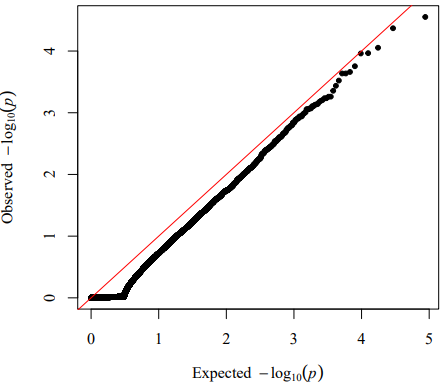 \| 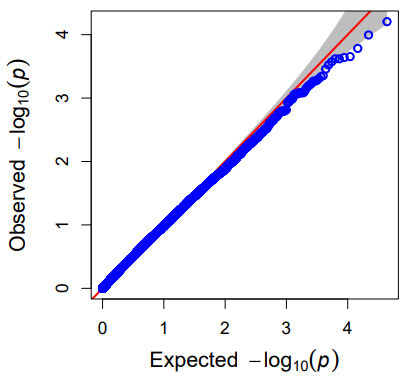 \| \| --- \| --- \| \| mrMLM rain-fed \| MLM rain-fed \| \| 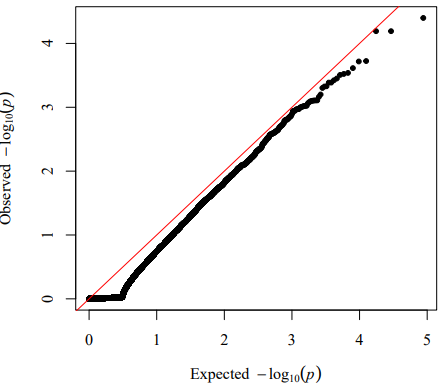 \| 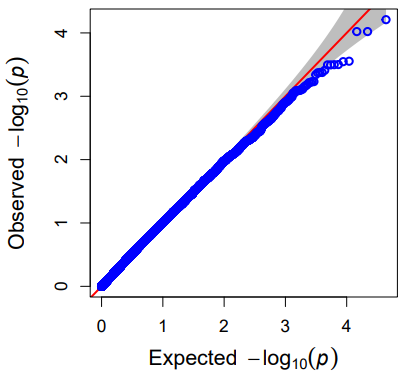 \| |
| Aspect ratio |  |
| 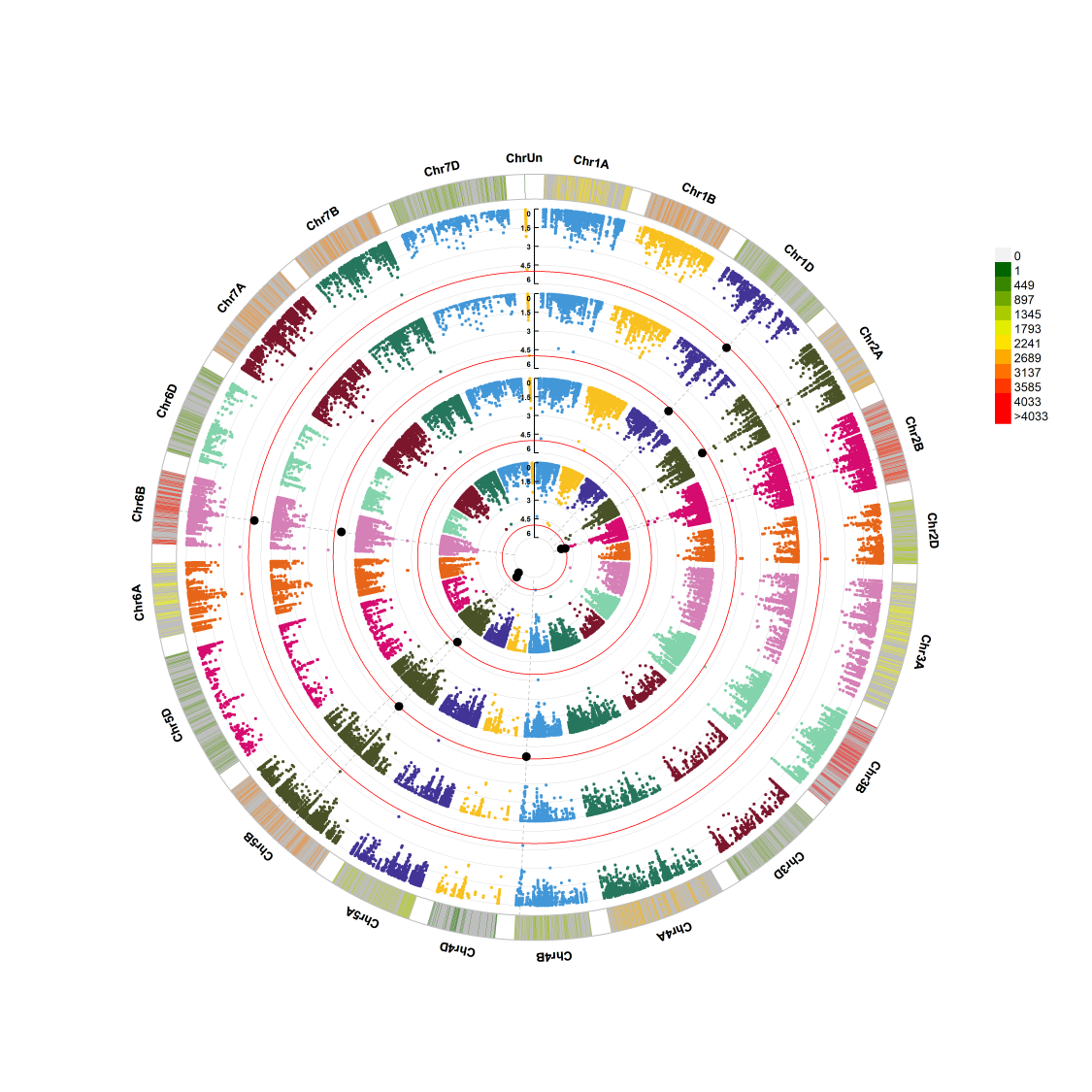 | mrMLM well-watered MLM well-watered   \| 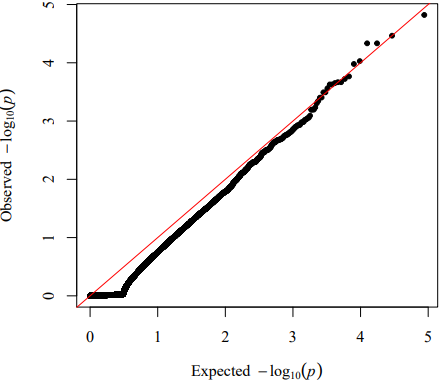 \| 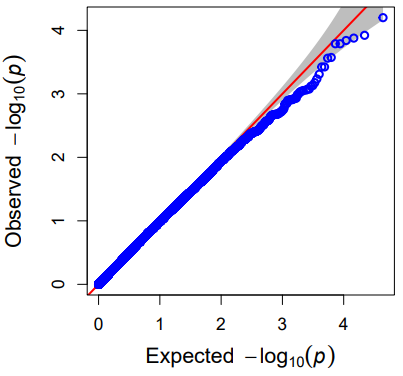 \| \| --- \| --- \| \| mrMLM rain-fed \| MLM rain-fed \| \| 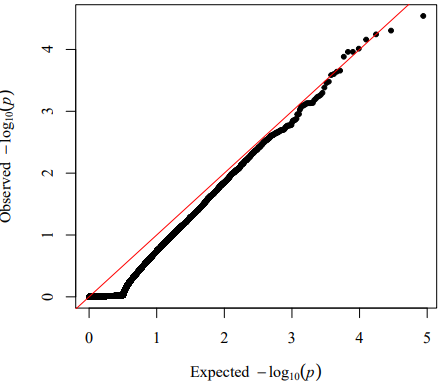 \| 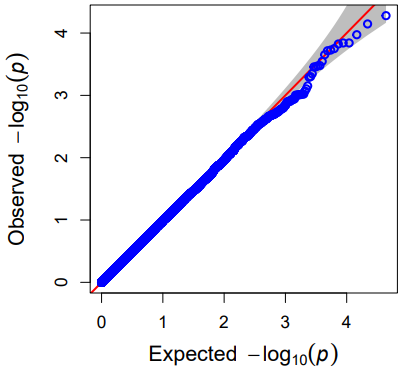 \| |
| Circ |  |
| 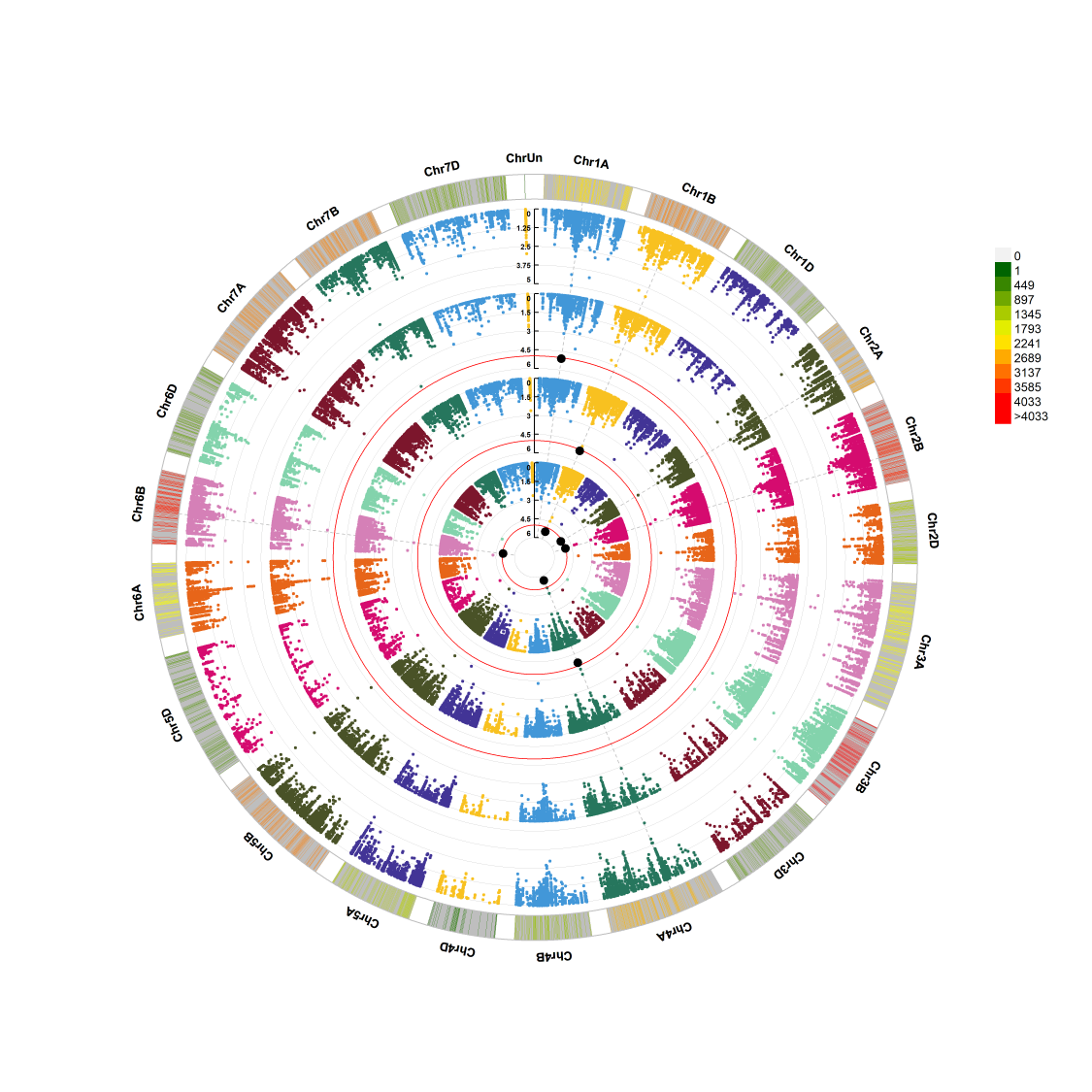 | mrMLM well-watered MLM well-watered   \| 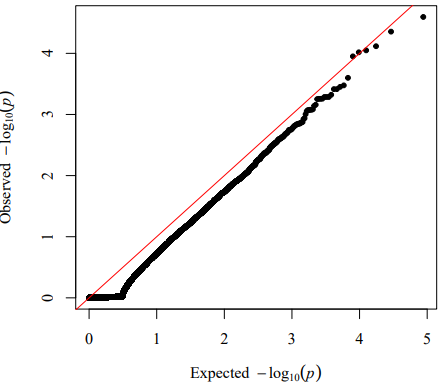 \| 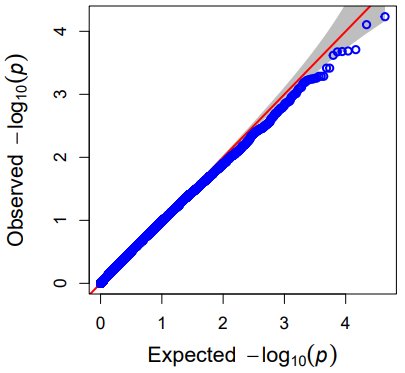 \| \| --- \| --- \| \| mrMLM rain-fed \| MLM rain-fed \| \| 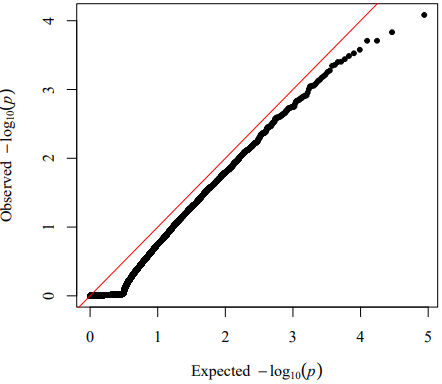 \| 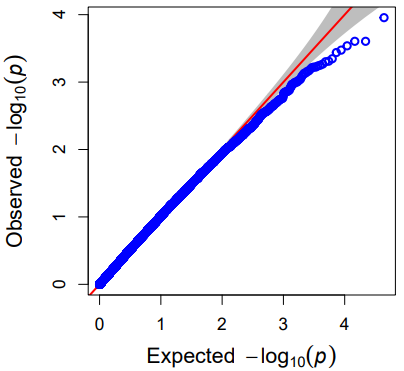 \| |
| Roundness |  |
| 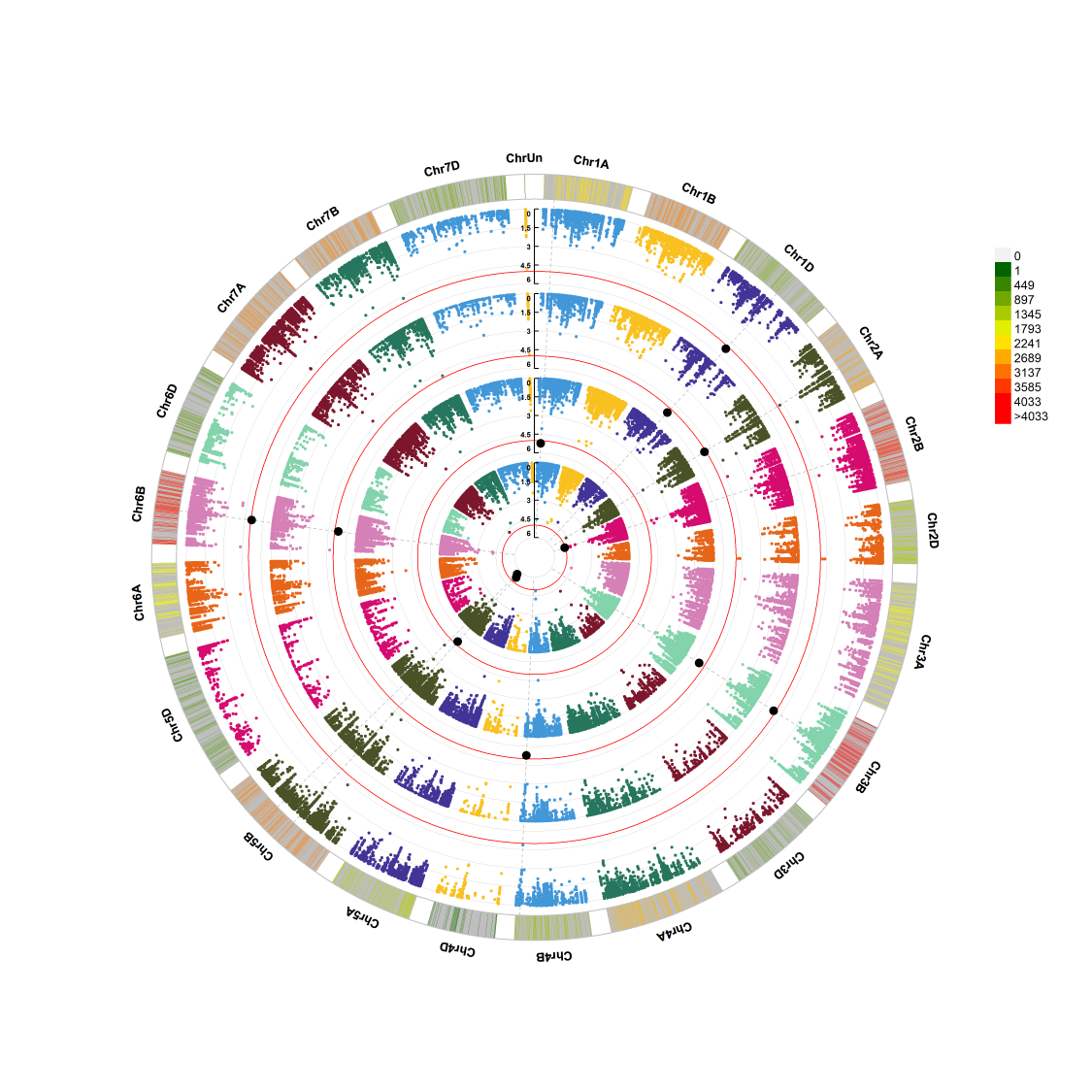 | mrMLM well-watered MLM well-watered   \| 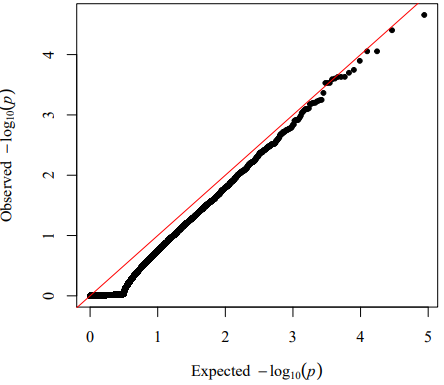 \| 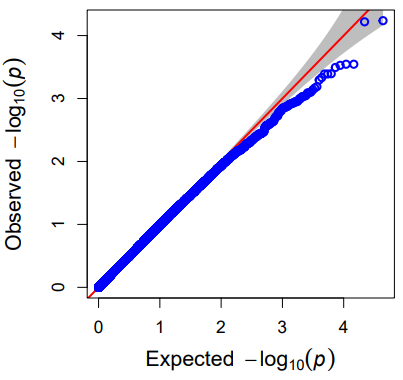 \| \| --- \| --- \| \| mrMLM rain-fed \| MLM rain-fed \| \| 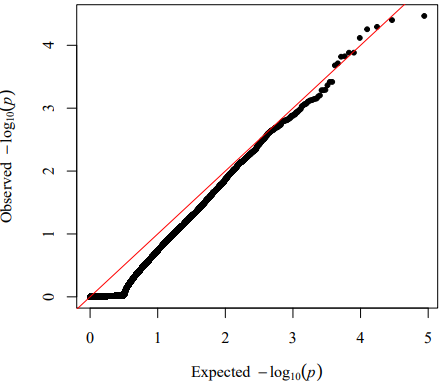 \| 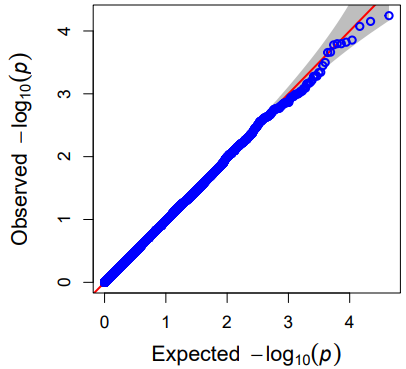 \| |
| ArEquivD |  |
| 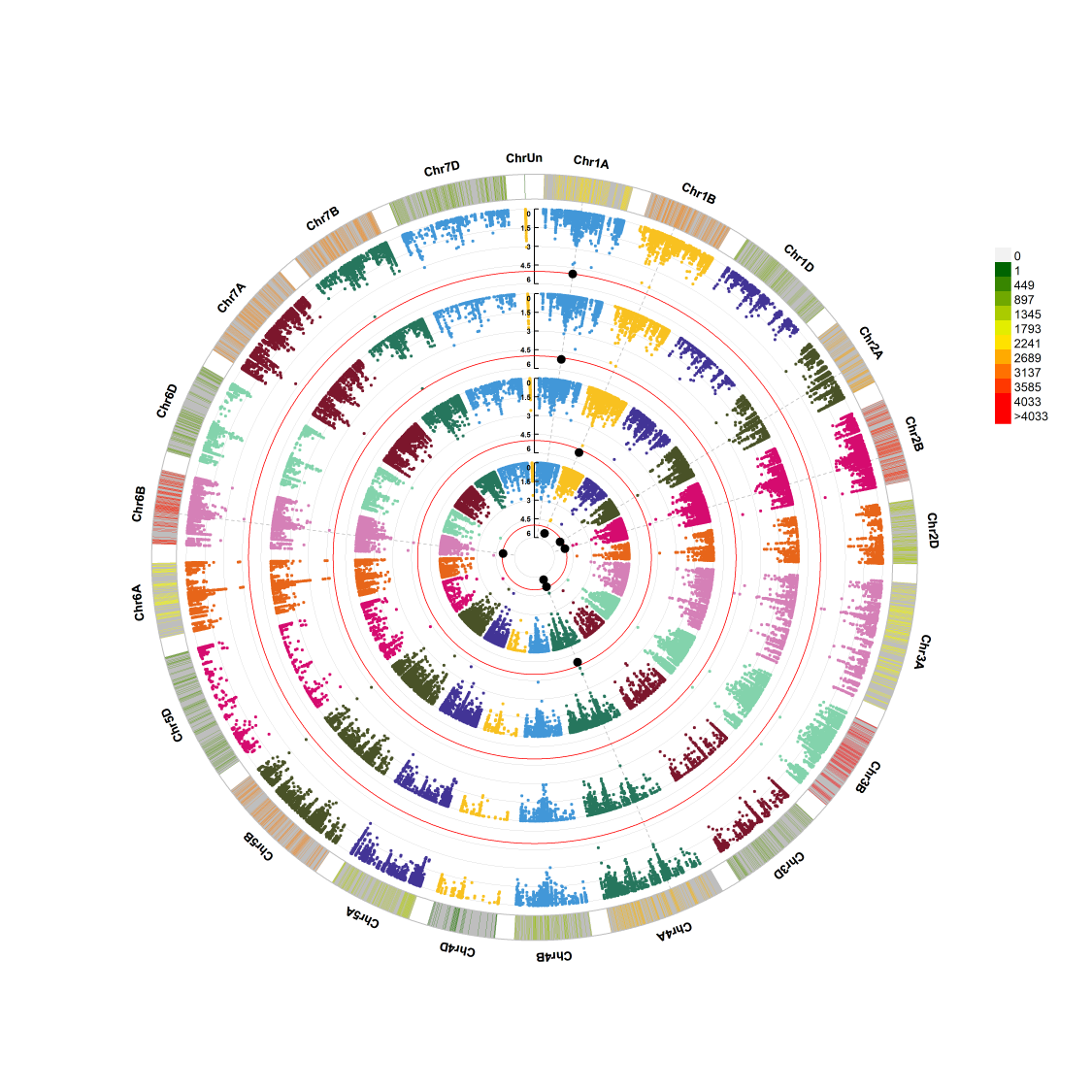 | mrMLM well-watered MLM well-watered   \| 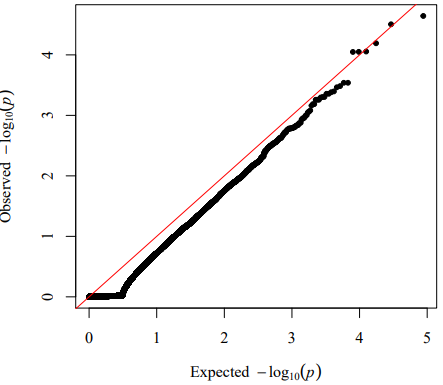  mrMLM rain-fed \| 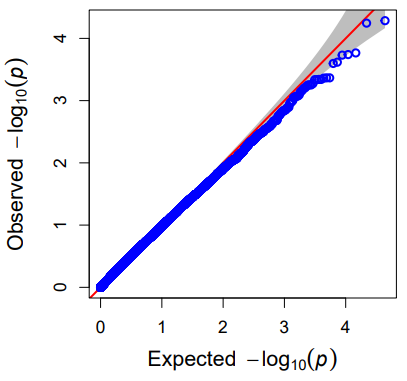  MLM rain-fed \| \| --- \| --- \| \| 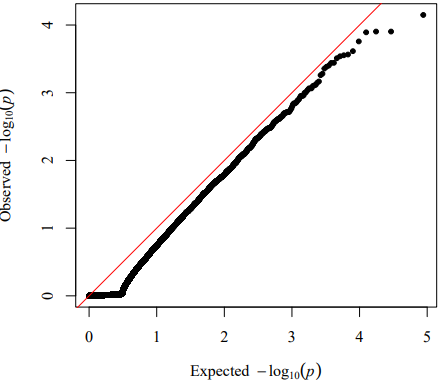 \| 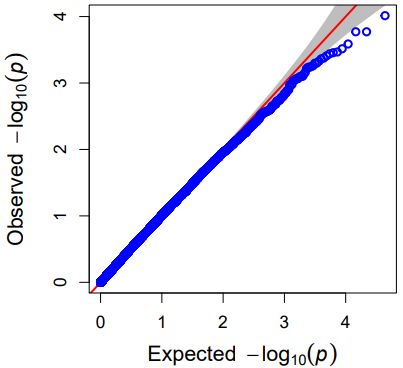 \| |
| PerEquivD |  |
| 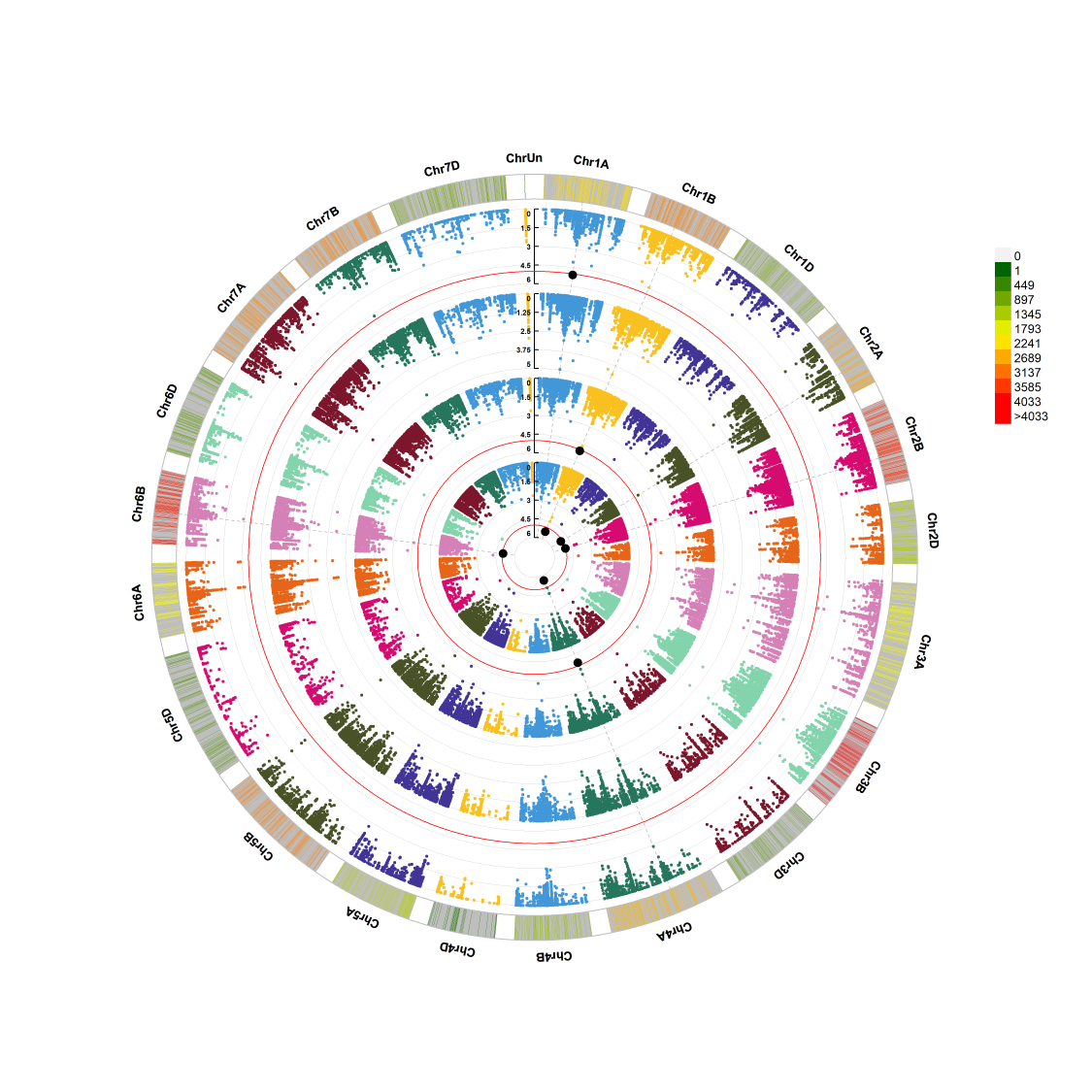 | mrMLM well-watered MLM well-watered   \| 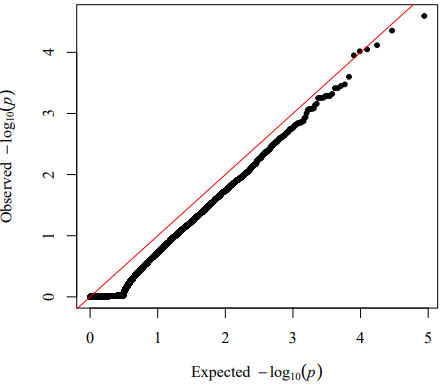  mrMLM rain-fed \| 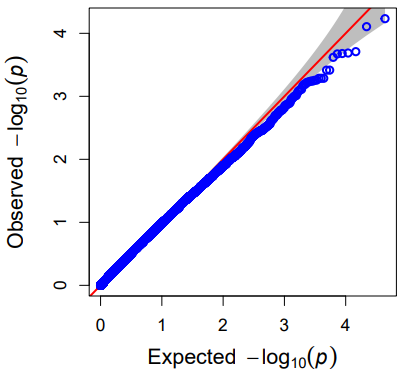  MLM rain-fed \| \| --- \| --- \| \|  \|  \| \| 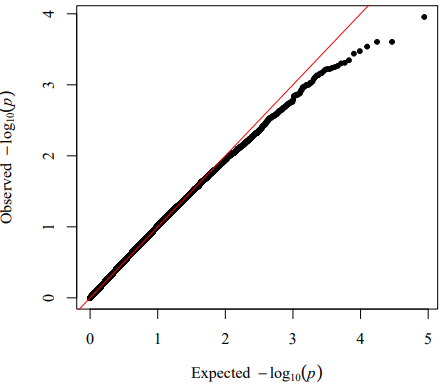 \| 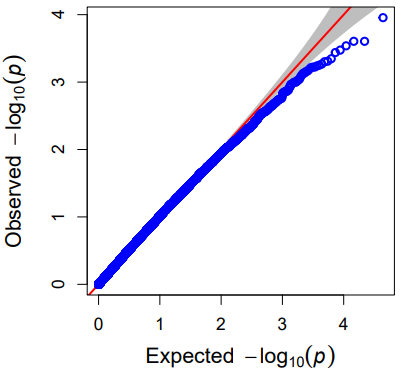 \| |
| EquivEllAr |  |
| 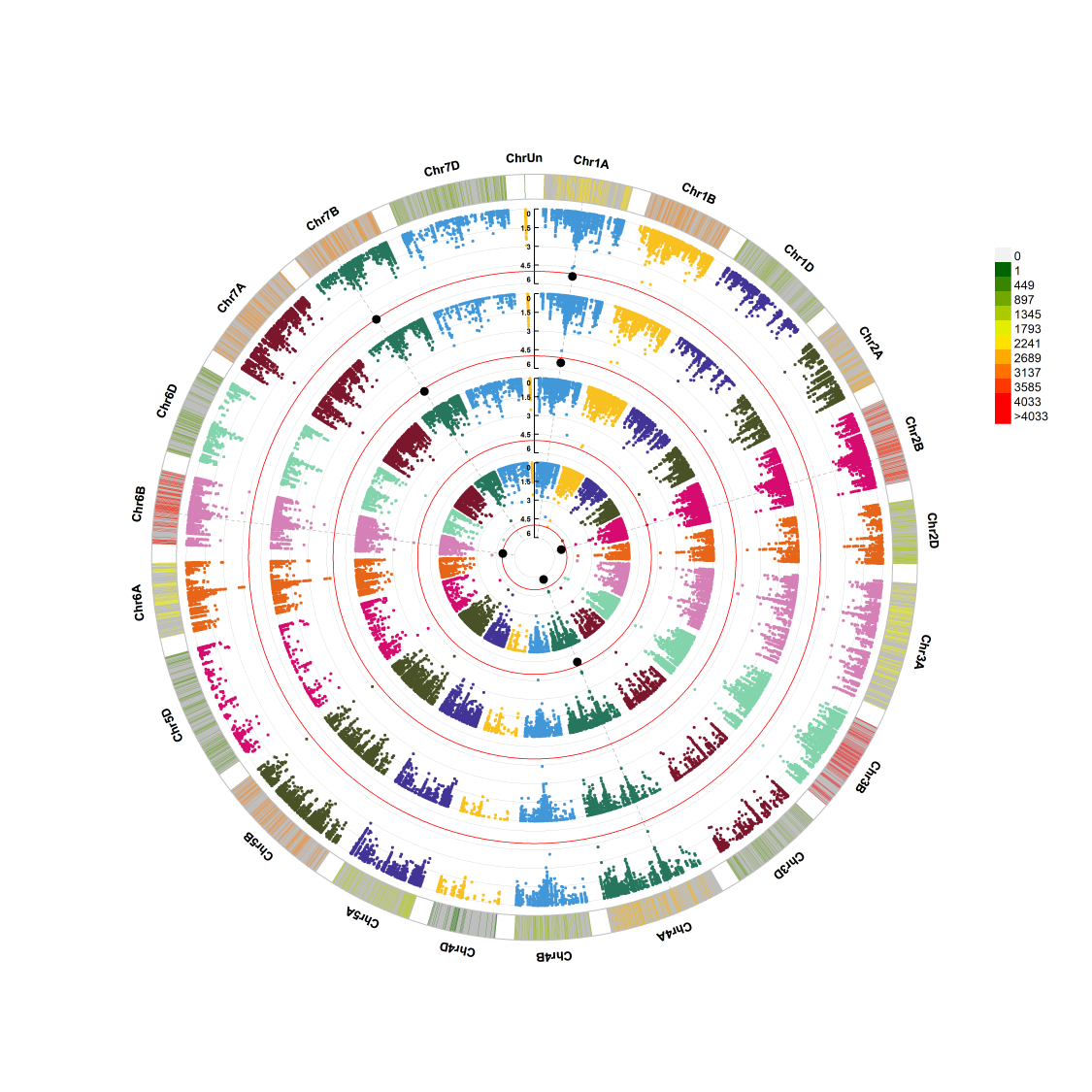 | mrMLM well-watered MLM well-watered   \| 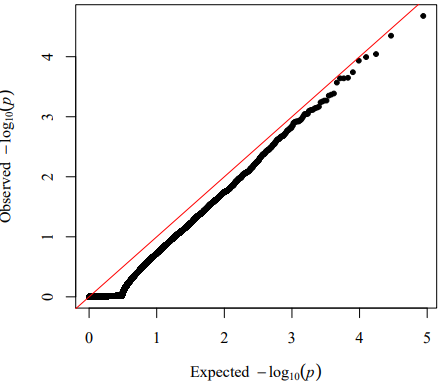  mrMLM rain-fed \| 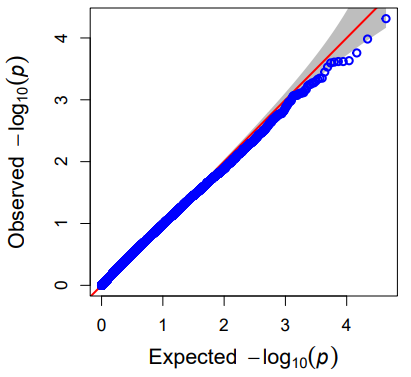 \| \| --- \| --- \| \|  \| MLM rain-fed \| \| 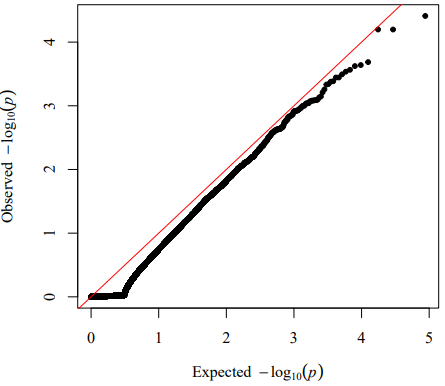 \| 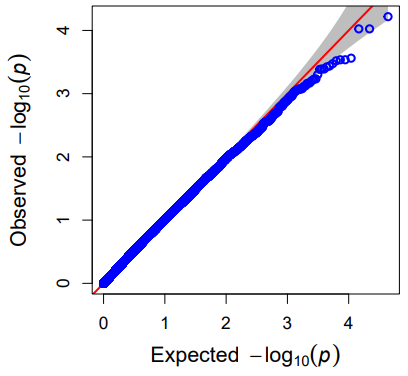 \| |
| Compactness | mrMLM well-watered MLM well-watered |
| 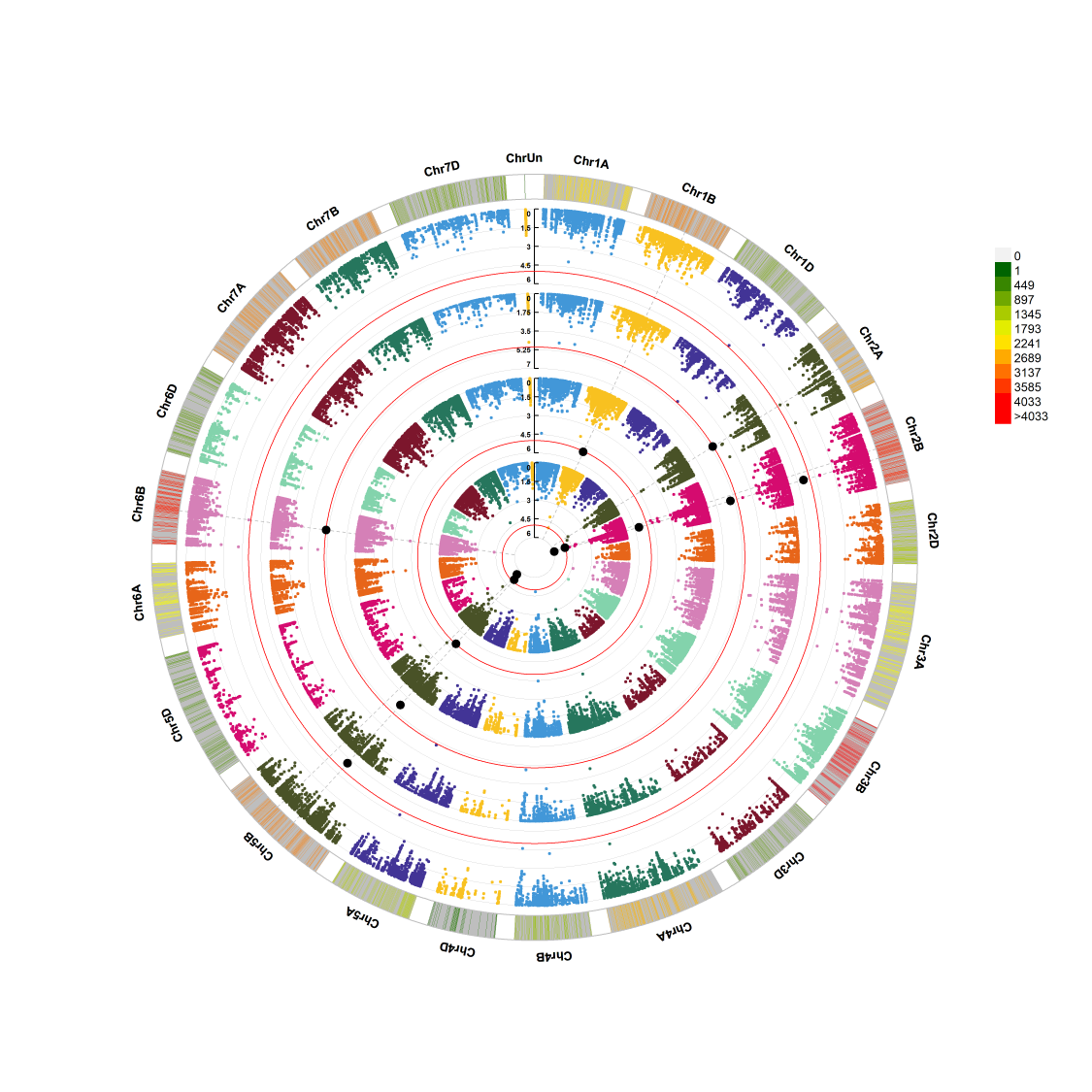 | \| 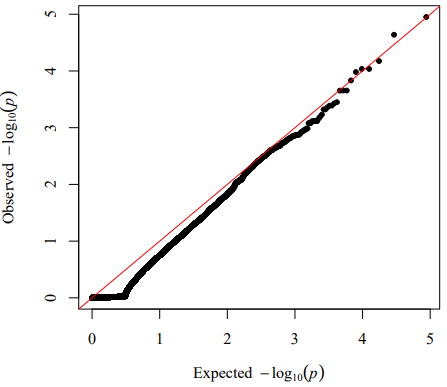  mrMLM rain-fed \| 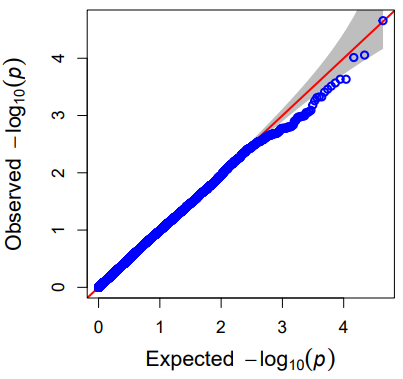 \| \| --- \| --- \| \|  \| MLM rain-fed \| \| 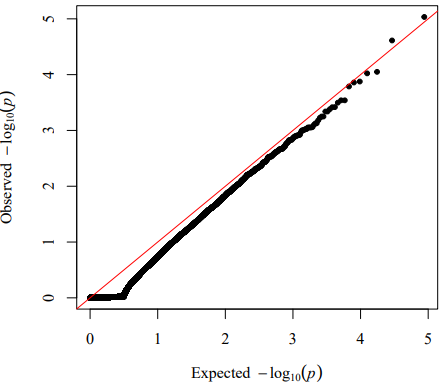 \| 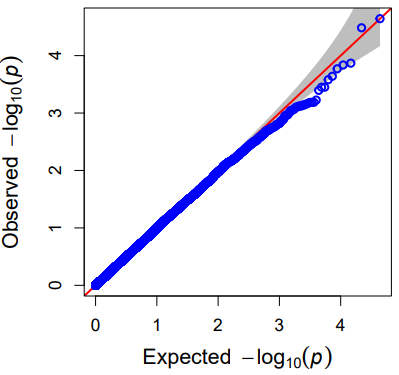 \| |
| Solidity | mrMLM well-watered MLM well-watered |
| 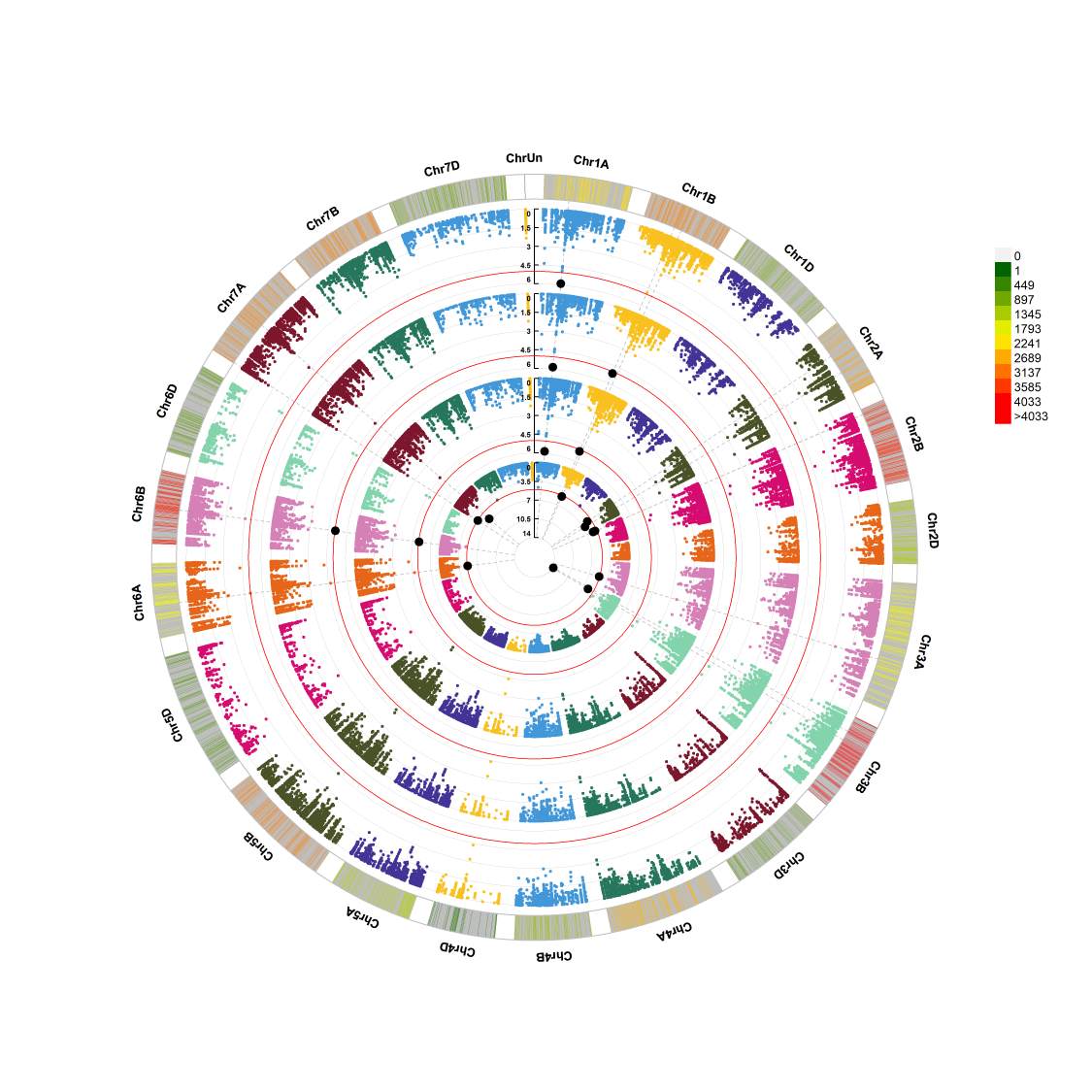 | \| 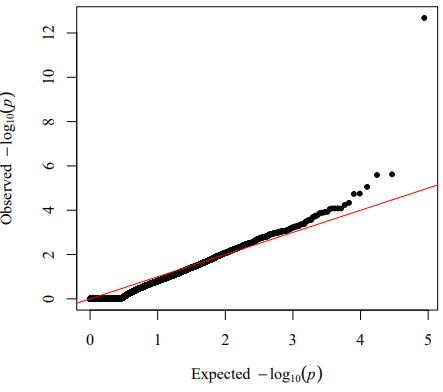  mrMLM rain-fed \| 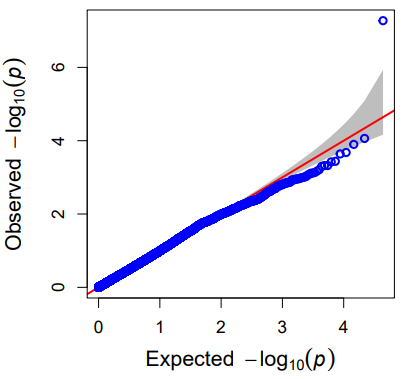  MLM rain-fed \| \| --- \| --- \| \|  \|  \| \| 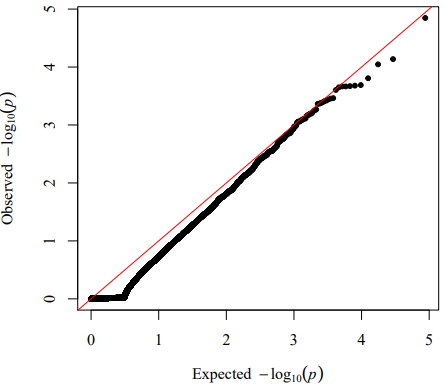 \| 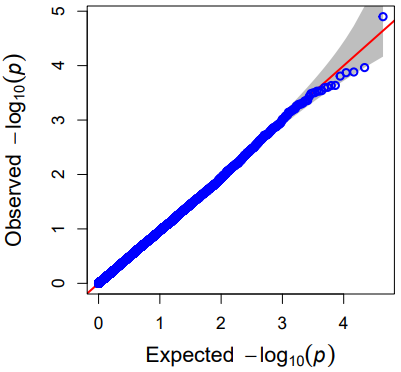 \| |
| Concavity | mrMLM well-watered MLM well-watered |
| 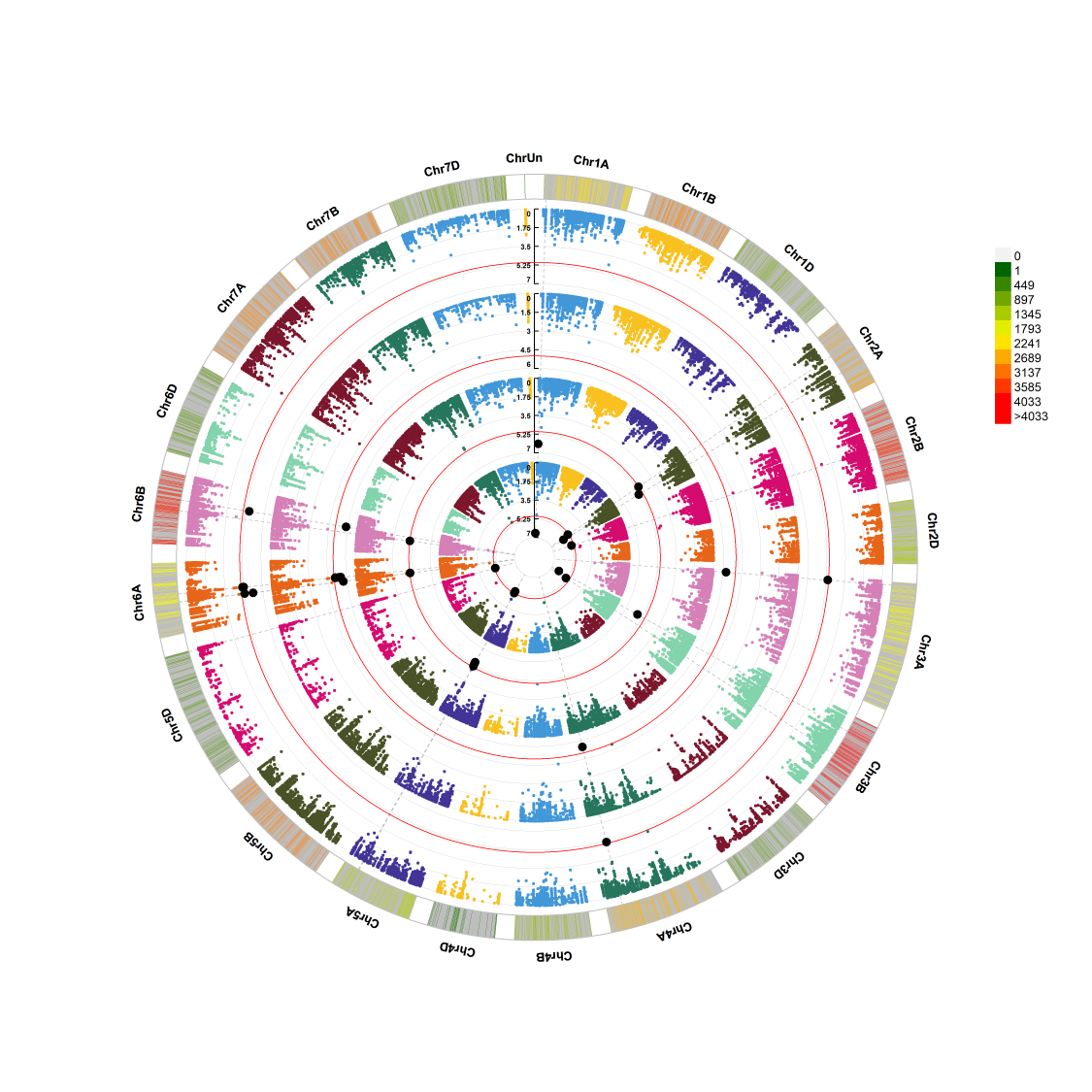 | \| 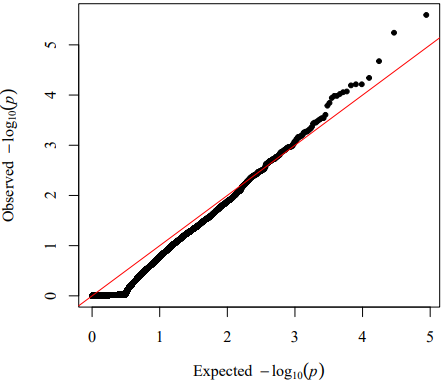  mrMLM rain-fed \| 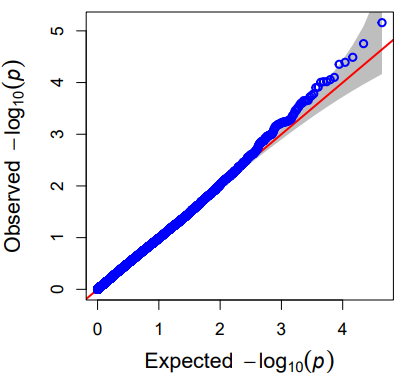  MLM rain-fed \| \| --- \| --- \| \| 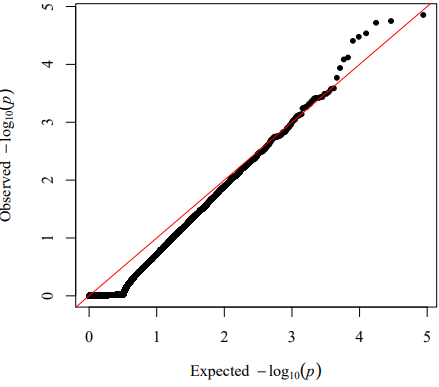 \| 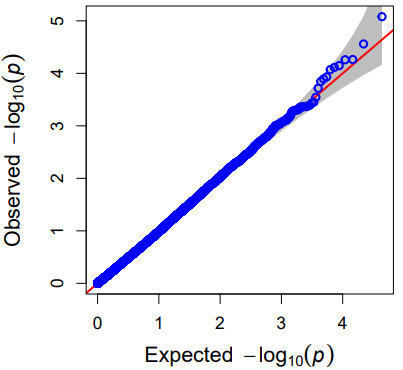 \| |
| Convexity | mrMLM well-watered MLM well-watered |
| 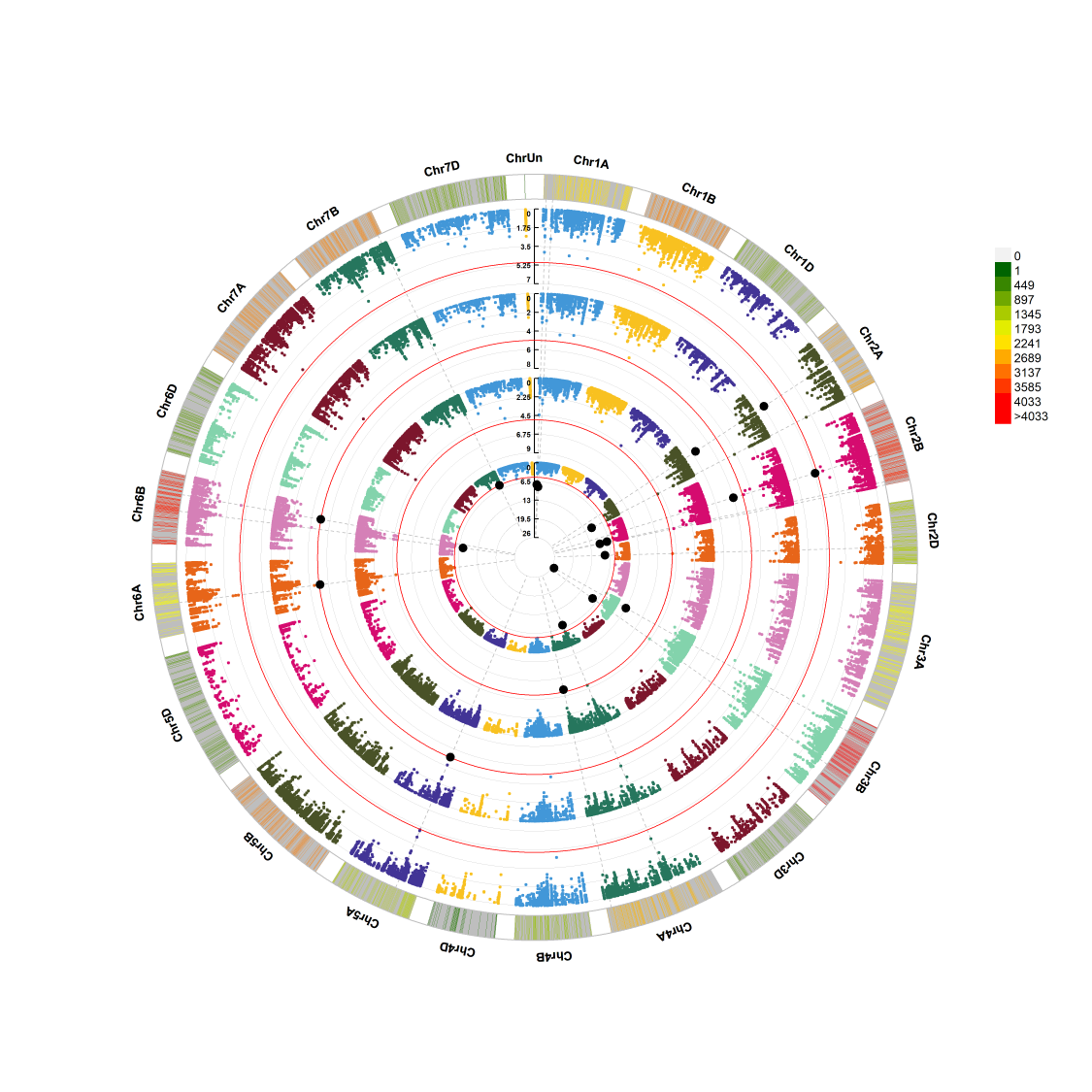 | \| 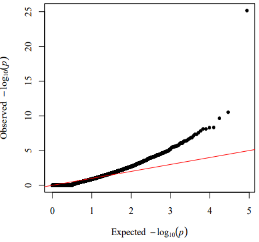  mrMLM rain-fed \| 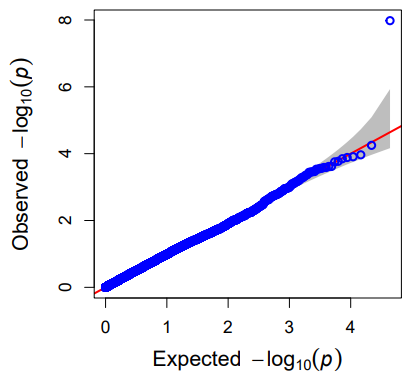  MLM rain-fed \| \| --- \| --- \| \|  \|  \| |
| Shape | mrMLM well-watered MLM well-watered |
|  | \|  \|  \| \| --- \| --- \| \| mrMLM rain-fed \| MLM rain-fed \| \|  \|  \| |
| RFactor | mrMLM well-watered MLM well-watered |
|  | \|  \|  \| \| --- \| --- \| \| mrMLM rain-fed \| MLM rain-fed \| \|  \|  \| |
| ModRatio | mrMLM well-watered MLM well-watered |
|  | \|  \|  \| \| --- \| --- \| \| mrMLM rain-fed \| MLM rain-fed \| \|  \|  \| |
| Sphericity | mrMLM well-watered MLM well-watered |
|  | \|  \|  \| \| --- \| --- \| \| mrMLM rain-fed \| MLM rain-fed \| \|  \|  \| |
| Rectang | mrMLM well-watered MLM well-watered |
|  | \|  \|  \| \| --- \| --- \| \| mrMLM rain-fed \| MLM rain-fed \| \|  \|  \| |
| Perim.1 | mrMLM well-watered MLM well-watered |
|  | \|  \|  \| \| --- \| --- \| \| mrMLM rain-fed \| MLM rain-fed \| \|  \|  \| |
| Area.1 | mrMLM well-watered MLM well-watered |
|  | \|  \|  \| \| --- \| --- \| \| mrMLM rain-fed \| MLM rain-fed \| \|  \|  \| |
| ArBBox.1 | mrMLM well-watered MLM well-watered |
|  | \|  \|  \| \| --- \| --- \| \| mrMLM rain-fed \| MLM rain-fed \| \|  \|  \| |
| Thickness | mrMLM well-watered MLM well-watered |
|  | \|  \|  \| \| --- \| --- \| \| mrMLM rain-fed \| MLM rain-fed \| \|  \|  \| |
| Perim.2 | mrMLM well-watered MLM well-watered |
|  | \|  \|  \| \| --- \| --- \| \| mrMLM rain-fed \| MLM rain-fed \| \|  \|  \| |
| Area.2 | mrMLM well-watered MLM well-watered |
|  | \|  \|  \| \| --- \| --- \| \| mrMLM rain-fed \| MLM rain-fed \| \|  \|  \| |
| ArBBox.2 | mrMLM well-watered MLM well-watered |
|  | \|  \|  \| \| --- \| --- \| \| mrMLM rain-fed \| MLM rain-fed \| \|  \|  \| |
| Volume | mrMLM well-watered MLM well-watered |
|  | \|  \|  \| \| --- \| --- \| \| mrMLM rain-fed \| MLM rain-fed \| \|  \|  \| |
|  |  |

**Supplementary Fig 2.** Circular Manhattan (A) and QQ-plots (B) to draw common regions associated with seed traits in Iranian wheat landraces and cultivars. Inner to outer circles represents average trait for the FarmCPU and GLM methods in the well-watered and rain-fed environments, respectively. The chromosomes are plotted at the outmost circle where thin dotted blue ‎and red lines indicate significant levels at P-value<0.00001 (0.05/m, Bonferroni), respectively. Black dots indicate genome-‎wide significantly associated SNPs at P-value<0.00001 (0.05/m , Bonferroni)), probability levels. The scale between ChrUn ‎and Chr1A indicates − log10 (p) values. Colored boxes outside on the top right side indicate SNP density across the genome ‎where green to red indicates less dense to dense.‎

**Supplementary Fig 3.** The KEGG pathway of metabolic pathways.

**Supplementary Fig 4.** The KEGG pathway of ubiquitin-mediated proteolysis.

**Supplementary Fig 5.** The KEGG pathway of oxidative phosphorylation.

**Supplementary Fig 6.** The KEGG pathway of carbon metabolism.

**Supplementary Fig 7.** The KEGG pathway of pentose phosphate.

**Supplementary Fig 8.** The KEGG pathway of sulfur metabolism.

**Supplementary Fig 9.** The KEGG pathway of fatty acid elongation.

The pathway map without coloring is the original version that is manually drawn by in-house software called KegSketch. The other pathway maps with coloring are all computationally generated as summarized below.

Reference pathway: this is the original version; white boxes are hyperlinked to KO, ENZYME, and REACTION entries in metabolic pathways; they are hyperlinked to KO entries in non-metabolic pathways.

Reference pathway (KO): blue boxes are hyperlinked to KO entries that are selected from the original version.

Reference pathway (EC): blue boxes are hyperlinked to ENZYME entries that are selected from the original vrsion.

Reference pathway (Reaction): blue boxes are hyperlinked to REACTION entries that are selected from the original version.

Organism-specific pathway: green boxes are hyperlinked to GENES entries by converting K numbers (KO identifiers) to gene identifiers in the reference pathway, indicating the presence of genes in the genome and also the completeness of the pathway.
